# Supplementary material for: Single-cell long-read transcriptomics: from technologies to biological insights
Source: Gigascience. 2026 Jul 22;15:giag082. doi: 10.1093/gigascience/giag082 (PMC13508701; doi:10.1093/gigascience/giag082)
Supplement: Supplementary file 1 [file giag082_authors_response_to_reviewer_comments_original_submission.pdf]

Author's Response To Reviewer Comments

Reviewer1
This is a well-structured and timely review that covers the scLR-seq landscape comprehensively. It will be a useful reference for the field, though several areas need strengthening before publication.
Response:
We thank the reviewer for the positive assessment of our review. We also appreciate the reviewer's constructive comments and have carefully revised the manuscript to strengthen the areas highlighted below. We also strengthened the previously underdeveloped sections, added quantitative benchmarking evidence, and improved the balance and organization of the manuscript.

Strengths:
The review covers the full pipeline from sequencing platforms to biological applications in a logical sequence. The inclusion of four detailed comparison tables (library strategies, demultiplexing tools, transcript discovery tools, functional analysis tools) is genuinely useful and not commonly found consolidated in a single review. Figures 1-4 are clear and well-designed.
Response:
We thank the reviewer for the positive comments on the organization, tables, and figures. Following Reviewer 2's suggestion, we have further consolidated the original Figures 2 and 3 into a single integrated figure, as these two figures presented related information that could be more effectively communicated together. This revision reduces redundancy and improves the clarity and coherence of the visual presentation.

Weaknesses:
1. There is a clear copy-paste error on lines 59-60: "These platforms have effectively . These platforms have effectively overcome the fragmentation issues…" More broadly, several sentences in the Background are overly long and would benefit from restructuring for clarity.
Response:
We thank the reviewer for identifying this editing error. We have removed the repeated phrase and revised the Background to improve sentence structure and readability. In the revised manuscript, the Background now more directly explains why short-read scRNA-seq is insufficient for resolving full-length transcript structures and how long-read sequencing addresses these limitations. We also shortened several overly long sentences to make the introduction easier to follow. These changes have been made in the Background section (line 40-76).

2. Cell Type Annotation Section is Underdeveloped - The discussion of cell type annotation is notably leaner than other sections. The authors correctly identify the gap, that isoform-level annotation frameworks are lacking, but offer only a brief speculatiion on future directions.
Response:
We agree with the reviewer that the original discussion of cell type annotation was too brief. We have revised the "Cell Type Annotation" section to provide a more comprehensive and balanced discussion of current annotation strategies, their limitations in the context of single-cell long-read transcriptomics, and potential future directions (line 348-368). Specifically, we now clarify that most existing annotation frameworks were developed for short-read scRNA-seq and operate primarily at the gene-expression level, whereas scLR-seq can capture isoform-level features, including alternative splicing and transcript boundary usage, that may contain cell-type-specific information. We also expanded the discussion of future developments by highlighting the need for multi-layered annotation strategies, isoform-aware reference resources, validated catalogs of cell-type-specific isoforms, and benchmarking frameworks to evaluate platform reliability and annotation robustness. These revisions provide a more detailed account of both the current gap and the methodological directions needed to support isoform-based cell type annotation.

3. Computational Challenges Section Needs Quantitative Grounding - The challenges discussion reads somewhat qualitatively. Phrases like "relatively high base-level error rates" and "lower in effective per-cell transcript coverage" would benefit from concrete numbers (ONT for example was often previously seen as very error prone but that's no longer the case - more recent papers have these numbers), at least as approximate benchmarks, to help readers appreciate the magnitude of the problems. Citing the systematic benchmarking studies already referenced more specifically here would help.
Response:
We agree with the reviewer that the original challenges discussion was too qualitative. We have revised the computational sections to include more quantitative context from recent benchmarking and systematic evaluation studies. Specifically, in the "Cell Barcode and UMI Demultiplexing" section, we now state that nanopore raw-read accuracy has improved from approximately 95% with earlier R9 chemistries to over 99% with recent R10.4.1 chemistry, while emphasizing that a typical 10-20 bp cell barcode can still have an estimated 10-18% probability of containing at least one sequencing error. We also added benchmark-based discussion of barcode recovery performance, including the approximate 92% recall of matched short-read reference barcodes across evaluated methods, together with method-specific trade-offs in precision, recall, UMI correction, and sequencing chemistry. In the "Transcript Discovery and Quantification" section, we further added quantitative and benchmark-based discussion of barcode assignment rates, per-cell read depth, truncation bias, and the relative contributions of read length, read accuracy, and sequencing depth to transcript model reliability. These revisions provide a more concrete assessment of the magnitude of current computational challenges and are included in line 245-284 and line 287-339.

4. Inconsistent Depth Across Biological Applications - The immunology subsection is noticeably more developed than neuroscience and oncology. The neurodevelopment discussion leans heavily on a single study (ScISOr-Seq2), and the oncology discussion is brief. The abstract promises substantial coverage of all three domains; the body of the review should be improved.
Response:
We thank the reviewer for pointing out this imbalance. We have revised the applications section to improve the balance among immunology, neurodevelopment, and oncology, and to better match the scope promised in the Abstract. The revised section now presents these applications as examples of how scLR-seq can reveal transcript-structure variation across biological systems rather than as an uneven list of individual studies. We expanded the neurodevelopment discussion beyond a single study by emphasizing cell-type-resolved splicing, promoter usage, and polyadenylation regulation in neural systems, and we expanded the oncology discussion to highlight how fusion transcripts, allele-specific expression, somatic variants, and isoform diversity can contribute to tumor heterogeneity and disease interpretation. These revisions are located in the "Applications in Immunology and Beyond" section (line 567-620).

5. Multi-omics Integration is Underrepresented - Both the abstract and cover letter emphasize multi-omics integration as a key future direction, yet the actual discussion is dispersed and thin. A dedicated subsection or at minimum a summary table of tools like SPLONGGET, ScISOr-ATAC, and scGTP-seq, would make this a more actionable resource for readers planning such experiments.
Response:
We thank the reviewer for this helpful comment. We agree that multi-omics integration required more explicit treatment. We have added a dedicated "Multi-omics integration" subsection to consolidate the discussion that was previously dispersed across the manuscript (line 542-565). The revised subsection now explains why integrating full-length transcript information with chromatin, spatial, genomic, and other single-cell modalities is important for interpreting cell-state regulation and disease mechanisms. We also discuss recent long-read single-cell multi-omics approaches, including scNanoCOOL-seq, SPLONGGET, and ScISOr-ATAC, and clarify that the field still lacks mature library preparation protocols and standardized computational pipelines for joint interpretation of transcript expression, isoform usage, chromatin accessibility, and genetic variation (line 556-559). In addition, we expanded the Table 5 to include multi-omics methods such as scNanoCOOL-seq, SPLONGGET, ScISOr-ATAC, and scGTP-seq, thereby making the revised manuscript more useful as an actionable resource for readers planning multi-omics experiments.

6. Missing Critical Discussion: Benchmarking and Reproducibility - The review mentions benchmark studies but does not synthesise what those benchmarks actually found. A paragraph summarizing key take-home messages from existing benchmarks would substantially increase the practical value of this review.
Response:
We agree with the reviewer and have expanded the benchmarking and reproducibility discussion to provide clearer synthesis rather than simply citing existing benchmark studies. In the revised manuscript, we now summarize key take-home messages from available benchmarks and quantitative evaluations across the platform-selection and computational-analysis workflow. In the "Long-Read Platform Selection for scLR-seq" section, we added a more explicit comparison of ONT and PacBio HiFi as complementary platforms and framed platform choice as a multi-parameter optimization problem involving per-read accuracy, molecule throughput per cell, barcode/UMI robustness, and cost per informative transcript. In the "Cell Barcode and UMI Demultiplexing" section, we discuss benchmark findings on cell barcode and UMI recovery, method-specific differences in sensitivity and precision, and the influence of sequencing chemistry on UMI correction. In the "Transcript Discovery and Quantification" section, we summarize benchmark evidence indicating that read length and read accuracy can affect transcript model reliability more strongly than sequencing depth alone, and we further discuss the trade-off between discovery breadth and model specificity. We also expanded the "Challenges and Future Directions" section to address reproducibility more explicitly, noting that novel isoform discovery requires careful validation, that isoform-level signals are sparser than gene-level counts, and that public benchmark datasets, unified evaluation metrics, and standardized cross-platform protocols remain essential infrastructure for the field. These revisions appear in lines 93-143, line 245-284, line 287-345 and line 622-656.

7. RNA Modifications Section is Abrupt - The paragraph introducing m6A-isoSC-seq and scm6A-seq appears mid-section on "Complex Transcripts" and feels appended rather than integrated. RNA modifications arguably deserve either a dedicated subsection or a more deliberate placement with proper framing.
Response:
We sincerely thank the reviewer for this insightful comment. We have reorganized the manuscript so that RNA modifications are now discussed in a dedicated "RNA Modifications" subsection rather than appearing abruptly within the discussion of complex transcripts (line 456-479). The revised section first introduces m6A as an additional regulatory layer of transcriptome biology and explains why bulk approaches can obscure cell-to-cell heterogeneity in RNA modification profiles. We then discuss current single-cell long-read strategies for m6A detection, such as m6A-isoSC-seq, in a more deliberate methodological context. We also added clearer framing of current limitations, including cDNA dependence, barcode retention, sensitivity, throughput, and the need for future methods that can jointly measure transcript structure and RNA modification in individual cells.

Reviewer2
The manuscript by Ren et al presents an overview of the single cell long reads transcriptomics approaches. The manuscript is timely owing to the rapid development of the technologies and field of applications. Long read transcriptomics has massive potential however as an extremely rapidly developing field it can be difficult to access for a non expert. The availability of reviews of the field including technologies, libraries preparation, and computational approaches can contribute in democratizing such approaches. In the current manuscript the authors present a thorough and well organised overview of long-read single-cell transcriptomics approaches.
Response:
We sincerely thank the reviewer for the positive and constructive assessment of our manuscript. We are encouraged that the reviewer recognizes the timeliness of this topic and the potential value of a review that summarizes technologies, library preparation strategies, and computational approaches for single-cell long-read transcriptomics. In response to the reviewer's detailed comments below, we have comprehensively revised the manuscript to improve its focus, organization, accessibility, and practical value for both specialists and non-expert readers. The overall scope of the review remains unchanged, but the revised version now provides a more coherent framework for platform selection, library design, computational analysis, benchmarking, biological applications, and future directions.

Nevertheless, the manuscript is currently very verbose and the target audience is unclear, the work is too general / unfocussed for an experienced audience, but includes perhaps more than necessary do the detriment of it's length for non-specialist audience. The manuscript would gain in being substantially shortened and more focused. For instance figures 2 and 3 are rather uninformative for the objective of the manuscript. Those present the sequencing technologies and integration.
Response:
We sincerely thank the reviewer for this insightful comment. The observation is highly accurate. During the initial drafting, the manuscript gradually shifted toward a method-by-method listing, which deviated from our original intent.
In reversion, we have revised the manuscript to provide a clear, concise overview of single-cell long-read transcriptomics that emphasizes the overall logic of the field and highlights key challenges, with the goal of helping scRNA-seq researchers rapidly understand current approaches. Specifically, we removed detailed method descriptions and consolidated similar methods into broader categories. For each section, we clarified the critical issues that researchers need to consider. Figures 2 and 3 have been integrated and redesigned to represent a decision-making workflow, allowing readers to quickly select appropriate platforms and single-cell long-read strategies. We believe these revisions substantially improve focus, readability, and practical value for the intended audience.

Likewise the authors should pay close attention to the contents of the tables, as such Table 1 that is focusing on library constructions, also include software and pipelines (Sockeye, scNonoGPS, Flexiplex) obfuscating the message from this table. This is also reflected in the text as lines 232-240 deal with algorithms in a section focusing on scLR-Seq library construction. I invite the authors in bringing clearer structure to the manuscript as this will bring greater focus and potential for the readers.
Response:
We thank the reviewer for this comment. We recognize that Table 1 previously mixed library construction protocols with associated computational methods, which could obscure the message. In the original draft, we found this difficult to fully disentangle. Single-cell long-read approaches that do not rely on short-read data follow two main strategies, either optimizing barcode design or optimizing computational methods. And new computational methods often come with specific library design considerations. As a result, Table 1 originally included some library schemes associated with computational method optimization.
To address this concern, we have revised the manuscript to clarify this structure, and all computational-method-associated library schemes have now been moved to the "Computational Methods" section for further discussion, making the separation between library construction and algorithmic optimization clearer.

Specific Comments
- Line 60. Repeated and meaningless sentence with incorrect citations. Assuming an editing error.
Response:
We thank the reviewer for catching this editing error. We have removed the repeated sentence and checked the surrounding citations for consistency. This correction was made in the Background section. We also reviewed the manuscript more broadly to identify similar editing artifacts.

- Line 89-92. Has AI been used in this document? This has a language stands out quite differently to the surrounding copy.
Response:
We thank the reviewer for this comment. The content reflects our intended message: single-cell long-read sequencing is no longer merely a supplement to short-read approaches, but can independently resolve questions that short reads cannot, such as detecting alternative splicing, fusion genes, allele-specific expression, and other transcriptomic features relevant in immunology, cancer, and related fields. Because it integrates multiple layers of transcriptomic information the description emphasizes the broader impact, which is why the wording is more expansive. All scientific content and conclusions are authored by us and have been reviewed for accuracy and consistency with the overall manuscript.
We have also provided a summary of AI-assisted writing in the "Declaration of Generative AI and AI-

assisted Technologies in the Manuscript Preparation Process” (line 686-690).

- Fig1. Editing box around "Allele expression Across Cells". The document has a large number of mistakes and errors like this suggesting closer attention to editing is required.
Response:
We apologize for the editing oversight and thank the reviewer for pointing it out. We have corrected the Figure 1 labeling issue around “Allele expression Across Cells” and reviewed the figure for similar formatting problems. In addition, we performed a broader language and formatting check of the manuscript to remove repeated phrases, inconsistent wording, and residual editing artifacts. We have also carefully inspected the final Word and PDF proofs to ensure that the figures, tables, and text are clean and consistent before resubmission.

Editorial Manager®

Cookie Preference Center

We use cookies which are necessary to make our site work. We may also use additional cookies to analyze, improve and personalize our content and your digital experience. For more information, see our Cookie Policy.

You may choose not to allow some types of cookies. However, blocking some types may impact your experience of our site and the services we are able to offer. See the different category headings below to find out more or change your settings.

Allow all

Manage Consent Preferences

Strictly Necessary Cookies

Always active

These cookies are necessary for the website to function and cannot be switched off in our systems. They are usually only set in response to actions made by you which amount to a request for services, such as setting your privacy preferences, logging in or filling in forms. You can set your browser to block or alert you about these cookies, but some parts of the site will not then work.

Functional Cookies

These cookies enable the website to provide enhanced functionality and personalisation. They may be set by us or by third party providers whose services we have added to our pages. If you do not allow these cookies then some or all of these services may not function properly.

Performance Cookies

These cookies allow us to count visits and traffic sources so we can measure and improve the performance of our site. They help us to know which pages are the most and least popular and see how visitors move around the site.

Targeting Cookies

These cookies may be set through our site by our advertising partners. They may be used by those companies to build a profile of your interests and show you relevant adverts on other sites. If you do not allow these cookies, you will experience less targeted advertising.

Cookie List

Clear

Apply Cancel

Consent Leg.Interest

Confirm my choices

Power
