# Supplementary material for: Single-cell long-read transcriptomics: from technologies to biological insights
Source: Gigascience. 2026 Jul 22;15:giag082. doi: 10.1093/gigascience/giag082 (PMC13508701; doi:10.1093/gigascience/giag082)
Supplement: Supplementary file 2 [file giag082_authors_response_to_reviewer_comments_revision_1.pdf]

Author's Response To Reviewer Comments

Reviewer #1: All comments addressed, the only final comment is figure 2, top right on the PacBio figure: hig-confidence should be high-confidence
Response:
We thank the reviewer for identifying this typographical error. We have corrected "hig-confidence" to "high-confidence" in the PacBio panel in the upper-right portion of Figure 2.

Reviewer #2: I am grateful to the authors for the extensive revision of the manuscript. As previously the filed is rapidly growing and the review is extremely timely. The authors have answered th epoints previously raised. however in the revised version of the manuscripts there are some concerns that need addressing.
Response:
We sincerely thank the reviewer for the positive assessment of our revised manuscript and for recognizing the timeliness of this review. We also appreciate the reviewer's careful evaluation of the revised version. We have carefully considered the remaining concerns and have further revised the manuscript accordingly. Detailed responses to each specific comment are provided below.

Line 123 - 127 "Because PacBio CCS accuracy is achieved by repeated sequencing of the same molecule, the number of independent molecules recovered per unit of sequencing output and cost should be carefully considered. This issue is especially relevant for large cell numbers, low-abundance isoforms, shallow per-cell coverage, and studies requiring broad transcriptome discovery". I do not agree with this statement in our hands the two technologies (PacBio, and ONT) yielded similar number of reads (after deconcatenation for PacBio) and similar number of reads after de duplication. I invite the authors to be cautious with their statement, and either add data or references to support this or rvise.
Response:
We thank the reviewer for this important clarification and agree that our original statement was overly general and did not adequately reflect recent advances in PacBio single-cell long-read sequencing workflows. The concern regarding reduced recovery of independent molecules was more applicable to earlier PacBio implementations. With the introduction of multiplexed concatenation strategies, including MAS-Seq/Kinnex-based workflows, current PacBio and ONT approaches can achieve comparable recovery of deduplicated molecules in single-cell applications.

We have therefore removed the original statement and revised this section to avoid implying an inherent disadvantage of PacBio in molecular recovery. The revised text now focuses on the remaining practical and platform-specific differences, including library-processing complexity, scalability and flexibility, cost at a given batch size, and per-read accuracy.
The revised text reads:
(Lines 123-125): "However, PacBio Kinnex-based scLR-seq involves programmed cDNA concatenation followed by computational read segmentation, introducing additional platform-specific steps in library preparation and primary data processing [1]."
(Lines 138-142): "Current PacBio and ONT workflows can achieve comparable recovery of deduplicated molecules in single-cell applications [2, 3]. The remaining distinctions between the two platforms primarily involve library-processing biases and complexity, sequencing scalability and operational flexibility, batch-dependent cost, and per-read accuracy."

[1] Al'Khafaji AM, Smith JT, Garimella KV, et al. High-throughput RNA isoform sequencing using programmed cDNA concatenation. Nat Biotechnol 2024;42(4):582-586. doi:10.1038/s41587-023-01815-7.
[2] Scoones ALA, Lan Y, Utting C, et al. A comparison of long-read single-cell transcriptomic approaches. bioRxiv 2025. doi:10.1101/2025.07.03.662955.
[3] Deng E, Shen Q, Zhang J, et al. Systematic evaluation of single-cell RNA-seq analyses performance based on long-read sequencing platforms. J Adv Res 2025;71:141-153. doi:10.1016/j.jare.2024.05.020.

Cookie Preference Center

We use cookies which are necessary to make our site work. We may also use additional cookies to analyze, improve and personalize our content and your digital experience. For more information, see our Cookie Policy.

You may choose not to allow some types of cookies. However, blocking some types may impact your experience of our site and the services we are able to offer. See the different category headings below to find out more or change your settings.

Allow all

Manage Consent Preferences

Strictly Necessary Cookies

Always active

These cookies are necessary for the website to function and cannot be switched off in our systems. They are usually only set in response to actions made by you which amount to a request for services, such as setting your privacy preferences, logging in or filling in forms. You can set your browser to block or alert you about these cookies, but some parts of the site will not then work.

Functional Cookies

These cookies enable the website to provide enhanced functionality and personalisation. They may be set by us or by third party providers whose services we have added to our pages. If you do not allow these cookies then some or all of these services may not function properly.

Performance Cookies

These cookies allow us to count visits and traffic sources so we can measure and improve the performance of our site. They help us to know which pages are the most and least popular and see how visitors move around the site.

Targeting Cookies

These cookies may be set through our site by our advertising partners. They may be used by those companies to build a profile of your interests and show you relevant adverts on other sites. If you do not allow these cookies, you will experience less targeted advertising.

Cookie List

Clear

Apply Cancel

Consent Leg.Interest

Confirm my choices

Power
