# Supplementary material for: Single-cell long-read transcriptomics: from technologies to biological insights
Source: Gigascience. 2026 Jul 22;15:giag082. doi: 10.1093/gigascience/giag082 (PMC13508701; doi:10.1093/gigascience/giag082)

GigaScience

Single-cell Long-read Transcriptomics: From Technologies to Biological Insights

--Manuscript Draft--

**Manuscript Number:**	GIGA-D-26-00122
**Full Title:**	Single-cell Long-read Transcriptomics: From Technologies to Biological Insights
**Article Type:**	Review
**Funding Information:**	
**Abstract:**	The rapid advancement of single-cell long-read transcriptomics (scLR-seq) is transforming transcriptomic research. Unlike traditional approaches, scLR-seq enables the direct capture of full-length transcript structures, isoform usage patterns, and precise information on alternative splicing and transcription start and end sites at single-cell resolution. These capabilities provide an unprecedented molecular view for dissecting cellular heterogeneity, developmental trajectories, and disease mechanisms. This review summarizes the technological evolution of scLR-seq and its potential to illuminate transcriptomic complexity, cellular states, and regulatory networks across key biological domains, including immunology, neurodevelopment, and oncology. We provide a comprehensive overview of the end-to-end analytical workflows, spanning from raw data processing to transcript discovery and quantification. We also discuss downstream biological analyses, with emphasis on cell-type annotation and the resolution of isoform usage dynamics. Finally, we highlight applications of scLR-seq in diverse biological systems, such as the immune system, neural development, and tumor microenvironments, and consider future opportunities and challenges in integrating multi-omics data to decode cellular programs and disease evolution.
**Corresponding Author:**	Chuanyu Liu<br>BGI Group<br>shenzhen, CHINA
**Corresponding Author Secondary Information:**	
**Corresponding Author's Institution:**	BGI Group
**Corresponding Author's Secondary Institution:**	
**First Author:**	Chuanyu Liu
**First Author Secondary Information:**	
**Order of Authors:**	Chuanyu Liu
	Ze-Hui Ren
	Wenteng Liu
	Jianhua Yin
**Order of Authors Secondary Information:**	
**Additional Information:**	
**Question**	**Response**
Are you submitting this manuscript to a special series or article collection?	No
**Experimental design and statistics**<br><br>Full details of the experimental design and statistical methods used should be given	No

Powered by Editorial Manager® and ProduXion Manager® from Aries Systems Corporation

in the Methods section, as detailed in our Minimum Standards Reporting Checklist. Information essential to interpreting the data presented should be made available in the figure legends.<br><br>Have you included all the information requested in your manuscript?	
If not, please give reasons for any omissions below.<br><br>as follow-up to "**Experimental design and statistics**<br><br>Full details of the experimental design and statistical methods used should be given in the Methods section, as detailed in our Minimum Standards Reporting Checklist. Information essential to interpreting the data presented should be made available in the figure legends.<br><br>Have you included all the information requested in your manuscript?<br><br>"	Not applicable. This manuscript is a review article and does not include original experimental design or statistical analyses.
**Resources**<br><br>A description of all resources used, including antibodies, cell lines, animals and software tools, with enough information to allow them to be uniquely identified, should be included in the Methods section. Authors are strongly encouraged to cite Research Resource Identifiers (RRIDs) for antibodies, model organisms and tools, where possible.<br><br>Have you included the information requested as detailed in our Minimum Standards Reporting Checklist?	No
If not, please give reasons for any omissions below.	Not applicable. This manuscript is a review article and does not include original resources.

as follow-up to "**Resources**<br><br>A description of all resources used, including antibodies, cell lines, animals and software tools, with enough information to allow them to be uniquely identified, should be included in the Methods section. Authors are strongly encouraged to cite Research Resource Identifiers (RRIDs) for antibodies, model organisms and tools, where possible.<br><br>Have you included the information requested as detailed in our Minimum Standards Reporting Checklist?<br><br>"	
**Availability of data and materials**<br><br>All datasets and code on which the conclusions of the paper rely must be either included in your submission or deposited in publicly available repositories (where available and ethically appropriate), referencing such data using a unique identifier in the references and in the “Availability of Data and Materials” section of your manuscript.<br><br>Have you have met the above requirement as detailed in our Minimum Standards Reporting Checklist?	No
If not, please give reasons for any omissions below.<br><br>as follow-up to "**Availability of data and materials**<br><br>All datasets and code on which the conclusions of the paper rely must be either included in your submission or deposited in publicly available repositories	Not applicable. This manuscript is a review article and does not include original data.

Powered by Editorial Manager® and ProduXion Manager® from Aries Systems Corporation

(where available and ethically appropriate), referencing such data using a unique identifier in the references and in the “Availability of Data and Materials” section of your manuscript.<br><br>Have you have met the above requirement as detailed in our Minimum Standards Reporting Checklist?<br><br>"	
GigaScience has policies and guidelines in place for the use of generative AI-writing tools such as ChatGPT. If you have used such writing tools to assist with writing the manuscript this must be declared and cited in the text. Authors should not list AI-writing tools and other AI-assisted technologies as an author or co-author and should acknowledge that they are fully responsible for text generated or refined by AI-writing tools.<p><br><br>A summary of use (particularly in the introduction or among methods) needs to be included at the end of the paper, and the outputs should also be included as a supplementary file hosted in GigaDB or other open repositories. Please read our guidelines for more information. <p><br><br>By submitting to GigaScience, you are aware of the journal's AI-writing tools policy, and if you have declared use of such tools below, you have acknowledged this where appropriate in your manuscript and have made a summary of use and outputs available. <p><br><b>AI-assisted writing tools have been used in the preparation of this manuscript?	No

Cover Letter Click here to access/download;Manuscript;scLR-CoverLetter.pdf

BGI Research

State Key Laboratory of Genome and Multi-omics Technologies
NO. 9, Xianyun Road
Shenzhen, China, 518085

华大生命科学研究院 BGI·Research

Apr. 20, 2026

Dear Editor,

We are pleased to submit our manuscript entitled "Single-cell Long-read Transcriptomics: From Technologies to Biological Insights" for your consideration in *Giga Science.*

Single-cell transcriptomics has significantly enhanced the resolution at which cellular heterogeneity can be characterized, uncovering the diverse gene expression landscapes within complex tissues. Capturing the full complexity of transcriptomes, however, has remained a major challenge. Single-cell long-read sequencing (scLR-seq) represents a breakthrough, providing the unprecedented ability to directly profile full-length transcripts at single-cell resolution. By revealing the landscape of isoform diversity and cell-type specific regulation, scLR-seq opens new avenues for deciphering complex biological processes, detecting rare molecular events, and driving advances in disease research and precision medicine. In this review, we highlight recent advances in scLR-seq, covering technological innovations, computational frameworks, and the biological insights they enable from isoform dynamics to allele-specific expression across diverse systems, while discussing future directions for integrating scLR-seq with spatial and multi-omics approaches to decode complex cellular programs.

Key highlights of our study include:

Technological Evolution: We provide a comprehensive comparison of leading long-read platforms, specifically Nanopore and PacBio SMRT sequencing, highlighting their unique mechanisms and signal processing strategies.

Analytical Frameworks: The article details end-to-end computational workflows: from raw data processing and demultiplexing to isoform-level quantification and transcript discovery.

Biological Insights: We emphasize the power of scLR-seq in resolving isoform usage dynamics, allele-specific expression, and its application in diverse fields such as immunology, neurodevelopment, and oncology.

Future Directions: We discuss the emerging potential of integrating scLR-seq with spatial transcriptomics and other multi-omics modalities to decode complex cellular programs.

As possible referees, we would suggest the following experts of single-cell omics and long-read sequencing:

Jue Ruan (ruanjue@caas.cn) from the Chinese Academy of Agricultural Sciences, for his expertise in long-read sequencing and assembly algorithm development.

Lan Jiang (jiangl@big.ac.cn) from the China National Center for Bioinformation, for his

expertise in single-cell genomics and transcriptomics.
Xi Chen (chenx9@sustech.edu.cn) from the Southern University of Science and Technology, for his expertise in single cell genomics techniques development.
Xiaoying Fan (fan_xiaoying@gzlab.ac.cn) from the Guangzhou National Laboratory, for her expertise in single cell long-read genomics.
Jixian Zhai (zhaijx@sustech.edu.cn) from the Southern University of Science and Technology, for his expertise in long-read sequencing technology development.

We believe that our review of scLR-seq will be of interest to your readership. This manuscript provides an overview of recent advances in the field, along with a discussion of current challenges and future directions. The manuscript is original, not under consideration elsewhere, and all authors have approved its submission. And we declare no conflicts of interest. Thank you for your time and consideration. We look forward to hearing from you soon.

Best regards,
Sincerely,

Chuanyu Liu, Ph.D.
State Key Laboratory of Genome and Multi-omics Technologies
BGI-Research, Shenzhen, China
Email: liuchuanyu@genomics.cn

Manuscript Click here to access/download;Manuscript;scLR_Manuscript.pdf

Single-cell Long-read Transcriptomics: From Technologies to Biological Insights

Ze-Hui Ren[1,2]†, Wenteng Liu[1,3]†, Jianhua Yin[1,4,5] & Chuanyu Liu[1,4,5]*

[1]State Key Laboratory of Genome and Multi-omics Technologies, BGI Research, Shenzhen 518083, China
[2]Shenzhen Key Laboratory of Single-Cell Omics, BGI Research, Shenzhen 518083, China.
[3]National Engineering Research Center for Nanomedicine, College of Life Science and Technology, Huazhong University of Science and Technology, Wuhan 430074, China
[4]Shenzhen Proof-of-Concept Center of Digital Cytopathology, BGI Research, Shenzhen 518083, China
[5]Shanxi Medical University-BGI Collaborative Center for Future Medicine, Shanxi Medical University, Taiyuan 030001, China
†Contributed equally to this work
*Corresponding author (Chuanyu Liu, email: liuchuanyu@genomics.cn)

Abstract

The rapid advancement of single-cell long-read transcriptomics (scLR-seq) is transforming transcriptomic research. Unlike traditional approaches, scLR-seq enables the direct capture of full-length transcript structures, isoform usage patterns, and precise information on alternative splicing and transcription start and end sites at single-cell resolution. These capabilities provide an unprecedented molecular view for dissecting cellular heterogeneity, developmental trajectories, and disease mechanisms. This review summarizes the technological evolution of scLR-seq and its potential to illuminate transcriptomic complexity, cellular states, and regulatory networks across key biological domains, including immunology, neurodevelopment, and oncology. We provide a comprehensive overview of the end-to-end analytical workflows, spanning from raw data processing to transcript discovery and quantification. We also discuss downstream biological analyses, with emphasis on cell-type annotation and the resolution of isoform usage dynamics. Finally, we highlight applications of scLR-seq in diverse biological systems, such as the immune system, neural development, and tumor microenvironments, and consider future opportunities and challenges in integrating multi-omics data to decode cellular programs and disease evolution.

Keywords

Single-cell long-read transcriptomics; Full-length isoform resolution; Third-generation sequencing; scLR-seq Analytical Workflows; Transcript structural complexity; Immune profiling

Background

Single-cell transcriptomics has significantly advanced our understanding of cellular heterogeneity, developmental trajectories, and disease-related state transitions over the past decade. At the heart of this progress lies single-cell RNA sequencing (scRNA-seq), which has become a cornerstone technology in biological and medical research [1-4]. Short-read scRNA-seq is widely used for cellular heterogeneity, identifying rare cell populations, and analyzing dynamic cell states, owing to its primary strengths: high throughput and quantitative stability [5-8]. However, short reads typically cover only a small portion of a transcript, imposing inherent limitations on resolving full-length transcript structures. Complex transcript structure features, such as alternative splicing, transcription start site (TSS) selection, and transcription end site (TES) choice, often require inference rather than direct observation when using short reads, and the detection of fusion transcripts and allele-specific expression is similarly constrained by the inability to link distant variants along the same molecule [9-12]. In many biological contexts, cell states are determined not just by whether a gene is expressed, but by which specific transcript isoforms are present. Capturing full-length transcript structures is therefore essential for understanding how a single gene can produce multiple functional proteins or regulatory RNAs within an individual cell [13-15].

The rise of long-read sequencing (LRS) offers a novel approach to addressing these challenges. Both Pacific Biosciences (PacBio), with its high-accuracy circular consensus sequencing (CCS), and Oxford Nanopore Technologies (ONT), through its real-time nanopore sequencing platforms, enable the detection of full-length RNA or cDNA at a single-molecule level. These platforms have effectively [16, 17]. These platforms have effectively overcome the fragmentation issues inherent in legacy sequencing technologies [18, 19]. Driven by improvements in sequencing accuracy, optimization of cell-barcoding strategies, and the rapid evolution of demultiplexing algorithms, single-cell long-read transcriptomics is transitioning from a proof-of-concept stage into a mature and standardized technological platform [13, 14, 20]. This evolution is further supported by the increasing availability of specialized bioinformatics pipelines tailored for long-read data [14]. Beyond simply increasing read length, the primary advantage of long-read technologies lies in their ability to simultaneously capture transcription start sites, internal splicing patterns, transcription end sites, and sequence variations within the same RNA molecule [9, 21, 22]. By providing a direct physical tether between these structural features, this technology enables the comprehensive profiling of full-length isoforms, bypassing the inherent ambiguity of transcript inference associated with fragmented short-reads. Consequently, this transition expands the scope of single-cell analysis from simple gene-level quantification to isoform-level regulation. The ability to resolve full-length transcript architectures now enables the exploration of functional diversity arising from alternative processing with unprecedented precision [15, 23-25].

As a result, single-cell long-read transcriptomics has rapidly expanded its application boundaries in recent years. On one hand, it offers definitive molecular evidence for investigating alternative splicing dynamics, isoform switching, alternative promoter usage, and alternative polyadenylation [23, 25-28]. By capturing the full connectivity of RNA molecules, these methods provide a much clearer picture of how diverse regulatory mechanisms coordinate within individual cells. On the other hand, this technology facilitates the systematic interrogation of transcriptomic features previously challenging to resolve at the single-cell level, including somatic mutations [29],

allele-specific expression [19], fusion transcripts, transposable element-derived sequences [30], and a vast array of unannotated isoforms [19, 31]. Integrating these multi-dimensional transcriptomic features into a unified analytical framework enables a more holistic understanding of cellular identity and function [13]. Furthermore, as long-read sequencing is increasingly integrated with short-read data, spatial transcriptomics [32-34], and other multi-omics modalities, it is no longer relegated to a supplementary role. Instead, it is redefining the scope of biological inquiry across an expanding range of experimental contexts. Ultimately, these advancements are propelling the field toward an unprecedentedly complete and high-resolution understanding of the transcriptome.

Against this backdrop, the present article systematically reviews the core technologies, pivotal computational pipelines, and diverse applications of single-cell long-read transcriptomics. Particular emphasis is placed on isoform-level characterization and the integration of transcriptional regulatory layers, offering a forward-looking perspective on their roles in decoding human health and disease.

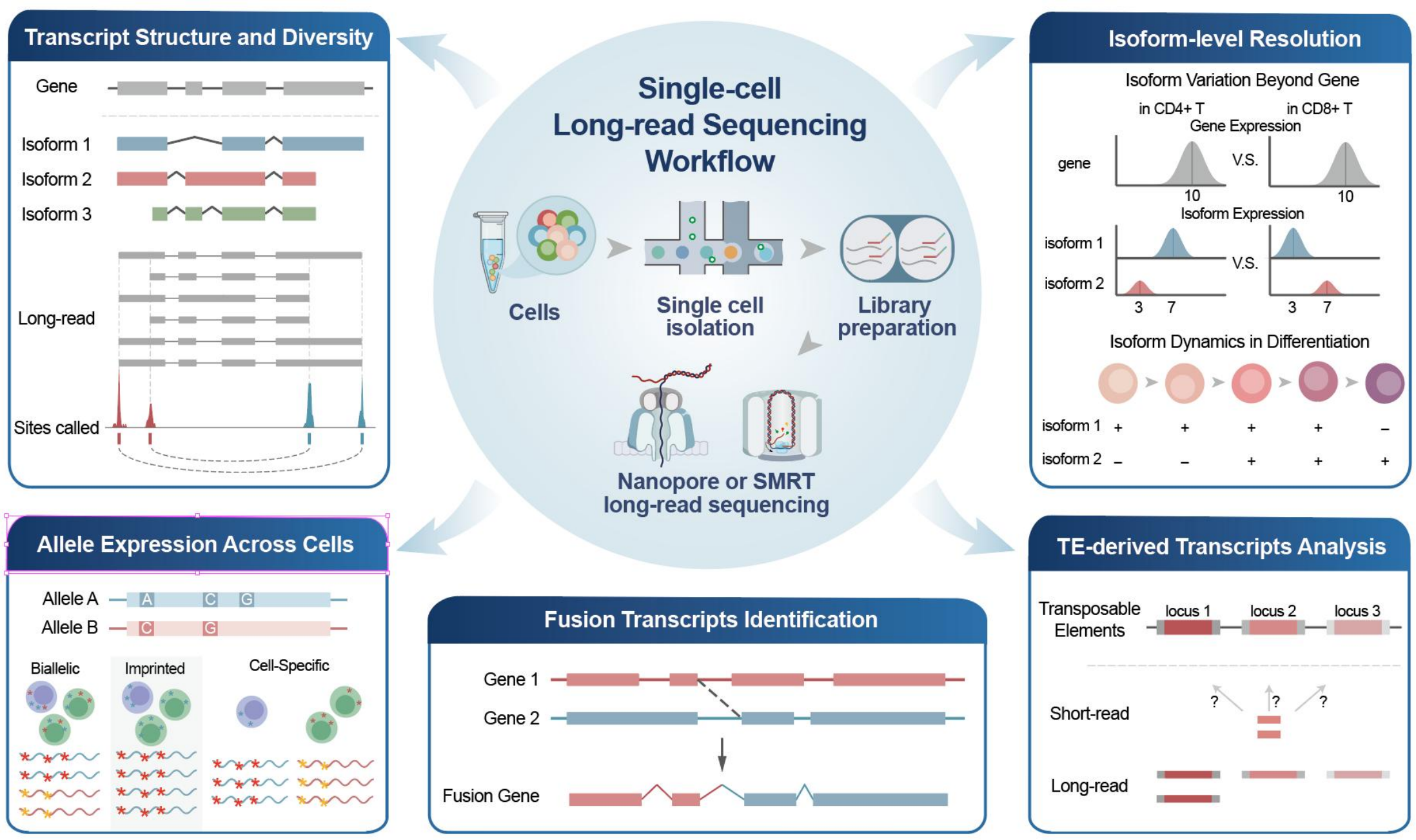

Figure 1. Overview of single-cell long-read RNA sequencing and its analytical applications. Single-cell long-read sequencing enables isolation of individual cells, library preparation, and sequencing on Nanopore or PacBio SMRT platforms. This approach captures full-length transcripts, allowing reconstruction of isoform structures, exon connectivity, and transcription start/end sites, as well as quantification of isoform diversity. It provides isoform-level resolution for assessing cell-type-specific expression and dynamic changes during differentiation. Full-length reads with cell barcodes and UMIs enable allele-specific expression analysis, revealing imprinting and allelic biases across cell populations. Fusion transcripts can be unambiguously detected, defining both fusion partners and their full-length structures. Long reads also facilitate analysis of transposable element-derived transcripts, resolving locus-specific expression and integration into novel isoforms. Together, these capabilities provide a comprehensive molecular view of transcriptomic complexity at single-cell resolution.

Single-Cell Long-Read Transcriptome Sequencing Technologies

Platforms for Long-read Sequencing

Current long-read sequencing technology has primarily converged into two distinct technical modalities: the Circular Consensus Sequencing platform, represented by Pacific Biosciences, and the nanopore-based electrical signal detection platform, led by Oxford Nanopore Technologies. In recent years, the LRS landscape has further diversified with the emergence of novel nanopore such as MGI's CycloneSEQ, Qitan tech's QNome and Polyseq Biotech's Polyseq. It is important to emphasize that the rapid development of this field stems not only from continuous improvements in hardware but also from breakthroughs in artificial intelligence and deep learning for signal processing and error modeling [35, 36]. These advancements have transformed LRS from a physical measurement technology into a data-driven analytical system.

Nanopore-based platforms: Nanopore decode sequences in real-time by monitoring ionic current fluctuations as nucleic acid molecules translocate through protein nanopores [37]. While iterative refinements in pore chemistry and basecalling algorithms have significantly enhanced accuracy, the error profile remains partially stochastic and inherently dependent on downstream computational modeling. Given that raw electrical signals are highly noisy and non-linear, deep learning architectures have become the indispensable backbone of basecalling [38, 39]. The basecalling frameworks have transitioned from early Recurrent Neural Networks (RNNs) to the Transformer-based architectures (e.g., Dorado [40]) predominant today, which excel at capturing long-range dependencies and improving accuracy in homopolymer regions [41]. Beyond basecalling, deep learning now facilitates signal denoising, epigenetic modification detection (e.g., 5mC, m6A) [42], and real-time adaptive sampling [43]. These advancements enable nanopore platforms to integrate ultra-long read acquisition with direct RNA sequencing and the concurrent mapping of chemical modifications. Furthermore, features such as device miniaturization, real-time data output, and adaptive sampling make nanopore platforms highly advantageous for rapid clinical diagnostics or field sequencing [37].

Circular Consensus Sequencing-Based Platforms: In contrast, PacBio's Single-Molecule Real-Time (SMRT) sequencing employs a distinct 'physical redundancy' strategy. By ligating hairpin adapters to create circular SMRTbell templates, the DNA polymerase can traverse the same molecule multiple times [44, 45]. The resulting subreads are computationally integrated into highly accurate HiFi reads, effectively neutralizing stochastic errors. This process has been further refined by DeepConsensus, a deep learning framework that models complex error patterns across subreads to enable non-linear error correction, even in low-coverage or homopolymer-rich regions [46]. While CCS read lengths are inherently shorter than the extreme lengths possible with nanopore sequencing, they nonetheless encompass the full extent of most eukaryotic transcripts. Moreover, upported by high-throughput platforms like Revio, this approach enables Q30–Q50 high-fidelity profiling for long-read sequencing [47]. Consequently, CCS highly effective for detecting single-nucleotide variants (SNVs), small indels, and supporting telomere-to-telomere (T2T) assemblies, making it a cornerstone for precision medicine and allele-specific expression analysis [18, 48]. However, higher per-base costs and stringent input quality requirements remain significant hurdles for broad adoption.

The selection of a long-read platform is increasingly a matter of strategic alignment with research goals. PacBio HiFi is the preferred choice for applications demanding base-level fidelity, such as somatic mutation calling, allele-specific expression (ASE), and precise fusion breakpoint mapping [9]. Conversely, nanopore platforms offer significant advantages in cost-efficiency and scalability, making them the preferred choice for large-scale cohorts and high-throughput sequencing [15, 18]. Beyond individual platform strengths, long-read sequencing is driving a paradigm shift in transcriptomics, moving from traditional gene-level quantification toward full-length transcript isoform resolution. This transition enables the simultaneous characterization of TSS/TES variation and complex splicing events at single-cell resolution, allowing multiple regulatory layers to be analyzed within the same molecular context.

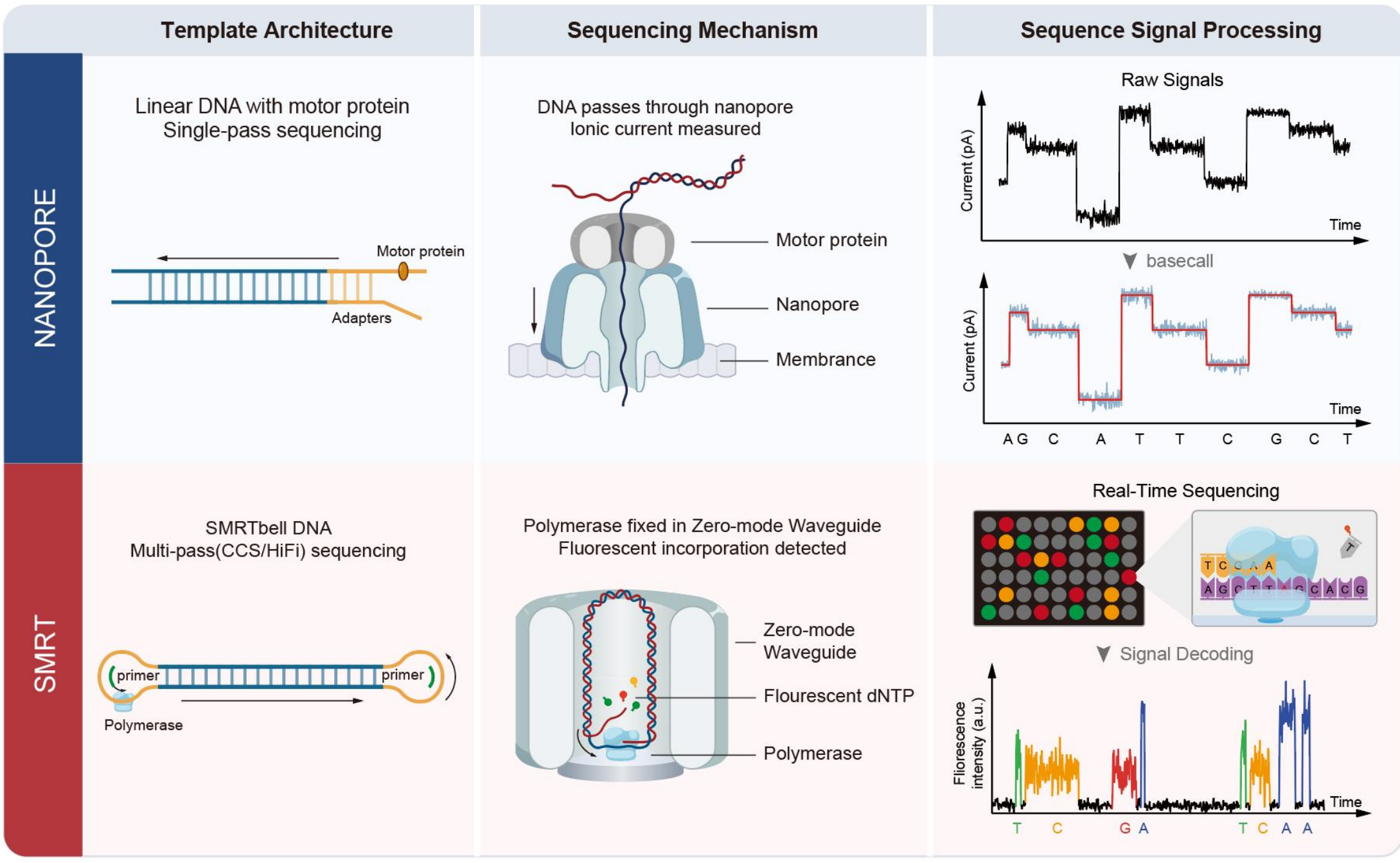

Figure 2. Comparison of Nanopore and PacBio SMRT long-read sequencing platforms. The figure illustrates the core differences in template architecture, sequencing mechanism, and signal decoding for the two major long-read technologies. Template Architecture: Nanopore sequencing uses linear DNA or RNA molecules with attached motor proteins and adapters, allowing single-pass sequencing of individual molecules. In contrast, SMRT sequencing relies on circular SMRTbell templates, where polymerases repeatedly replicate the same DNA molecule to generate multiple subreads for high-accuracy consensus sequences (HiFi reads). Sequencing Mechanism: In Nanopore sequencing, nucleic acids pass through a protein nanopore embedded in a membrane, and changes in ionic current caused by each nucleotide are measured in real time. SMRT sequencing uses DNA polymerase immobilized in ZMWs to incorporate fluorescently labeled nucleotides. Fluorescence signals from each incorporation event are captured to infer the nucleotide sequence. Signal Decoding: Raw current signals from Nanopore sequencing are processed by basecalling algorithms to translate ionic current changes into nucleotide sequences. SMRT sequencing signals are decoded from fluorescence traces, with real-time analysis of nucleotide incorporation generating high-fidelity consensus reads. These differences illustrate how the two platforms balance read length, sequencing accuracy, and real-time detection capabilities.

scLR-seq Library Construction Strategies

Core Library Preparation Strategies: During the development of single-cell long-read sequencing, a pivotal challenge has been the high-fidelity recovery and assignment of cell barcodes (CBs) and unique molecular identifiers (UMIs). Over the past few years, multiple library preparation strategies have been developed. These methodologies are primarily distinguished by their reliance on orthogonal short-read sequencing to facilitate accurate CB and UMI identification, or their ability to perform de novo barcode deconvolution directly from long-read data.

Table 1. Core library construction strategies

Method	Concept	Advantages	Limitations
ScNaUmi-seq [31]	Split-library: short-read barcodes guide nanopore full-length reads	Accurate molecule assignment; concordant with short-read data	Requires paired short-read sequencing and dual-platform workflow
ScISOr-Seq [49]	PacBio full-length sequencing of barcoded cDNA from droplet-based scRNA	High HiFi accuracy; enables cell-type-specific isoform discovery	Lower throughput; needs dedicated processing
scCOLOR-seq [50]	Bi-nucleotide barcodes enable error detection and direct ONT correction	Short-read-free; supports isoform, fusion and transcript quantification	Requires custom barcoded beads and prior barcode knowledge
MAS-seq [51]	Concatenates multiple cDNAs into long molecules to boost PacBio throughput	Greatly increases throughput; integrates with 10x workflows	Needs extra concatemer construction; may limit very long transcripts
Anchor-Enhanced Bead Design [52]	Anchor-guided barcode/UMI design to reduce truncation errors	Improves UMI recovery and transcript detection at library design level	Requires custom bead design; mainly tackles synthesis rather than sequencing errors
scLIS-seq [53]	Plate-based long-read isoform sequencing using Smart-seq3xpress-derived amplification	High sensitivity for low-abundance isoforms and complex transcript detection	Low throughput; dependent on FACS/manual cell isolation; ONT errors need correction
BLAZE [54]	Adapter/poly(T)-guided barcode and UMI recovery with quality filtering	Removes short-read dependence; high barcode recovery accuracy	Needs predefined barcode structure and expected cell counts; high-sensitivity mode increases false positives
Sockeye	Official ONT demultiplexing workflow for barcode/UMI recovery from 10x libraries	User-friendly, automated and integrated into EPI2ME environment	Relies on 10x whitelist; may yield higher false positives for low-quality reads
scNanoGPS [26]	De novo barcode calling via iCARLO; optional variant analysis	Short-read and whitelist-free; supports joint genotype sphenotype profiling	Computationally intensive; parameter tuning required; ONT error correction still needed
R2C2 [55]	Rolling-circle amplification with consensus sequencing for ONT error correction	Improves accuracy through consensus; cost-effective full-length profiling	May introduce amplification bias; throughput tied to circle generation efficiency
Flexiplex [56]	Demultiplexing by Levenshtein distance with whitelist or de novo barcode discovery	Supports custom barcode designs; error-tolerant and flexible	Requires user-specified barcode structure; less straightforward configuration
FLT-Seq [57]	Split-library 10x sequencing with short-read-guided barcode assignment and clustering	Low-abundance isoform detection with full-cell coverage	Still requires dual-platform sequencing and split library

LR-Split-seq [58]	Short-read-guided cell-state definition with long-read sequencing of selected cells	Reduces cost by sequencing a subset of cells; suitable for following up existing atlases	Long reads cover only subset of cells; rare isoforms may be missed
SCAN-seq [59]	RT primer integrates cell barcodes for pooled nanopore sequencing of full-length cDNA	Enables alternative splicing, novel transcript and allele-specific analyses	Limited throughput and labor intensive; ONT errors still need correction
SCAN-seq2 [60]	Combinatorial different 3′ and 5′ barcoding for scLR	Improves throughput and supports immune-receptor analysis	Complex library preparation and demultiplexing
scNanoCOOL-seq[61]	Multi-omics profiling of transcriptome, DNA methylation, chromatin and CNVs	Integrates transcriptomic and epigenomic information in the same cell	High cost; limited per-cell coverage
RAGE-Seq [62]	Targeted long-read immune receptor sequencing linked to droplet-based scRNA-seq	High-throughput clonotype-resolved profiling with matched transcriptomes	Focuses on immune receptors; not transcriptome-wide

Early mainstream approaches relied on short-read-assisted strategies. Representative methods, such as ScISOr-Seq [49], ScNaUmi-seq [31], and FLT-Seq [57], adopt a parallel library design. In these workflows, full-length cDNA molecules generated using platforms like 10x Genomics are aliquoted into two fractions: one is sequenced using short-read platforms (e.g., Illumina) to generate a high-fidelity CB/UMI 'dictionary' and cell clusters, and the other using long-read platforms (PacBio or ONT) to capture full-length transcript architectures. By convoluting both datasets through shared barcodes, these approaches achieve accurate cell identity assignment alongside comprehensive isoform resolution. While this dual-platform strategy offered high reliability during the early stages of LRS, it is often hampered by operational complexity, increased costs, and the need for sophisticated bioinformatic integration.

An alternative short-read-dependent approach is exemplified by methods such as LR-Split-seq [58] and RAGE-seq [62]. Instead of parallel sequencing, these methods first utilize short-read data for initial cell clustering and then selectively apply long-read sequencing to specific cell populations of interest. This targeted enrichment significantly reduces sequencing costs and enhances efficiency, though it may result in a sampling bias that misses rare cell types or cryptic isoforms. Such strategies have proven particularly powerful in immunogenomics; for instance, RAGE-seq enables the simultaneous reconstruction of full-length TCR/BCR repertoires and their corresponding cellular identities [62].

More recently, improvements in long-read accuracy and computational methods have enabled the development of short-read-free strategies. These approaches aim to directly demultiplex CBs and UMIs from long-read data, thereby simplifying workflows and reducing cost. Their key innovations lie in both barcode design and computational algorithms.

At the molecular level, strategies like scCOLOR-seq [50] employ redesigned barcode and UMI system based on dimer nucleotide blocks, embedding error-detection capability at each position. This allows direct error correction even in the presence of nanopore sequencing errors. Similarly, anchor-enhanced bead designs [52] incorporate defined anchor sequences between barcode and UMI regions, improving boundary identification and reducing synthesis-related errors. In addition, some approaches improve accuracy through molecular strategies. For example, R2C2

[55] generates concatemeric reads via rolling circle amplification to produce high-confidence consensus sequences. scLIS-seq [53] adopts a plate-based strategy combined with Smart-seq3xpress, processing each cell individually. Although limited in throughput, it offers high sensitivity for rare cell populations.

At the computational level, sophisticated demultiplexing tools have bridged the accuracy gap. BLAZE [54] uses a three-step strategy: barcode localization, quality filtering, and frequency-based selection, and it achieves high concordance with short-read benchmarks using whitelist correction [14]. In contrast, scNanoGPS [26] employs a de novo clustering-based algorithm (iCARLO) that does not rely on predefined barcodes and can simultaneously integrate genotype–phenotype analysis. For users with non-standard library designs, Flexiplex [56] provides an error-tolerant matching framework that supports arbitrary barcode configurations. ONT's wf-single-cell pipeline integrates the Sockeye demultiplexer with downstream analysis, providing an automated and user-friendly workflow.

According to recent benchmarking studies [63], short-read-free approaches are rapidly approaching the accuracy of short-read-assisted methods, while offering advantages in cost, scalability, and experimental simplicity.

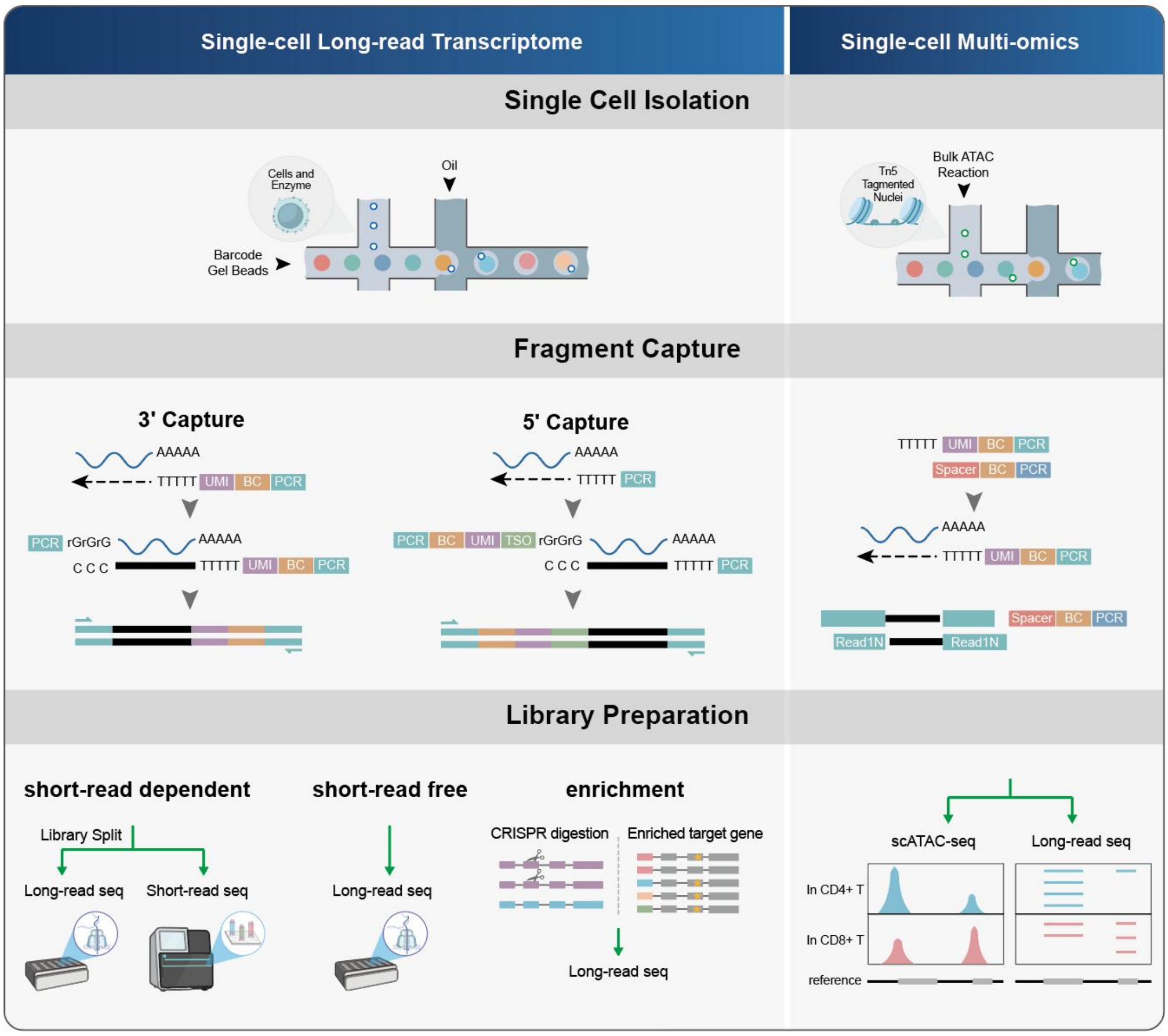

Figure 3. Overview of single-cell long-read transcriptome workflows and potential for multi-omics integration. Individual cells are first isolated using microfluidic or droplet-based

platforms, with barcoded beads introducing cell barcodes and UMIs to enable single-cell resolution. Transcript fragments can be captured via 3' or 5' end enrichment, linking reads to specific cells and molecules. Long-read library preparation can follow short-read-dependent workflows, where cDNA is split for parallel Illumina and long-read sequencing, or short-read-free workflows that recover barcodes and UMIs directly from long reads using error-correction strategies. Specialized enrichment, such as CRISPR-mediated target capture, allows profiling of low-abundance or specific transcripts. In addition to standalone transcriptome analysis, these workflows can be integrated with other single-cell modalities, such as chromatin accessibility or targeted epigenetic profiling, enabling multi-omics characterization of cellular states, isoform diversity, and regulatory programs.

Specialized and Application-Oriented Library Designs: Specialized and application-oriented library designs have significantly expanded the utility of single-cell long-read sequencing (scLR-seq). By integrating targeted enrichment, molecular depletion, or multi-omics coupling into standard workflows, these bespoke strategies enable researchers to address specific biological questions

For low-abundance transcript detection, CRISPR-based depletion strategies, such as scCLEAN [64], selectively remove highly abundant non-target transcripts using CRISPR-Cas9 system prior to library construction. This approach effectively reallocates sequencing capacity toward low-abundance transcripts and rare isoforms. Conversely, targeted long-read methods, such as scRaCH-seq [65], use biotinylated probes to capture transcripts of interest, followed by streptavidin-based enrichment. This strategy can also be applied retrospectively to existing 10x cDNA libraries, enabling targeted long-read sequencing without additional sample preparation. In immune receptor studies, RAGE-seq [62] is specifically designed to capture full-length T cell receptor (TCR) and B cell receptor (BCR) sequences. It preserves complete V(D)J recombination information and can be integrated with short-read-based cell atlases to link immune repertoire features with cellular identity. In multi-omics integration, methods such as SPLONGGET [66] and ScISOr-ATAC [67] enable the simultaneous profiling of full-length transcriptomes and chromatin accessibility within the same cell. These approaches provide concurrent measurements of gene expression, isoform diversity, and epigenetic regulation, offering a powerful framework for studying cis-regulatory mechanisms. Addressing the complexity of repetitive elements, CELLO-seq [30] optimizes the detection of transposable elements (TEs) by incorporating extended UMIs (~50 nt) and RYN-based error-correction. This enables locus-specific resolution, effectively distinguishing autonomous TE expression from passive readthrough events. Finally, SMART-Seq-Total [68] provides a truly comprehensive view by utilizing E. coli poly(A) polymerase to tail all RNA species, including non-polyadenylated species such as miRNAs, snoRNAs, and histone RNAs. This strategy facilitates template-switching-mediated cDNA synthesis for the entire transcriptome, making it one of the few platforms capable of capturing both coding and non-coding RNA diversity at single-cell resolution

Table 2. Specialized strategies for single-cell long-read library preparation

Method	Library	Key Features	Outputs	Limitations
SMART-Seq-Total[68]	Plate-based full-length total-RNA cDNA library (96/384-well; FACS-sorted cells)	Full-length total-RNA capture with UMI; broad RNA biotype coverage	mRNA, lncRNA, miRNA, snoRNA, snRNA, tRNA, histone RNA, and related RNA biotypes	rRNA depletion is often required; limited throughput; complex workflow
scRaCH-seq[65]	10x Chromium cDNA library (3′/5′ compatible)	Probe-based targeted long-read resequencing from 10x cDNA	Full-length targeted isoforms, point mutations, and splicing variants	Restricted to targeted genes; requires probe-panel design; enrichment may introduce bias
scCLEAN[64]	10x Chromium 3′ cDNA library with CRISPR-mediated depletion	CRISPR depletion of abundant transcripts; boosts rare isoform detection	Low-abundance transcripts, rare isoforms, difficult-to-detect transcript classes	Extra CRISPR step required
CRISPR-edited Single-cell profiling[69]	Plate-based single-cell cDNA library after CRISPR perturbation	Single-cell long-read readout of CRISPR-induced isoform changes	Edited loci, transcript-structure consequences, and full-length edited isoforms	Target-focused; requires customized perturbation design and dedicated analysis
CELLO-seq[30]	Plate-based full-length cDNA library (FACS-sorted cells)	Long-UMI ONT profiling for locus-specific TE transcripts	TE locus-specific expression, TE-chimeric transcripts, and TE subfamily activity	Limited throughput; complex UMI design
ScISOr-ATAC[67]	Single-nucleus split RNA + ATAC libraries	Same-nucleus profiling of full-length transcripts/splicing and chromatin accessibility	Chromatin accessibility, full-length transcripts, splicing, and chromatin–splicing coupling	Complex dual-omics workflow
ouro-seq[70]	10x Chromium full-length mRNA cDNA library	Full-length single-cell mRNA profiling for splicing and APA	Full-length mRNA isoforms, alternative splicing, alternative promoter usage, and APA	Complex experimental and computational workflow
SPLONGGET[66]	10x Genomics Single Cell Multiome ATAC + Gene Expression library	Same-cell/nucleus profiling of genome, open chromatin, and full-length transcriptome with optional targeted enrichment	Small variants, structural variants, CNAs, chromatin accessibility, full-length transcripts, and splice-affecting variants	Experimental design, sequencing cost, and downstream integrative analysis are all relatively demanding
scGTP-seq[71]	Single-cell genome + transcriptome co-assay library	Single-cell multi-omics framework linking structural genomic variation to transcriptomic profiles	ecDNA, structural variants, and associated transcriptomic changes	Genotype–phenotype multi-omics workflow
FlsnRNA-seq[72]	Protoplasting-free single-nucleus cDNA library	Protoplasting-free full-length single-nucleus RNA profiling in plants using isolated nuclei	Nuclear full-length transcripts and isoforms in plants	Plant-specific and nucleus-focused
m6A-isoSC-seq[73]	10x Chromium cDNA library with APOBEC1-YTH labeling	Single-cell full-length isoform profiling with m6A resolution	m6A at isoform and single-cell levels, misprocessed isoforms, and modification associated transcript states	Requires matched short-read sequencing and an engineered DART/APOBEC1-YTH system; specialized workflow

Computational Methods for Data Processing

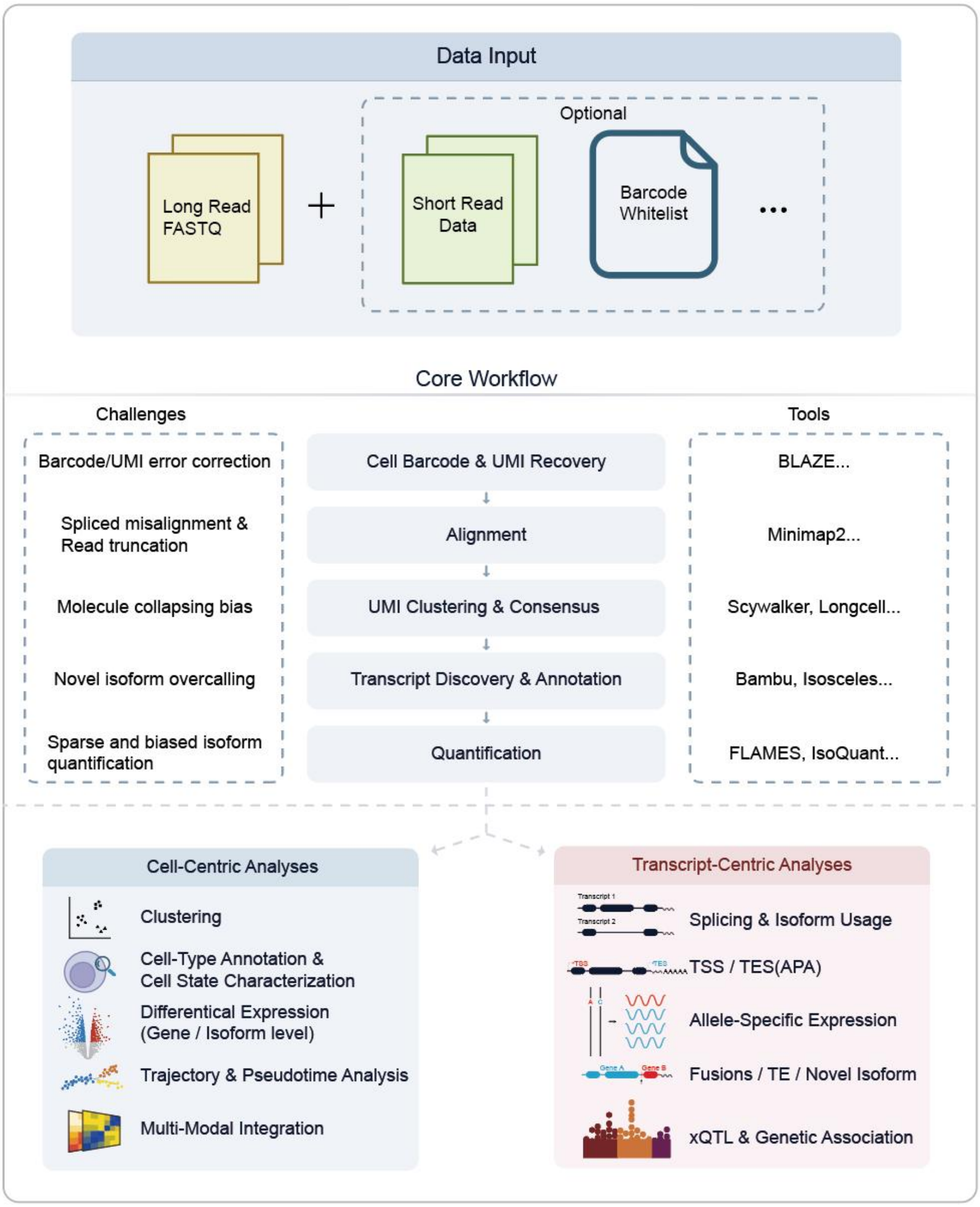

Figure 4. Overview of single-cell long-read RNA sequencing analytical workflow. The core workflow addresses common challenges in single-cell long-read transcriptomics, including barcode/UMI errors, spliced misalignment, read truncation, molecule collapsing bias, novel isoform overcalling, and sparse or biased isoform quantification. Key computational steps include cell barcode and UMI recovery, read alignment, UMI clustering and consensus building, transcript discovery and annotation, and isoform-level quantification. Downstream analyses are divided into cell-centric and transcript-centric approaches. Cell-centric analyses include clustering, cell-type annotation and characterization, differential expression, trajectory and pseudotime inference, and multi-modal data integration. Transcript-centric analyses focus on splicing and isoform usage, transcription start/end site (TSS/TES) analysis, allele-specific expression, detection of fusion or

novel transcripts, and integration with quantitative trait loci (QTL) and genetic association studies.

Cell Barcode and UMI Demultiplexing

In single-cell long-read sequencing (scLR-seq), accurate demultiplexing of CBsand UMIs is a prerequisite for achieving single-cell resolved quantification. However, long-read data, particularly from Nanopore platforms, are characterized by relatively high base-level error rates. This makes it challenging to reliably identify and correct short sequences, such as CBs and UMIs (typically 10–20 bp), embedded within reads. As a result, CB and UMI recovery has become a critical problem in computational method development. Based on their requirement for complementary sequencing data, current methodologies are broadly categorized into short-read-assisted and short-read-free approaches.

The early standard for scLR-seq, exemplified by the SiCeLoRe [31] pipeline, relies on high-accuracy short-read sequencing (e.g., Illumina) to provide a CB and UMI reference that guides the demultiplexing of long reads. In these workflows, the same 10x cDNA library is sequenced using both short- and long-read platforms. The short-read data provide a high-confidence reference set of CB–UMI combinations, which are then used to assign long-read sequences at the single-cell level. The primary advantage of this strategy is its exceptional accuracy, as the short-read "whitelist" provides a reliable anchor for correcting long-read sequencing errors. However, this approach significantly increases experimental complexity, time, and sequencing costs. Furthermore, integrating multi-platform datasets imposes a higher burden on bioinformatics processing [14, 15].

With improvements in sequencing accuracy and algorithm design, short-read-free approaches have become increasingly dominant. These methods aim to recover CB and UMI information directly from long-read data without external references. They can be further divided into whitelist-based and de novo strategies.

Whitelist-based methods rely on predefined barcode reference sets, typically provided by platforms such as 10x Genomics. Tools such as BLAZE [54] adopt a multi-step strategy: identifying candidate CB and UMI regions based on adapter and poly(T) signals, applying quality filtering, and selecting true barcodes based on frequency thresholds before mapping them to the whitelist. Similarly, ONT's wf-single-cell pipeline [74] integrates the Sockeye demultiplexer, which uses model-based inference combined with whitelist correction. The PacBio scIso-Seq workflow employs locality-sensitive hashing (LSH) to accelerate barcode matching, enabling efficient mapping of candidate sequences to the closest whitelist entries. These approaches are generally accurate and computationally efficient, particularly for standard 10x-based protocols, but their applicability is limited when barcode designs deviate from predefined schemas.

In contrast, de novo methods offer greater flexibility by inferring barcodes directly from the long-read data. For example, scNanoGPS [26] employs the iCARLO algorithm to cluster similar barcode sequences and identify valid CBs based on abundance patterns, without requiring any prior whitelist. This allows it to accommodate both standard and custom barcode designs. However, such approaches are typically more computationally intensive and sensitive to parameter settings, which may affect robustness and reproducibility.

Overall, continued advances in demultiplexing algorithms have significantly improved the recovery of valid molecules from long-read data, thereby enhancing the reliability of scLR-seq analyses. Furthermore, integrative frameworks such as Scywalker [75] are emerging to unify multiple barcode identification strategies within a modular and extensible architecture. By supporting cross-platform data processing and flexible method selection, these frameworks highlight a transition from standalone tools to a more standardized and interoperable analytical ecosystem, which will be critical for the broader adoption of single-cell long-read technologies.

Table 3. Cell Barcode and UMI Demultiplexing Methods

Method	Short-read-free	Whitelist needed	Primary task	Corrected element(s)	Correction strategy	Key feature
BLAZE [54]	✓	Yes	CB calling; UMI extraction	CB + UMI	Adapter/poly(T) localization + whitelist filtering + count cutoff	Count/quality-based 10x barcode calling from ONT reads
wf-single-cell [74]	✓	Yes	CB/UMI demultiplexing; count matrix generation	CB + UMI	Sockeye-based barcode calling + whitelist-guided correction	Official ONT Nextflow workflow built around Sockeye
scNanoGPS [26]	✓	No	CB calling; read assignment; optional variant analysis	CB + UMI	De novo CB calling + locus-aware UMI collapsing	iCARLO-based de novo cell calling without short reads or whitelist
scIso-Seq	✓	Yes	Barcode calling; UMI deduplication	CB + UMI	LSH-assisted whitelist rescue + edit-distance correction + groupdedup	Official PacBio HiFi single-cell workflow
Scywalker [75]	✓	Optional	End-to-end scLR workflow	CB + UMI	Read-count ranking + 1-mismatch merging	One-command workflow from FASTQ to gene/isoform counts
SiCeLoRe [31]	×	Yes	CB/UMI assignment; molecule consensus	CB + UMI	Short-read truth-set assignment + UMI-guided consensus	Short-read-guided CB/UMI assignment with molecule consensus
scTagger [76]	×	Yes	LR–SR barcode matching	CB	Trie-based approximate matching to short-read CBs	Fast approximate matching between long- and short-read CBs
Flexiplex [56]	✓	Optional	CB/UMI extraction; demultiplexing; barcode discovery	CB + UMI	Flanking-sequence search + Levenshtein matching / discovery mode	Levenshtein-based flexible demultiplexing
Longcell [34]	✓	Yes	CB/UMI recovery; isoform quantification	CB + UMI	k-mer barcode alignment + edit-distance filtering + UMI denoising	UMI-aware denoising for isoform quantification
ScNapBar [77]	×	Yes	Barcode assignment	CB	UMI matching or Naïve Bayes with Illumina-derived priors	Hybrid UMI/Bayesian barcode assignment
FLAMES [57]	✓	Optional	CB/UMI assignment; isoform quantification	CB + UMI	External CB calling + edit-distance UMI collapse	End-to-end isoform workflow; supports external CB callers
Scnanoseq [78]	✓	Yes	Barcode correction; UMI deduplication; quantification	CB + UMI	BLAZE-based calling + barcode correction + deduplication	Reproducible nf-core end-to-end ONT scRNA-seq pipeline

Transcript Discovery and Quantification

Following accurate assignment of CBs and UMIs, transcript discovery and quantification represent the key analytical steps for resolving isoform diversity and cell-type-specific splicing patterns in scLR. However, the combination of high data sparsity, ambiguity in splice junction detection, and incomplete transcript coverage poses substantial challenges for reconstructing full-length transcripts and obtaining robust expression estimates from individual cells.

From the perspective of transcript discovery, current approaches can be broadly classified into annotation-guided and de novo strategies. Traditional tools such as StringTie2 [79], while efficient for bulk data, show limited accuracy in single-cell settings. In contrast, Bambu and its updated version bambu-clump incorporate statistical modeling to distinguish true splice junctions from sequencing noise, enabling both accurate transcript reconstruction and reliable detection of novel isoforms [80, 81]. IsoQuant [82] adopts a splice graph-based framework to balance sensitivity and precision, and is particularly effective for resolving complex alternative splicing events. For PacBio data, the Iso-Seq pipeline (including scIso-Seq) remains a widely adopted standard, generating high-confidence full-length consensus transcripts through iterative clustering and polishing of CCS reads [83]. The ability of these tools to resolve the complete structure of an RNA molecule significantly reduces the ambiguity that previously hindered transcriptomic studies.

The evolution of quantification algorithms has also improved the accuracy of transcript-level expression matrices. Because individual long reads may ambiguously map to multiple transcript isoforms, expectation–maximization (EM) have become the dominant framework for resolving multi-mapping reads. Isosceles [84] is a leading method in this area, combining transcript compatibility counts (TCC) with EM optimization to achieve high quantification accuracy. Study shows that Isosceles outperforms earlier methods such as ESPRESSO [85], largely due to its improved handling of ambiguous read assignments. In addition, to address the limited sequencing depth of single cells, recent methods such as LRAA [86] introduce cluster-guided strategies. By pooling information from cells with similar biological profiles, these methods can recover low-abundance transcripts that might otherwise be lost in the noise of a single cell. For poorly annotated genomes or non-model organisms, Mandalorion [87] provides strong de novo reconstruction capabilities.

In practical research, end-to-end pipelines are increasingly adopted to streamline analysis workflows. Tools such as FLAMES [57] integrate barcode correction, transcript assembly (via Bambu [80]), and rapid quantification into a unified framework, enabling efficient processing from raw reads to expression matrices. Other frameworks, including TALON [88] and FLAIR2 [89], emphasize stringent filtering and validation of transcript models, and are commonly used in large-scale consortium projects to ensure reproducibility.

Collectively, these tools provide a range of complementary strategies, supporting complex splicing analysis and high-precision quantification. As these computational frameworks mature, they provide a solid foundation for exploring the full-length transcriptome at single-cell resolution with unprecedented resolution.

Table 4. Transcript Discovery and Quantification Methods

Method	Reference-guided novel transcript discovery	Annotation-free de novo discovery	Single-cell compatibility	Core principle	Strengths / recommended use
Bambu / banbu-clump [80, 81]	Yes	Limited	Native via clump	ML-guided reference-based discovery and context-aware quantification	Conservative, high-confidence discovery in annotated or shallow sc/spatial data
IsoQuant [82]	Yes	Yes	Adaptable to barcode-grouped single-cell data	Intron-graph reconstruction and annotation-assisted correction	Balanced sensitivity/precision; suitable for ONT and PacBio
Isosceles [84]	Yes	Yes	Native	DAG splice graph and TCC/EM quantification	Sensitive detection and accurate quantification, especially for sparse data
FLAMES [57]	Yes	Limited	Native	Barcode-aware semi-supervised isoform calling	Practical ONT workflow for isoforms, DTU, and mutation-aware analysis
FLAIR / FLAIR2 [89, 90]	Yes	Yes	Compatible after per-cell or grouped preprocessing	Alignment correction and isoform collapse; variant-aware in FLAIR2	High-confidence isoform filtering and variant-/haplotype-aware analysis
LRAA [86]	Yes	Yes	Native	Strand-specific splice-graph assembly and EM quantification	Flexible exploratory analysis across bulk, sc, and snRNA data
Mandalorion [87]	Optional	Yes	Native	Consensus-based isoform calling from accurate reads	Strong for poorly annotated or non-model systems
SCOTCH [87]	Yes	Yes	Native	Sub-exon modeling and novel-read clustering	Strong for isoform usage and DTU in barcoded sc long-read data
Iso-Seq [83]	Optional	Yes	Native	HiFi sprocessing, clustering/deduplication and isoform classification	Standard PacBio workflow for full-length isoform discovery
StringTie2 [79]	Yes	Yes	After cell/group preprocessing	Network-flow transcript assembly	Fast general-purpose baseline for annotated genomes
ESPRESSO [85]	Yes	Yes	After barcode-aware preprocessing	Error-aware splice-graph reconstruction and abundance estimation	Useful when splice correction and quantification are both priorities
TALON [88]	Yes	Limited	Preprocessing required	Database-based transcript tracking	Conservative multi-sample reproducibility tracking
Oarfish [91]	No	No	Backend for barcode-aware data	Probabilistic long-read quantification	Fast, memory-efficient complement to discovery tools
lr-kallisto [92]	No	No	After barcode recovery	Long-read pseudoalignment and EM quantification	Fast, low-memory quantification for large datasets
LongcellPre [34]	No	No	Native	CB/UMI recovery and consensus correction	Improves ONT quantification for downstream Longcell analysis
scNanoGPS [26]	No	No	Native	iCARLO deconvolution and consensus BAM generation	Best for joint isoform–mutation and genotype–phenotype analysis

Cell Type Annotation

Cell type annotation is a prerequisite for downstream biological interpretation, aiming to assign each cell a defined identity based on its transcriptomic profile. Existing approaches can be broadly categorized into three groups: marker gene-based methods (e.g., SCINA [93], scCATCH [94]), reference-based mapping approaches (e.g., SingleR [95], clustifyr [96]), and machine learning-based classifiers. These strategies have been extensively validated in short-read single-cell RNA sequencing and together form a well-established analytical framework.

However, a fundamental limitation arises when these methods are applied to long-read data. Nearly all current annotation frameworks operate at the gene-expression level and are inherently designed for short-read technologies. In contrast, single-cell long-read transcriptomics provides isoform-level resolution, capturing structural features such as alternative splicing and variation in transcription start and end sites. These features can carry cell-type-specific information that is not reflected at the gene level [22, 49, 97].

As a result, relying solely on gene-level expression may obscure functionally relevant heterogeneity encoded at the isoform level, thereby limiting the analytical value of long-read data. Despite this potential, dedicated frameworks for cell type annotation or clustering that directly leverage isoform-level information remain largely underdeveloped. In particular, robust computational methods that integrate isoform usage patterns into cell identity inference are still lacking, representing a critical gap in the current analytical ecosystem.

Future developments are likely to move toward multi-layered annotation strategies. Such frameworks would combine gene-level expression for coarse classification with isoform-level features for refined cell-type or state discrimination. Advances in deep learning models, particularly in representation learning for transcript features, are expected to facilitate this transition, enabling isoform-based cell type identification to evolve from a conceptual possibility into a practical analytical paradigm.

Biological Insights Enabled by Long Reads

Table 5. Functional Analysis Tools for Single-cell Long-read Transcriptomics

Method	Compatibility with SCLR	Analysis module	Key strength	Example application
IsoformSwitch AnalyzeR [98]	Compatible	Isoform switching and functional consequence analysis	Functional consequence analysis of isoform switches in long-read and single-cell data	Disease-associated isoform switching analyses
Longcell [34]	Native	Isoform usage and alternative splicing analysis	UMI-aware isoform quantification and splicing heterogeneity, spatial-ready	Spatial isoform switching; splice-factor perturbation targets
scCyclone [99]	Native	Differential isoform usage, differential splicing, and RBP analysis	Integrated DTU/DSE/RBP toolkit with rMAPS-compatible output	Public sc long-read demo datasets
Chevreul [100]	Compatible	Full-length single-cell transcript exploration and visualization	Exploratory analysis and visualization of full-length single-cell sequencing data	Full-length single-cell isoform and exon-coverage exploration

IsoTools 2.0 [101]	Compatible	Transcript structure, TSS, and alternative splicing analysis	Comprehensive TSS prediction, entropy, and splicing analysis	Long-read TSS prediction and AS analyses
scTSS [102]	Compatible	Transcription start site usage analysis	Alternative TSS detection and comparison from 5′ scRNA-seq	Cell-type-specific TSS and promoter-usage analysis
scAPA [103]	No	Alternative polyadenylation analysis	APA site usage analysis from 3′-tag scRNA-seq	Cell-type APA dynamics and PAU shifts
LongFUSE [104]	Native	Fusion transcript and fusion-isoform detection	Cell-level fusion and fusion-isoform calling with XOR-based candidate search	TMPRSS2–ERG fusion and fusion-positive tumor-cell identification
CTAT-LR-Fusion [105]	Native	Fusion transcript detection	Accurate long-read fusion calling with or without short-reads	Fusion detection in bulk and single-cell cancer transcriptomes
LongSom [29]	Native	Somatic variant, CNA, and fusion profiling	Joint calling of somatic SNVs, CNAs, and fusions from scLR	Ovarian-cancer clonal heterogeneity and prognostic subclones
ASPEN [106]	Compatible	Allele-specific expression analysis	Adaptive-shrinkage ASE modeling with improved sensitivity and dispersion control	F1 mouse brain organoids and T cells
scTE [107]	Compatible	Transposable element expression analysis	Family-level TE quantification for scRNA-seq	Cell-fate-specific TE dynamics in development and disease
SoloTE [108]	Compatible	Locus-specific transposable element expression analysis	Locus-aware TE quantification for scRNA-seq	Two-cell embryo and disease TE activity analyses
CELLO-seq [109]	Native	Locus-specific transposable element and TE-chimeric transcript analysis	Long-UMI framework for locus-specific young-TE and TE-chimeric transcript detection	Active versus readthrough TE transcription in single cells

Isoform Usage and Alternative Splicing Dynamics

In single-cell transcriptomics, defining cell types is only an initial step, whereas the deeper biological objective is to resolve the regulatory mechanisms that define cellular states. In this context, transcriptional regulation extends beyond gene-level expression and increasingly involves isoform usage and alternative splicing, which add an additional layer of functional diversity across cells. However, short-read sequencing was limited by its length, forcing researchers to infer alternative splicing indirectly through fragmented splice junction signals. The advent of LRS has enabled a shift from indirect inference to direct characterization of full-length isoforms, allowing splicing patterns to be resolved at the single-molecule level [34, 110]. This transition provides a more comprehensive view of isoform usage dynamics and enables the identification of coordinated splicing events across multiple exons.

To address the unique properties of scLR-seq data, a range of computational tools has been developed to quantify isoform-level variation. LongCell [34] is a leading example, integrating barcode recovery, quantification, and a statistical framework for assessing both intra- and inter-cellular splicing diversity, thereby revealing widespread isoform heterogeneity even within highly expressed genes. Scywalker emphasizes an end-to-end design, enabling transcript discovery, quantification, and cell-level demultiplexing directly from raw sequencing data, which provides a scalable solution for large datasets[75]. IsoTools 2.0 offers a comprehensive framework that integrates transcript identification, differential isoform usage, local splicing analysis, and transcription start site prediction, allowing a more global assessment of transcript structural complexity [101]. In addition, recent tools such as LRAA demonstrate improved sensitivity in detecting low-abundance and complex isoforms, supporting exploratory analyses in heterogeneous

tissues [86]. Visualization platforms such as Chevreul further facilitate the interpretation of full-length data by enabling interactive exploration of exon coverage, isoform expression, and splicing patterns [100].

Importantly, the field is rapidly moving beyond descriptive isoform cataloging toward mechanistic interpretation. Emerging frameworks such as scCyclone [99] integrate differential transcript usage (DTU), differential splicing events (DSE), and RNA-binding protein (RBP) motif enrichment within a unified analytical model, aiming to link splicing factor activity with isoform output. At the functional level, tools such as IsoformSwitchAnalyzeR [98] extend this analysis by predicting the downstream consequences of isoform switching, including changes in protein domains, coding potential, and regulatory features. This progression from identifying an event to predicting its functional effect allows researchers to uncover the molecular drivers behind cellular state transitions.

These analytical advances are beginning to yield concrete biological insights. For example, LongCell [34] has demonstrated the detectability of spatial isoform switching and identified candidate regulatory targets in CRISPR-based perturbation of splicing factors, suggesting that scLR can link splicing heterogeneity to upstream regulatory mechanisms. In parallel, studies using ScISOr-Seq2 [23] in brain tissues have revealed extensive isoform variability across brain regions, cell subtypes, and developmental stages, with coordinated regulation at both exon and transcript boundary levels. In many cases, these specific isoform patterns define cellular identity more accurately than gene-level expression alone. scLR uniquely enables this dimension to be captured at full-length, single-molecule, and single-cell resolution, thereby providing a new perspective for dissecting transcriptional regulation.

Transcription Start and End Site Resolution

Transcription start site selection and transcription end site usage are critical regulatory layers that define transcript boundaries and functional potential. Alternative TSS usage can reshape the 5'UTR and influence translation initiation, whereas alternative TES selection modulates 3'UTR length, thereby affecting RNA stability, localization, and translational efficiency [111, 112]. Thus, transcript diversity arises not only from internal alternative splicing but also from coordinated regulation at transcript boundaries. Traditional short-read single-cell RNA sequencing can capture either 5' or 3' end information depending on library design, but it cannot link TSS, exon connectivity, and TES usage within the same RNA molecule. In contrast, long-read sequencing spans full-length transcripts, enabling simultaneous characterization of transcription initiation, splicing, and termination at the single-molecule level. This capability allows the direct observation of coupling among promoter usage, exon connectivity, and 3' end selection in single cells, rather than relying on indirect inference.

At the methodological level, analytical frameworks are shifting from simple "end detection" toward integrated modeling of differential boundary usage and structural coupling. Tools such as scTSS [102] leverage 5' scRNA-seq data to perform TSS clustering, quantification, and differential usage analysis, enabling the identification of cell-type- and condition-specific promoter activity. Meanwhile, methods such as IsoTools 2.0 [101] extend this concept by integrating TSS identification with full-length isoform reconstruction, allowing distinct

promoter-driven transcripts to be directly linked to their corresponding isoform structures and avoiding collapse at the gene level. Alternative polyadenylation (APA) constitutes a complementary regulatory layer. The selection of multiple poly(A) sites can result in 3'UTR shortening or lengthening, or even intronic polyadenylation, thereby influencing interactions with RNA-binding proteins and miRNAs. Recent approaches, such as Ouro-Seq [70], demonstrate that under a full-length transcript framework, alternative promoter usage and APA can be jointly resolved at single-cell resolution. This unified modeling of transcript boundaries elevates TSS and TES regulation to a level comparable to alternative splicing in defining transcriptomic complexity.

These advances are beginning to yield concrete biological insights. For example, enhanced ScISOr-Seq2 [23] analyses in mouse and human brain tissues revealed extensive variability in TSS usage, poly(A) site selection, and exon regulation across brain regions, cell types, and developmental stages. Similarly, Ouro-Seq [70] profiling across multiple adult mouse tissues showed that distinct cell types exhibit coordinated differences not only in isoform expression but also in promoter usage and APA patterns. These findings collectively suggest that TSS and TES are not merely passive boundaries at the ends of a transcript; instead, they are active regulatory nodes that help define cellular identity and developmental programs. Together with alternative splicing, transcription initiation and termination form an integrated and multi-layered regulatory framework that shapes the full landscape of transcriptomic diversity

Somatic Mutations and Allele-Specific Expression

Somatic mutations and ASE represent two complementary layers of information in single-cell transcriptomics. Somatic mutations capture acquired genetic differences among cells and provide insight into clonal evolution, whereas ASE reveals asymmetrical transcriptional activity between the two allelic copies of a gene [113, 114]. Traditional short-read single-cell RNA sequencing faces clear limitations in both aspects: sparse coverage of variant sites hampers reliable discrimination of true somatic mutations from sequencing errors and background polymorphisms, and ASE estimates often rely on only a few heterozygous single-nucleotide polymorphism SNPs, making them sensitive to low coverage, mapping bias, and excessive dispersion. In contrast, long-read sequencing fundamentally expands the available variant information by covering a larger number of SNP sites within individual transcripts. This increased density of informative loci provides a richer set of candidate variants for both somatic mutation detection and ASE analysis. At the same time, long reads preserve full-length transcript structures and exon connectivity, enabling variant information to be interpreted within a complete isoform context [19, 29, 114].

For somatic variant detection, scLR frameworks now provide integrated pipelines that link genetic variation to transcriptional state. Tools such as LongSom can de novo identify somatic single nucleotide variants (SNVs), mitochondrial variants (mtSNVs), copy number alterations (CNAs), and fusion transcripts from long-read single-cell data, reconstructing the clonal heterogeneity of tumors at single-cell resolution [29]. Unlike traditional approaches that infer malignant states indirectly from expression patterns, these strategies incorporate genetic variation directly into cell clustering and subclone identification, enabling joint analysis of transcriptomic state and somatic evolution.

Similarly, ASE analysis benefits substantially from long-read sequencing. By spanning entire

transcripts, long reads reduce dependence on single-exon SNPs and mitigate the effects of sparse sampling and low site coverage. They also allow allelic expression to be linked to full-length transcript structures and novel isoforms. Statistical frameworks such as ASPEN model ASE using beta-binomial distributions with adaptive shrinkage, providing robust estimates in low-count, high-noise settings, and emphasize that ASE should be interpreted as a dynamic, cell-state dependent molecular phenotype rather than a single average bias [106].

Together, these advances in somatic mutation detection and ASE analysis offer richer yet complementary insights into transcript diversity and regulatory potential, facilitating the interpretation of heterogeneity and the identification of candidate cis-regulatory effect.

Genetic Regulation at Transcript Resolution

The significance of transcripts lies not only in their structural novelty, but also in the fact that they can be regulated by genetic variation in a cell-type-specific manner [115, 116]. Previous long-read studies have already shown that accurate full-length annotation can improve the interpretation of disease-linked isoforms and their connections to trQTLs, junction-level sQTLs, and GWAS signals. A recent study further demonstrated that isoQTL analysis could identify numerous genetic effects acting at the isoform level, including signals involving unannotated or cell-type-specific isoforms, many of which extended beyond what could be captured by conventional gene-level expression analysis [117]. By further integrating a negative binomial framework, mashr, and GWAS analyses, this study illustrates that single-cell long-read transcriptomics can not only uncover complex transcripts themselves, but also resolve their genetic regulation at full-length, cell-type-specific resolution, thereby providing a more direct molecular path from risk variants to effector isoforms and their relevant cellular contexts.

Complex Transcripts: Fusions, Transposable Elements, Novel Isoforms, and RNA Modifications

The deeper value of long-read sequencing lies in its ability to resolve "complex transcripts," which are particularly difficult to identify using short-read data. This category includes gene fusion transcripts [105, 118], TE-derived transcripts [30], and a vast number of novel isoforms not yet included in existing annotations [19], and even RNA modifications at isoform resolution. The long-standing underrepresentation of these molecules in single-cell transcriptomics stems not from their biological irrelevance, but from the difficulty of reconstructing their structures from fragmented reads. These structures often contain repetitive sequences, novel combinations of start and termination sites, or non-canonical junctions and splice patterns that resist accurate reconstruction from fragmented reads. In contrast, long-read sequencing can directly span fusion breakpoints, repetitive regions, and full transcript boundaries, enabling these complex events to be observed at single-molecule and single-cell resolution. As a result, complex transcripts are shifting from annotation byproducts to biologically meaningful features that provide additional dimensions for understanding clonal heterogeneity, developmental regulation, and genome evolution [9, 44].

For example, m6A-isoSC-seq [73] integrates 10x Genomics single-cell cDNA libraries with Oxford Nanopore long-read sequencing and APOBEC1-YTH-induced C-to-U mutations, enabling isoform-level m6A detection in individual cells. Similarly, scm6A-seq[119] has revealed dynamic m6A landscapes during mouse oocyte maturation and early embryonic development at true

single-oocyte resolution. As a result, complex transcripts are shifting from annotation byproducts to biologically meaningful features that provide additional dimensions for understanding clonal heterogeneity, developmental regulation, RNA surveillance, and genome evolution.

In the detection of fusion transcripts, the advantages of long-read sequencing are particularly evident. Fusion events are defined by breakpoint-spanning sequences and their associated transcript structures, which are only partially captured by short reads. Recent frameworks like LongFUSE [104] are designed specifically for high-throughput single-cell long-read data to identify full-length fusion isoforms. More established tools, such as CTAT-LR-fusion [105], provide end-to-end pipelines for fusion identification, quantification, annotation and visualization. These studies consistently show that long-read data improve sensitivity and enable resolution of fusion isoforms and their cell-type-specific expression. This allows gene fusions to be treated as detailed structural phenotypes that differ across cell types, rather than simple "present or absent" markers [104, 105].

For TE-derived transcripts, the primary challenge lies in sequence repetitiveness and multi-mapping ambiguity. Previous short-read-based tools, such as scTE [107] and SoloTE [108], have demonstrated that TE expression is dynamic and biologically regulated rather than background noise. However, accurately assigning reads to specific genomic loci remains difficult, especially for evolutionarily young TE families. CELLO-seq [109] represents a major advance in this area by using long UMIs and error-correction strategies to measure full-length molecules reliably. This enables the analysis of locus-specific TE expression and TE-derived isoforms at single-cell resolution. Importantly, this enables the dissection of cell-type-specific TE activity and its contribution to cellular heterogeneity, which is largely inaccessible with bulk or short-read data. Long-read technology effectively moves TE research from a rough description of family-level abundance to a high-resolution, locus-specific, and cell-type-specific characterization.

Similarly, novel isoforms should not be viewed merely as extra entries in a database. Many of these transcripts are actually cell-type-specific regulatory products or read-through transcripts, or entirely new transcript structures shaped by repetitive elements. Recent large-scale studies have shown that unannotated transcripts are widespread across different tissues. For example, the TRAILS atlas of human immune cells identified numerous unannotated isoforms and revealed how repetitive elements contribute to this diversity [115]. Likewise, the Ouro-Seq [70] mouse atlas demonstrated that alternative splicing and polyadenylation together shape the unique transcript repertoires of different cell types. These findings indicate that novel isoforms represent meaningful molecular outputs that shape cell identity and govern regulatory programs.

Applications in Immunology and Beyond

scLR provides a transcript-level panoramic view of transcriptome complexity. Compared with traditional single-cell short-read approaches, long-read can directly capture full-length transcript structures, transcription start and end sites, alternative splicing, and isoform usage. It can also integrate information on somatic mutations and allele-specific expression at single-cell resolution. As a result, the application of scLR extends beyond simple expression quantification. It supports

cell state characterization, developmental trajectory analysis, disease mechanism exploration, and clonal evolution tracking. In biological systems that rely on alternative splicing, promoter choice, 3'-end processing, or receptor rearrangement, the advantages of long-read technology are not merely incremental; rather, they redefine the types of biological questions that can be addressed.

This impact is particularly evident in immunology. Immune cells are highly heterogeneous, and their functional states depend not only on gene expression levels but also on isoform structures, receptor rearrangements, and clonal expansion. The TRAILS atlas, which maps full-length transcripts across 29 peripheral blood immune cell subsets by long-read sequencing, shows widespread unannotated isoforms with clear cell type specific patterns. Alternative 3'-UTR usage contributes to shaping these specific expression profiles. Integrating isoform switch analysis with QTL and GWAS data further highlights isoforms linked to immune-related diseases [115]. Another long-read single-cell study of human PBMCs uncovered numerous novels, cell-type-specific isoforms, revealing isoform diversity within functional immune contexts [120]. Meanwhile, strategies like RAGE-seq [62] enable joint recovery of full-length TCR/BCR sequences and transcriptome data, allowing immune repertoire and transcriptome profiling in the same cell. This framework not only resolves clonal relationships but also links isoform diversity directly to receptor dynamics, providing a finer characterization of activation states and functional differentiation. When combined with multi-omics data such as scTCR/BCR-seq, scLR uncovers regulatory layers beyond traditional gene-level analyses, capturing coordinated alterations in isoforms and immune receptor clonotypes during immunosenescence for a more systematic understanding of immune aging [121]. Overall, scLR is reshaping the molecular framework for understanding immune cell identity, functional states, and disease associations.

Beyond the immune system, fields such as neuroscience, developmental biology, and spatial transcriptomics are also important directions for scLR. In brain tissue, ScISOr-Seq2 [23] studies show that neurons and glial cells exhibit broad isoform variability across brain regions, cell subtypes, and developmental stages. Certain cell populations display pronounced regulation at TSS, poly(A) sites, and exon levels, indicating that full-length isoform structures are key features defining cell identity and developmental programs. In developmental biology, studies on early zebrafish embryos show that cell populations can be distinguished by stage-specific isoform usage [122]. In some cases, these isoform-level profiles provide a more accurate coordinate for cell fate differentiation than traditional gene-level analysis. Furthermore, technologies like Spl-ISO-Seq [33] have brought long-read sequencing into the field of spatial transcriptomics. This has revealed gradients of splicing and polyadenylation across different cortical layers, proving that transcript heterogeneity is organized within specific tissue dimensions.

In cancer research, scLR-seq demonstrates a unique ability to couple genotypes with phenotypes. Methods like scNanoGPS [26] enable the joint analysis of single-cell mutations and transcriptomes without relying on short-read data. This has revealed distinct patterns of isoform usage and mutation distribution between tumor cells and their surrounding immune environment. Additionally, tools like CTAT-LR-Fusion [105] show that long-read sequencing can identify fusion transcripts and complex transcript structures at single-cell resolution, providing precise molecular foundation for tumor classification and targeted therapy. In hematological malignancies, long-read sequencing directly resolves abnormal splicing or novel isoforms associated with drug resistance,

offering molecular explanations for mechanisms such as immune evasion [123]. These advances establish scLR-seq as an essential tool in precision oncology.

Collectively, these applications illustrate that single-cell long-read transcriptomics is moving beyond the technical phase of constructing isoform catalogs. In the immune system, it reveals the synergy between isoform regulation and receptor remodeling. In fields like neuroscience and oncology, it continues to prove that full-length transcript structure is a critical molecular layer for defining cellular identity, spatiotemporal programs, and disease evolution. Ultimately, these advancements are moving the field toward a more complete and functional understanding of the single-cell landscape.

Challenges and Future Directions

Despite the rapid expansion of biological applications, single-cell long-read transcriptomics remains in a transitional stage, its potential has been clearly demonstrated, yet its routine adoption is not fully established. The most immediate limitations arise from the experimental layer. Compared to conventional short-read scRNA-seq, long-read workflows still require trade-offs among throughput, cost, single-molecule accuracy, and full-length molecule recovery [124]. Although platforms such as PacBio and Oxford Nanopore have substantially improved in accuracy and accessibility, long-read single-cell approaches generally remain lower in effective per-cell transcript coverage and usable throughput. In addition, long molecules are more susceptible to degradation, truncation, and amplification bias during reverse transcription and library preparation [10, 125]. Barcode and UMI recovery also remain more challenging due to elevated sequencing error rates, which introduce additional uncertainty in demultiplexing, molecule collapsing, and dropout [14, 28]. As a result, the primary bottleneck is no longer whether full-length transcripts can be captured, but whether they can be profiled in a stable, scalable, and cost-effective manner.

Correspondingly, the complexity of the computational analysis chain represents a second core challenge. Single-cell long-read data is not merely a "long-read version" of bulk sequencing with a cell tag, it requires the simultaneous processing of cell barcode and UMI extraction, error correction, isoform quantification, and downstream tasks such as alternative splicing, TSS/TES, ASE, and fusion detection. Minor biases in the early stages of the pipeline can be amplified at each subsequent step. For instance, high error rates can directly impair barcode recovery, while read truncation leads to inaccurate isoform quantification [24, 34, 120]. Currently, the field lacks a unified and mature statistical framework for robustly assessing splicing variation within and between cells. The challenge is not a lack of tools, but rather a lack of stable, compatible analysis systems that can be easily migrated across different sequencing platforms and library strategies.

Furthermore, the information density provided by long reads complicates statistical interpretation and reproducibility. Signals at the isoform, TSS/TES, or fusion levels are naturally sparser than gene-level counts, making them more dependent on sequencing depth, sample size, and the quality of reference annotations [14]. While the discovery of novel isoforms is a major advantage of long-read sequencing, their biological relevance and reproducibility across cohorts cannot be assumed. Systematic benchmarks have shown significant performance differences

between various bioinformatics workflows in isoform profiling [126]. This indicates that the field is still some distance away from establishing a "gold standard" or "default practice" similar to those found in short-read single-cell analysis. Establishing public benchmark datasets, unified evaluation metrics, and standardized cross-platform protocols will be essential infrastructure for the future.

As these challenges become clearer, future research directions are beginning to converge. Experimentally, the focus will remain on increasing throughput, reducing input requirements, and improving full-length molecule recovery to close the gap with short-read scales. Computationally, the field is likely to move away from isolated task-specific tools toward joint modeling frameworks that simultaneously consider gene expression, isoform usage, and sequence variation. Additionally, the integration of long-read sequencing with short-read data, spatial transcriptomics, and other single-cell multi-omics layers will be a dominant trend [66]. Recent spatial long-read studies have demonstrated that splicing and polyadenylation regulation have clear tissue-spatial dimensions [33, 63], suggesting that the focus is shifting from building isoform catalogs to interpreting full-length structures as regulatory phenotypes. Ultimately, as reference annotations and benchmark systems mature, single-cell long-read sequencing will transition from a "novel technology showcase" into a robust tool for disease mechanism resolution and clinical translation.

Conclusions

scLR-seq has rapidly emerged as a transformative technology, providing an unprecedented molecular view of transcriptomes at the level of full-length isoforms. By directly capturing transcript structure, alternative splicing, and transcription start and end sites, scLR-seq overcomes the inherent limitations of short-read approaches and enables precise characterization of cellular heterogeneity, developmental programs, and disease-associated transcriptomic changes. It has revealed novel isoforms, complex transcript architectures, and regulatory dynamics across immune, neural, and tumor systems, uncovering cell-type-specific splicing, promoter usage, and polyadenylation patterns that were previously inaccessible. These findings highlight the critical role of transcript structural complexity in defining cellular identity and function. While challenges remain in throughput, cost, and data analysis, ongoing developments in computational tools, multi-omics integration, and experimental scale promise to expand its impact. scLR-seq is poised to become a core tool for dissecting cellular programs, developmental trajectories, and disease mechanisms, offering a new level of resolution in transcriptomic research.

Abbreviations

APA: alternative polyadenylation; AS: alternative splicing; ASE: allele-specific expression; CB: cell barcode; CCS: circular consensus sequencing; CNA: copy number alteration; DSE: differential splicing events; DTU: differential transcript usage; EM: expectation–maximization; GWAS: genome-wide association study; LRS: long-read sequencing; LSH: locality-sensitive hashing; ONT: Oxford Nanopore Technologies; PacBio: Pacific Biosciences; QTL: quantitative trait locus; RBP: RNA-binding protein; RNN: Recurrent Neural Network; scLR-seq: single-cell long-read sequencing; scRNA-seq: single-cell RNA sequencing; SMRT: Single-Molecule Real-Time; SNP: single-nucleotide polymorphism; SNV: single nucleotide variants; SNV:

single-nucleotide variants; T2T: telomere-to-telomere; TCC: transcript compatibility count; TE: transposable element; TES: transcription end site; TSS: transcription start site; UMI: unique molecular identifier

Acknowledgements

We thank all of our team members. This work was supported by the National Key Research and Development Program of China (2025YFC3409300), Guangdong Basic and Applied Basic Research Foundation (2026A1515012050 and 2024B1515230003), and the Shenzhen Key Laboratory of Single-Cell Omics (ZDSYS20190902093613831).

Author Contributions

Chuanyu Liu (Conceptualization, Supervision, Writing – review & editing), Ze-Hui Ren (Investigation, Writing – original draft, Visualization), Wenteng Liu (Writing – original draft, Visualization), Jianhua Yin (Writing – review & editing).

Data Availability

not applicable

Competing Interests

The authors declare no competing financial interests.

References

1. Gulati GS, D'Silva JP, Liu Y, et al. Profiling cell identity and tissue architecture with single-cell and spatial transcriptomics. Nature reviews. Molecular cell biology 2025;**26**(1):11-31. doi:10.1038/s41580-024-00768-2.
2. Rood JE, Maartens A, Hupalowska A, et al. Impact of the Human Cell Atlas on medicine. Nat Med 2022;**28**(12):2486-2496. doi:10.1038/s41591-022-02104-7.
3. Yin J, Zheng Y, Huang Z, et al. Chinese Immune Multi-Omics Atlas. Science 2026;**391**(6781):eadt3130. doi:10.1126/science.adt3130.
4. Boxer E, Feigin N, Tschernichovsky R, et al. Emerging clinical applications of single-cell RNA sequencing in oncology. Nature reviews. Clinical oncology 2025;**22**(5):315-326. doi:10.1038/s41571-025-01003-3.
5. Cha J, Lee I. Single-cell network biology for resolving cellular heterogeneity in human diseases. Exp Mol Med 2020;**52**(11):1798-1808. doi:10.1038/s12276-020-00528-0.
6. Grün D, Lyubimova A, Kester L, et al. Single-cell messenger RNA sequencing reveals rare intestinal cell types. Nature 2015;**525**(7568):251-255. doi:10.1038/nature14966.
7. Jindal A, Gupta P, Jayadeva, et al. Discovery of rare cells from voluminous single cell expression data. Nat Commun 2018;**9**(1):4719. doi:10.1038/s41467-018-07234-6.
8. Tirosh I, Suva ML. Cancer cell states: Lessons from ten years of single-cell RNA-sequencing of human tumors. Cancer Cell 2024;**42**(9):1497-1506. doi:10.1016/j.ccell.2024.08.005.
9. Ament IH, Debruyne N, Wang F, et al. Long-read RNA sequencing: A transformative technology for exploring transcriptome complexity in human diseases. Mol Ther 2025;**33**(3):883-894. doi:10.1016/j.ymthe.2024.11.025.

10. Chen Y, Davidson NM, Wan YK, et al. A systematic benchmark of Nanopore long-read RNA sequencing for transcript-level analysis in human cell lines. Nat Methods 2025;**22**(4):801-812. doi:10.1038/s41592-025-02623-4.
11. Pardo-Palacios FJ, Wang D, Reese F, et al. Systematic assessment of long-read RNA-seq methods for transcript identification and quantification. bioRxiv 2023:2023.07.25.550582. doi:10.1101/2023.07.25.550582.
12. Iwabuchi S, Nasti A, Okada H, et al. Systematic evaluation of long- and short-read RNA-seq for human peripheral blood. NAR molecular medicine 2026;**3**(1):ugag006. doi:10.1093/narmme/ugag006.
13. Wen L, Tang F. Single-cell omics sequencing technologies: the long-read generation. Trends Genet 2026;**42**(1):46-62. doi:10.1016/j.tig.2025.07.012.
14. Bhatia S, Field MA, Hebbard L, et al. Bioinformatics frameworks for single-cell long-read sequencing: unlocking isoform-level resolution. Brief Bioinform 2025;**26**(6):bbaf655. doi:10.1093/bib/bbaf655.
15. Kumari P, Kaur M, Dindhoria K, et al. Advances in long-read single-cell transcriptomics. Hum Genet 2024;**143**(9-10):1005-1020. doi:10.1007/s00439-024-02678-x.
16. Wang Y, Zhao Y, Bollas A, et al. Nanopore sequencing technology, bioinformatics and applications. Nat Biotechnol 2021;**39**(11):1348-1365. doi:10.1038/s41587-021-01108-x.
17. Monzó C, Liu T, Conesa A. Transcriptomics in the era of long-read sequencing. Nature reviews. Genetics 2025;**26**(10):681-701. doi:10.1038/s41576-025-00828-z.
18. Pardo-Palacios FJ, Wang D, Reese F, et al. Systematic assessment of long-read RNA-seq methods for transcript identification and quantification. Nat Methods 2024;**21**(7):1349-1363. doi:10.1038/s41592-024-02298-3.
19. Deng E, Shen Q, Zhang J, et al. Systematic evaluation of single-cell RNA-seq analyses performance based on long-read sequencing platforms. J Adv Res 2025;**71**:141-153. doi:10.1016/j.jare.2024.05.020.
20. Gupta P, O'Neill H, Wolvetang EJ, et al. Advances in single-cell long-read sequencing technologies. NAR Genom Bioinform 2024;**6**(2):lqae047. doi:10.1093/nargab/lqae047.
21. Zhang R, Kuo R, Coulter M, et al. A high-resolution single-molecule sequencing-based Arabidopsis transcriptome using novel methods of Iso-seq analysis. Genome Biol 2022;**23**(1):149. doi:10.1186/s13059-022-02711-0.
22. Byrne A, Beaudin AE, Olsen HE, et al. Nanopore long-read RNAseq reveals widespread transcriptional variation among the surface receptors of individual B cells. Nat Commun 2017;**8**:16027. doi:10.1038/ncomms16027.
23. Joglekar A, Hu W, Zhang B, et al. Single-cell long-read sequencing-based mapping reveals specialized splicing patterns in developing and adult mouse and human brain. Nat Neurosci 2024;**27**(6):1051-1063. doi:10.1038/s41593-024-01616-4.
24. Alfonso-Gonzalez C, Hilgers VRE. Elucidating the coordination of RNA processing using short-read and long-read RNA-sequencing methods. Nature reviews. Molecular cell biology 2026;**27**(3):194-212. doi:10.1038/s41580-025-00895-4.
25. Yang A, de Los Santos MR, Kozlenkov A, et al. Cell type-resolved long-read transcriptomics of the human cortex reveals pervasive alternative splicing and disease-relevant isoforms. bioRxiv 2025. doi:10.1101/2025.11.25.690524.
26. Shiau CK, Lu L, Kieser R, et al. High throughput single cell long-read sequencing analyses of same-cell genotypes and phenotypes in human tumors. Nat Commun 2023;**14**(1):4124.

doi:10.1038/s41467-023-39813-7.
27. Byrne A, Le D, Sereti K, et al. Single-cell long-read targeted sequencing reveals transcriptional variation in ovarian cancer. Nat Commun 2024;**15**(1):6916. doi:10.1038/s41467-024-51252-6.
28. Belchikov N, Hsu J, Li XJ, et al. Understanding isoform expression by pairing long-read sequencing with single-cell and spatial transcriptomics. Genome Res 2024;**34**(11):1735-1746. doi:10.1101/gr.279640.124.
29. Dondi A, Borgsmüller N, Ferreira PF, et al. De novo detection of somatic variants in high-quality long-read single-cell RNA sequencing data. Genome Res 2025;**35**(4):900-913. doi:10.1101/gr.279281.124.
30. Marlow SA, Deaville LA, Berrens RV. Long-read RNA sequencing of transposable elements from single cells using CELLO-seq. Nat Protoc 2025;**20**(11):3070-3095. doi:10.1038/s41596-025-01203-2.
31. Lebrigand K, Magnone V, Barbry P, et al. High throughput error corrected Nanopore single cell transcriptome sequencing. Nat Commun 2020;**11**(1):4025. doi:10.1038/s41467-020-17800-6.
32. Yuan CU, Quah FX, Hemberg M. Single-cell and spatial transcriptomics: Bridging current technologies with long-read sequencing. Mol Aspects Med 2024;**96**:101255. doi:10.1016/j.mam.2024.101255.
33. Foord C, Prjibelski AD, Hu W, et al. A spatial long-read approach at near-single-cell resolution reveals developmental regulation of splicing and polyadenylation sites in distinct cortical layers and cell types. Nat Commun 2025;**16**(1):8093. doi:10.1038/s41467-025-63301-9.
34. Fu Y, Kim H, Roy S, et al. Single cell and spatial alternative splicing analysis with Nanopore long read sequencing. Nat Commun 2025;**16**(1):6654. doi:10.1038/s41467-025-60902-2.
35. Wan YK, Hendra C, Pratanwanich PN, et al. Beyond sequencing: machine learning algorithms extract biology hidden in Nanopore signal data. Trends Genet 2022;**38**(3):246-257. doi:10.1016/j.tig.2021.09.001.
36. Pagès-Gallego M, de Ridder J. Comprehensive benchmark and architectural analysis of deep learning models for nanopore sequencing basecalling. Genome Biol 2023;**24**(1):71. doi:10.1186/s13059-023-02903-2.
37. Kasianowicz JJ, Bezrukov SM. On 'three decades of nanopore sequencing'. Nat Biotechnol 2016;**34**(5):481-482. doi:10.1038/nbt.3570.
38. Athanasopoulou K, Michalopoulou VI, Scorilas A, et al. Integrating Artificial Intelligence in Next-Generation Sequencing: Advances, Challenges, and Future Directions. Curr Issues Mol Biol 2025;**47**(6):470. doi:10.3390/cimb47060470.
39. Ling C, Lebeau B, Keong KC, et al. Advanced deep learning strategies in nanopore RNA sequencing. RNA Biol 2026;**23**(1):1-15. doi:10.1080/15476286.2026.2627968.
40. Dorado (2026). Dorado (Version 1.4) https://github.com/nanoporetech/dorado/
41. Wick RR, Judd LM, Holt KE. Performance of neural network basecalling tools for Oxford Nanopore sequencing. Genome Biol 2019;**20**(1):129. doi:10.1186/s13059-019-1727-y.
42. Zhong ZD, Xie YY, Chen HX, et al. Systematic comparison of tools used for m(6)A mapping from nanopore direct RNA sequencing. Nat Commun 2023;**14**(1):1906. doi:10.1038/s41467-023-37596-5.
43. Geyer J, Opoku KB, Lin J, et al. Real-time genomic characterization of pediatric acute leukemia using adaptive sampling. Leukemia 2025;**39**(5):1069-1077. doi:10.1038/s41375-025-02565-y.
44. Wang B, Jia P, Gao S, et al. Long and Accurate: How HiFi Sequencing is Transforming Genomics. Genomics, proteomics & bioinformatics 2025;**23**(1):qzaf003. doi:10.1093/gpbjnl/qzaf003.

45. Wissel D, Mehlferber MM, Nguyen KM, et al. A systematic benchmark of high-accuracy PacBio long-read RNA sequencing for transcript-level quantification. Genome Biol 2026;**27**(1):110. doi:10.1186/s13059-026-03988-1.

46. Baid G, Cook DE, Shafin K, et al. DeepConsensus improves the accuracy of sequences with a gap-aware sequence transformer. Nat Biotechnol 2023;**41**(2):232-238. doi:10.1038/s41587-022-01435-7.

47. Manuel JG, Heins HB, Crocker S, et al. High Coverage Highly Accurate Long-Read Sequencing of a Mouse Neuronal Cell Line Using the PacBio Revio Sequencer. bioRxiv 2023:2023.06.06.543940. doi:10.1101/2023.06.06.543940.

48. Nurk S, Koren S, Rhie A, et al. The complete sequence of a human genome. Science 2022;**376**(6588):44-53. doi:10.1126/science.abj6987.

49. Gupta I, Collier PG, Haase B, et al. Single-cell isoform RNA sequencing characterizes isoforms in thousands of cerebellar cells. Nat Biotechnol 2018:10.1038/nbt.4259. doi:10.1038/nbt.4259.

50. Philpott M, Watson J, Thakurta A, et al. Nanopore sequencing of single-cell transcriptomes with scCOLOR-seq. Nat Biotechnol 2021;**39**(12):1517-1520. doi:10.1038/s41587-021-00965-w.

51. Al'Khafaji AM, Smith JT, Garimella KV, et al. High-throughput RNA isoform sequencing using programmed cDNA concatenation. Nat Biotechnol 2024;**42**(4):582-586. doi:10.1038/s41587-023-01815-7.

52. Sun J, Philpott M, Loi D, et al. Enhancing single-cell transcriptomics using interposed anchor oligonucleotide sequences. Commun Biol 2025;**8**(1):67. doi:10.1038/s42003-025-07474-5.

53. Deserranno K, Callens E, Berrevoet D, et al. Plate-based long-read single cell gene- and isoform transcriptome profiling using scLIS-seq. Research Square 2025. doi:10.21203/rs.3.rs-6217988/v1.

54. You Y, Prawer YDJ, De Paoli-Iseppi R, et al. Identification of cell barcodes from long-read single-cell RNA-seq with BLAZE. Genome Biol 2023;**24**(1):66. doi:10.1186/s13059-023-02907-y.

55. Volden R, Palmer T, Byrne A, et al. Improving nanopore read accuracy with the R2C2 method enables the sequencing of highly multiplexed full-length single-cell cDNA. Proc Natl Acad Sci U S A 2018;**115**(39):9726-9731. doi:10.1073/pnas.1806447115.

56. Cheng O, Ling MH, Wang C, et al. Flexiplex: a versatile demultiplexer and search tool for omics data. Bioinformatics 2024;**40**(3):btae102. doi:10.1093/bioinformatics/btae102.

57. Tian L, Jabbari JS, Thijssen R, et al. Comprehensive characterization of single-cell full-length isoforms in human and mouse with long-read sequencing. Genome Biol 2021;**22**(1):310. doi:10.1186/s13059-021-02525-6.

58. Rebboah E, Reese F, Williams K, et al. Mapping and modeling the genomic basis of differential RNA isoform expression at single-cell resolution with LR-Split-seq. Genome Biol 2021;**22**(1):286. doi:10.1186/s13059-021-02505-w.

59. Fan X, Tang D, Liao Y, et al. Single-cell RNA-seq analysis of mouse preimplantation embryos by third-generation sequencing. PLoS Biol 2020;**18**(12):e3001017. doi:10.1371/journal.pbio.3001017.

60. Liao Y, Liu Z, Zhang Y, et al. High-throughput and high-sensitivity full-length single-cell RNA-seq analysis on third-generation sequencing platform. Cell Discov 2023;**9**(1):5. doi:10.1038/s41421-022-00500-4.

61. Lin J, Xue X, Wang Y, et al. scNanoCOOL-seq: a long-read single-cell sequencing method for multi-omics profiling within individual cells. Cell Res 2023;**33**(11):879-882.

doi:10.1038/s41422-023-00873-5.

62. Singh M, Al-Eryani G, Carswell S, et al. High-throughput targeted long-read single cell sequencing reveals the clonal and transcriptional landscape of lymphocytes. Nat Commun 2019;**10**(1):3120. doi:10.1038/s41467-019-11049-4.
63. Hamraoui A, Onfroy A, Senamaud-Beaufort C, et al. A systematic benchmark of bioinformatics methods for single-cell and spatial RNA-seq Nanopore long-read data. bioRxiv 2025. doi:10.1101/2025.07.21.665920.
64. Pandey AC, Bezney J, Deascanis D, et al. A CRISPR/Cas9-based enhancement of high-throughput single-cell transcriptomics. Nat Commun 2025;**16**(1):4664. doi:10.1038/s41467-025-59880-2.
65. Peng H, Jabbari JS, Tian L, et al. Single-cell Rapid Capture Hybridization sequencing reliably detects isoform usage and coding mutations in targeted genes. Genome Res 2025;**35**(4):942-955. doi:10.1101/gr.279322.124.
66. Pančíková A, Cools R, Eftychiou M, et al. Long-read single-cell genome, transcriptome and open chromatin profiling links genotype to phenotypes. bioRxiv 2025. doi:10.1101/2025.09.08.674950.
67. Hu W, Foord C, Hsu J, et al. Combined single-cell profiling of chromatin-transcriptome and splicing across brain cell types, regions and disease state. Nat Biotechnol 2025:10.1038/s41587-025-02734-5. doi:10.1038/s41587-025-02734-5.
68. Isakova A, Neff N, Quake SR. Single-cell quantification of a broad RNA spectrum reveals unique noncoding patterns associated with cell types and states. Proc Natl Acad Sci U S A 2021;**118**(51):e2113568118. doi:10.1073/pnas.2113568118.
69. Kim HS, Grimes SM, Hooker AC, et al. Single-cell characterization of CRISPR-modified transcript isoforms with nanopore sequencing. Genome Biol 2021;**22**(1):331. doi:10.1186/s13059-021-02554-1.
70. An H, Choi SY, Yi J, et al. Mouse Single-Cell Long-Read Splicing Atlas by Ouro-Seq. bioRxiv 2025. doi:10.1101/2025.01.17.633678.
71. Chang L, Deng E, Wang J, et al. Single-cell third-generation sequencing-based multi-omics uncovers gene expression changes governed by ecDNA and structural variants in cancer cells. Clin Transl Med 2023;**13**(8):e1351. doi:10.1002/ctm2.1351.
72. Long Y, Liu Z, Jia J, et al. FlsnRNA-seq: protoplasting-free full-length single-nucleus RNA profiling in plants. Genome Biol 2021;**22**(1):66. doi:10.1186/s13059-021-02288-0.
73. Ren Z, He J, Huang X, et al. Isoform characterization of m(6)A in single cells identifies its role in RNA surveillance. Nat Commun 2025;**16**(1):5828. doi:10.1038/s41467-025-60869-0.
74. wf-single-cell (2026). wf-single-cell (Version 3.3.4) https://github.com/epi2me-labs/wf-single-cell
75. De Rijk P, Watzeels T, Küçükali F, et al. Scywalker: scalable end-to-end data analysis workflow for long-read single-cell transcriptome sequencing. Bioinformatics 2024;**40**(9):btae549. doi:10.1093/bioinformatics/btae549.
76. Ebrahimi G, Orabi B, Robinson M, et al. Fast and accurate matching of cellular barcodes across short-reads and long-reads of single-cell RNA-seq experiments. iScience 2022;**25**(7):104530. doi:10.1016/j.isci.2022.104530.
77. Wang Q, Bönigk S, Böhm V, et al. Single-cell transcriptome sequencing on the Nanopore platform with ScNapBar. RNA 2021;**27**(7):763-770. doi:10.1261/rna.078154.120.
78. Trull A, Worthey EA, Ianov L. scnanoseq: an nf-core pipeline for Oxford Nanopore single-cell RNA-sequencing. Bioinformatics 2025;**41**(9):btaf487. doi:10.1093/bioinformatics/btaf487.

79. Kovaka S, Zimin AV, Pertea GM, et al. Transcriptome assembly from long-read RNA-seq alignments with StringTie2. Genome Biol 2019;**20**(1):278. doi:10.1186/s13059-019-1910-1.
80. Chen Y, Sim A, Wan YK, et al. Context-aware transcript quantification from long-read RNA-seq data with Bambu. Nat Methods 2023;**20**(8):1187-1195. doi:10.1038/s41592-023-01908-w.
81. Sim A, Ling MH, Chen Y, et al. Isoform-level discovery, quantification and fusion analysis from single-cell and spatial long-read RNA-seq data with Bambu-Clump. bioRxiv 2025. doi:10.1101/2024.12.30.630828.
82. Prjibelski AD, Mikheenko A, Joglekar A, et al. Accurate isoform discovery with IsoQuant using long reads. Nat Biotechnol 2023;**41**(7):915-918. doi:10.1038/s41587-022-01565-y.
83. IsoSeq (2025). IsoSeq (Version 4.3.0) https://github.com/pacificbiosciences/isoseq
84. Kabza M, Ritter A, Byrne A, et al. Accurate long-read transcript discovery and quantification at single-cell, pseudo-bulk and bulk resolution with Isosceles. Nat Commun 2024;**15**(1):7316. doi:10.1038/s41467-024-51584-3.
85. Gao Y, Wang F, Wang R, et al. ESPRESSO: Robust discovery and quantification of transcript isoforms from error-prone long-read RNA-seq data. Sci Adv 2023;**9**(3):eabq5072. doi:10.1126/sciadv.abq5072.
86. Yu H, Georgescu CH, Khorgade A, et al. Accurate strand-specific long-read transcript isoform discovery and quantification at bulk, single-cell, and single-nucleus resolution. bioRxiv 2026:2026.02.12.705617. doi:10.64898/2026.02.12.705617.
87. Volden R, Schimke KD, Byrne A, et al. Identifying and quantifying isoforms from accurate full-length transcriptome sequencing reads with Mandalorion. Genome Biol 2023;**24**(1):167. doi:10.1186/s13059-023-02999-6.
88. Wyman D, Balderrama-Gutierrez G, Reese F, et al. A technology-agnostic long-read analysis pipeline for transcriptome discovery and quantification. bioRixv 2019. doi:10.1101/672931.
89. Tang AD, Felton C, Hrabeta-Robinson E, et al. Detecting haplotype-specific transcript variation in long reads with FLAIR2. Genome Biol 2024;**25**(1):173. doi:10.1186/s13059-024-03301-y.
90. Tang AD, Soulette CM, van Baren MJ, et al. Full-length transcript characterization of SF3B1 mutation in chronic lymphocytic leukemia reveals downregulation of retained introns. Nat Commun 2020;**11**(1):1438. doi:10.1038/s41467-020-15171-6.
91. Zare Jousheghani Z, Singh NP, Patro R. Oarfish: enhanced probabilistic modeling leads to improved accuracy in long read transcriptome quantification. Bioinformatics 2025;**41**(Supplement_1):i304-i313. doi:10.1093/bioinformatics/btaf240.
92. Loving RK, Sullivan DK, Reese F, et al. Long-read sequencing transcriptome quantification with lr-kallisto. PLoS Comput Biol 2025;**21**(12):e1013692. doi:10.1371/journal.pcbi.1013692.
93. Zhang Z, Luo D, Zhong X, et al. SCINA: A Semi-Supervised Subtyping Algorithm of Single Cells and Bulk Samples. Genes (Basel) 2019;**10**(7):531. doi:10.3390/genes10070531.
94. Shao X, Liao J, Lu X, et al. scCATCH: Automatic Annotation on Cell Types of Clusters from Single-Cell RNA Sequencing Data. iScience 2020;**23**(3):100882. doi:10.1016/j.isci.2020.100882.
95. Aran D, Looney AP, Liu L, et al. Reference-based analysis of lung single-cell sequencing reveals a transitional profibrotic macrophage. Nat Immunol 2019;**20**(2):163-172. doi:10.1038/s41590-018-0276-y.
96. Fu R, Gillen AE, Sheridan RM, et al. clustifyr: an R package for automated single-cell RNA sequencing cluster classification. F1000Research 2020;**9**:223. doi:10.12688/f1000research.22969.2.

97. Lim C, An H, Park J. Identification of cell-type-specific, transcriptionally active transposable elements using long-read RNA-sequencing data-based comprehensive annotation. Genomics & informatics 2025;**23**(1):17. doi:10.1186/s44342-025-00048-1.
98. Vitting-Seerup K, Sandelin A. IsoformSwitchAnalyzeR: analysis of changes in genome-wide patterns of alternative splicing and its functional consequences. Bioinformatics 2019;**35**(21):4469-4471. doi:10.1093/bioinformatics/btz247.
99. scCyclone (2025). scCyclone (Version 1.1.0) https://github.com/dawangran/scCyclone
100. Stachelek K, Bhat B, Cobrinik D. Chevreul: an R bioconductor package for exploratory analysis of full-length single cell sequencing. GigaByte 2025;**2025**:gigabyte158. doi:10.46471/gigabyte.158.
101. Bi Y, Lankenau TL, Lienhard M, et al. IsoTools 2.0: Software for Comprehensive Analysis of Long-read Transcriptome Sequencing Data. J Mol Biol 2025;**437**(15):169049. doi:10.1016/j.jmb.2025.169049.
102. Fu S, Li WV. Predicting and comparing transcription start sites in single cell populations. PLoS Comput Biol 2025;**21**(4):e1012878. doi:10.1371/journal.pcbi.1012878.
103. Shulman ED, Elkon R. Cell-type-specific analysis of alternative polyadenylation using single-cell transcriptomics data. Nucleic Acids Res 2019;**47**(19):10027-10039. doi:10.1093/nar/gkz781.
104. Shiau CK, Lin HY, Pan T, et al. Fusion gene discovery in single cells from high throughput long read single cell transcriptomes. bioRxiv 2026:2026.01.13.699333. doi:10.64898/2026.01.13.699333.
105. Qin Q, Popic V, Wienand K, et al. Accurate fusion transcript identification from long- and short-read isoform sequencing at bulk or single-cell resolution. Genome Res 2025;**35**(4):967-986. doi:10.1101/gr.279200.124.
106. Petrova V, Niu M, Vierbuchen TS, et al. ASPEN: Robust detection of allelic dynamics in single cell RNA-seq. PLoS Comput Biol 2025;**21**(12):e1013837. doi:10.1371/journal.pcbi.1013837.
107. He J, Babarinde IA, Sun L, et al. Identifying transposable element expression dynamics and heterogeneity during development at the single-cell level with a processing pipeline scTE. Nat Commun 2021;**12**(1):1456. doi:10.1038/s41467-021-21808-x.
108. Rodríguez-Quiroz RO, Valdebenito-Maturana B. SoloTE for improved analysis of transposable elements in single-cell RNA-Seq data using locus-specific expression. Commun Biol 2022;**5**(1):1063. doi:10.1038/s42003-022-04020-5.
109. Berrens RV, Yang A, Laumer CE, et al. Locus-specific expression of transposable elements in single cells with CELLO-seq. Nat Biotechnol 2022;**40**(4):546-554. doi:10.1038/s41587-021-01093-1.
110. Wang M, Li Y, Wang J, et al. Integrating short-read and long-read single-cell RNA sequencing for comprehensive transcriptome profiling in mouse retina. Genome Res 2025;**35**(4):740-754. doi:10.1101/gr.279167.124.
111. Weber R, Ghoshdastider U, Spies D, et al. Monitoring the 5'UTR landscape reveals isoform switches to drive translational efficiencies in cancer. Oncogene 2023;**42**(9):638-650. doi:10.1038/s41388-022-02578-2.
112. Alfonso-Gonzalez C, Hilgers VRE. (Alternative) transcription start sites as regulators of RNA processing. Trends Cell Biol 2024;**34**(12):1018-1028. doi:10.1016/j.tcb.2024.02.010.
113. Roehrig AL, Hirsch TZ, Pire A, et al. Single-cell multiomics reveals the interplay of clonal evolution and cellular plasticity in hepatoblastoma. Nat Commun 2024;**15**(1):3031. doi:10.1038/s41467-024-47280-x.

114. Qi G, Battle A. Computational methods for allele-specific expression in single cells. Trends Genet 2024;**40**(11):939-949. doi:10.1016/j.tig.2024.07.003.
115. Inamo J, Suzuki A, Ueda MT, et al. Long-read sequencing for 29 immune cell subsets reveals disease-linked isoforms. Nat Commun 2024;**15**(1):4285. doi:10.1038/s41467-024-48615-4.
116. Wu J, Hu W, Li S. Long-read transcriptome sequencing reveals allele-specific variants at high resolution. Trends Genet 2023;**39**(1):31-33. doi:10.1016/j.tig.2022.09.001.
117. Li B, Luong T, Sisay E, et al. Single-cell full-length transcriptome of human lung reveals genetic effects on isoform regulation beyond gene-level expression. bioRxiv 2026. doi:10.64898/2026.03.27.714873.
118. Walter W, Kern W, Stengel A. Long-read single-cell isoform sequencing for cell type-specific detection of genomic rearrangement-dependent and -independent fusion transcripts. Blood 2025.
119. Yao H, Gao CC, Zhang D, et al. scm(6)A-seq reveals single-cell landscapes of the dynamic m(6)A during oocyte maturation and early embryonic development. Nat Commun 2023;**14**(1):315. doi:10.1038/s41467-023-35958-7.
120. Doyle PH, Page ML, Brandon JA, et al. Decoding the human PBMC isonome: Isoform-level resolution with single-cell long-read transcriptomics. bioRxiv 2025. doi:10.1101/2025.10.16.682832.
121. Zheng Y, Ren ZH, Yang Y, et al. Single-Cell Multi-Omics Dissects Transcript Isoform and Immune Repertoire Dynamics in Human Immunosenescence. bioRxiv 2025. doi:10.1101/2025.11.21.689731.
122. Lin X, Wang X, Liu C, et al. Deciphering the Cell-Specific Transcript Heterogeneity and Alternative Splicing during the Early Embryonic Development of Zebrafish. bioRxiv 2024. doi:10.1101/2024.09.08.611790.
123. Sciarrillo R, Wojtuszkiewicz A, Assaraf YG, et al. The role of alternative splicing in cancer: From oncogenesis to drug resistance. Drug Resist Updat 2020;**53**:100728. doi:10.1016/j.drup.2020.100728.
124. Shi H, Zhou Y, Jia E, et al. Bias in RNA-seq Library Preparation: Current Challenges and Solutions. Biomed Res Int 2021;**2021**:6647597. doi:10.1155/2021/6647597.
125. Prawer YDJ, Gleeson J, De Paoli-Iseppi R, et al. Pervasive effects of RNA degradation on Nanopore direct RNA sequencing. NAR Genom Bioinform 2023;**5**(2):lqad060. doi:10.1093/nargab/lqad060.
126. Scoones ALA, Lan Y, Utting C, et al. A comparison of long-read single-cell transcriptomic approaches. bioRxiv 2025. doi:10.1101/2025.07.03.662955.

Click here to access/download;Figure;Figure1.pdf

Figure 1

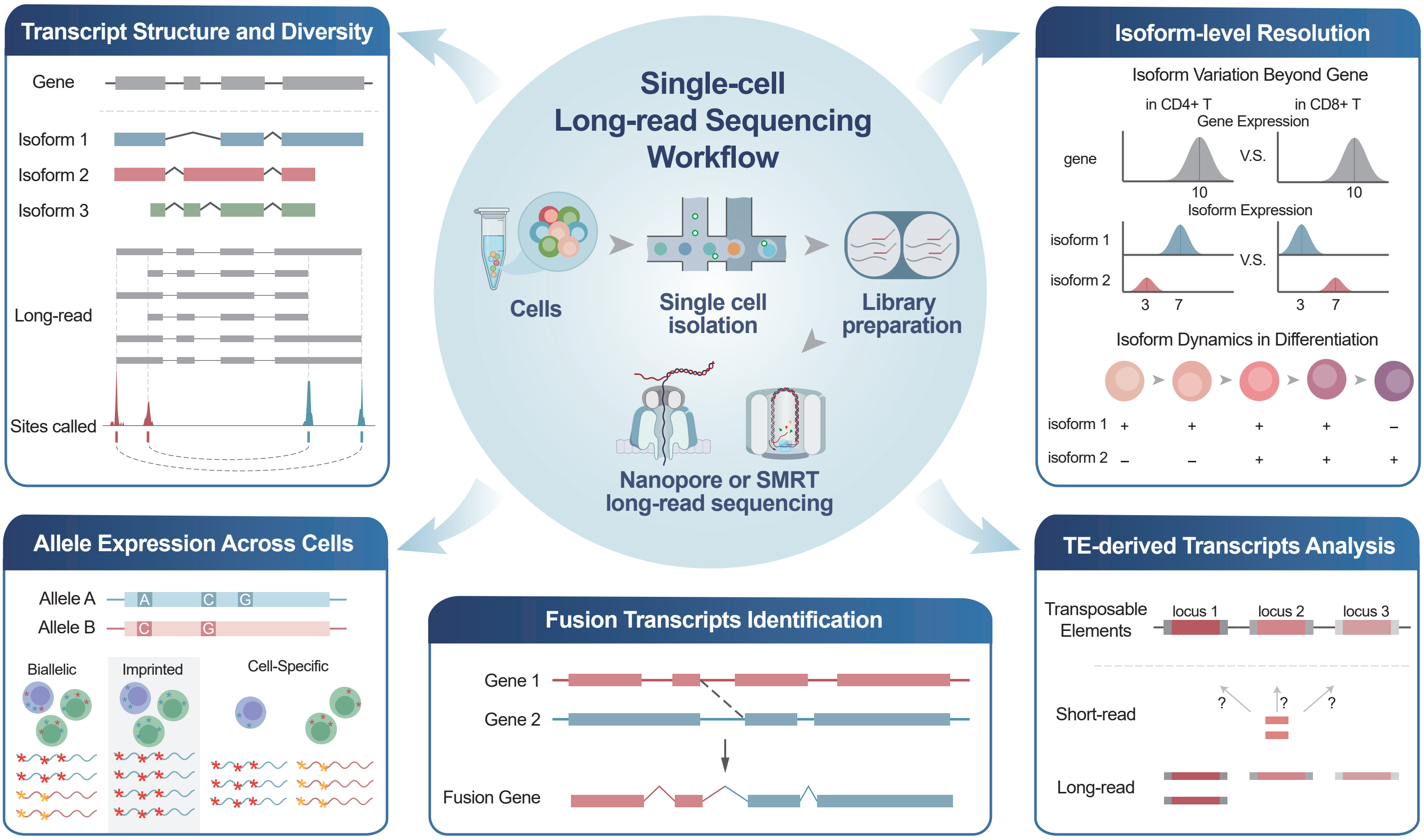

Click here to access/download;Figure;Figure2.pdf

Figure 2

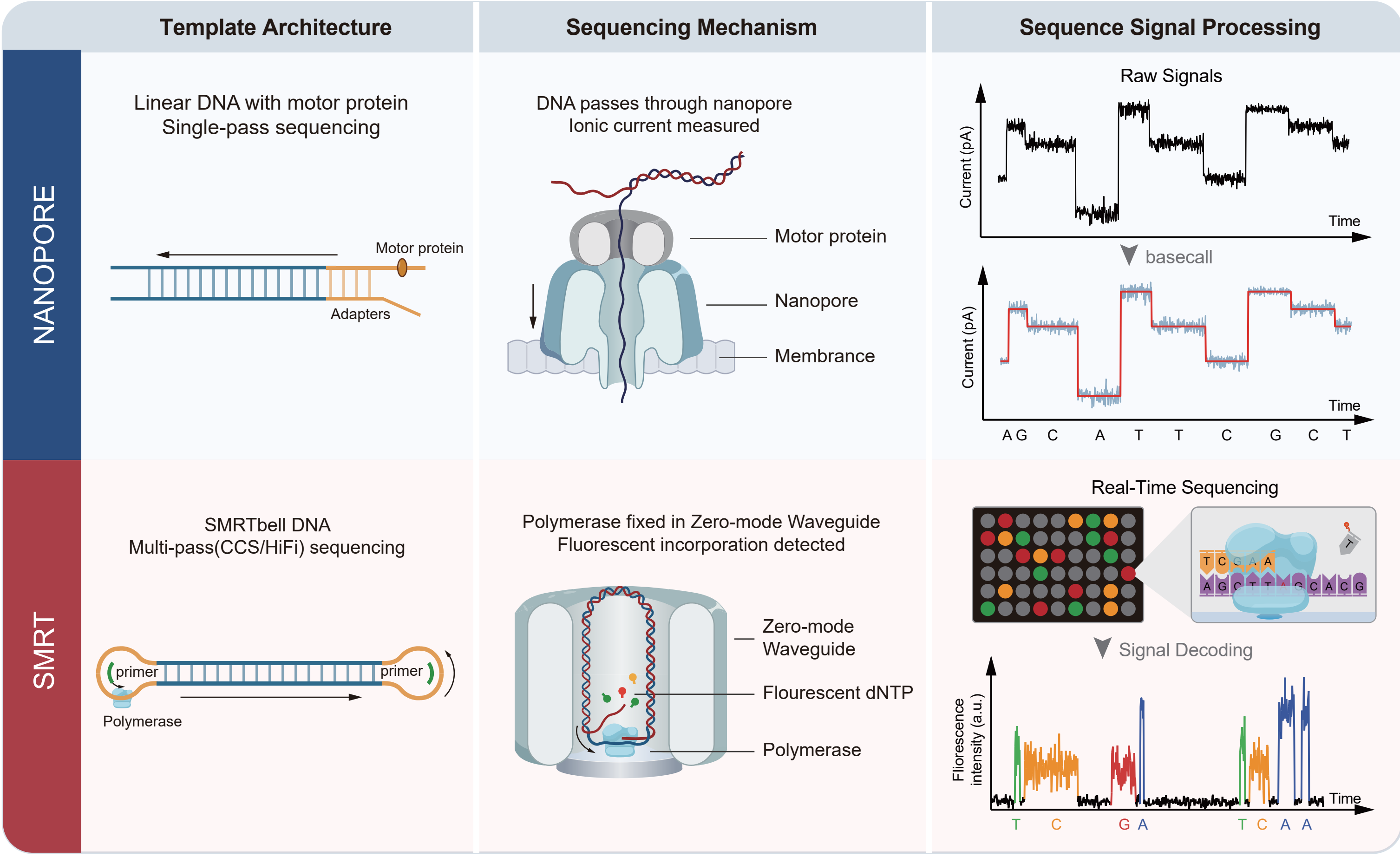

Click here to access/download;Figure;Figure3.pdf

Figure 3

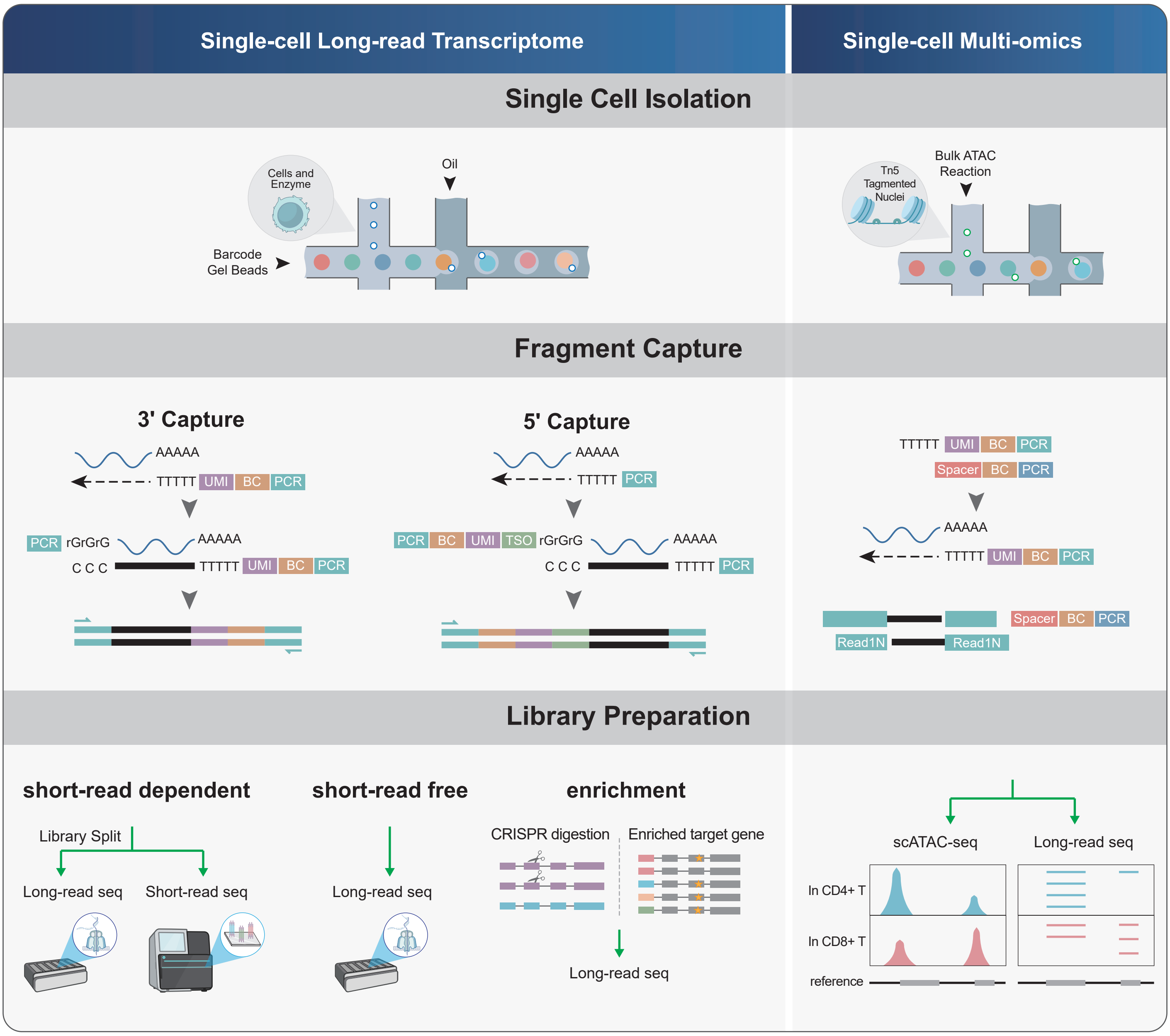

Click here to access/download;Figure;Figure4.pdf

Figure 4

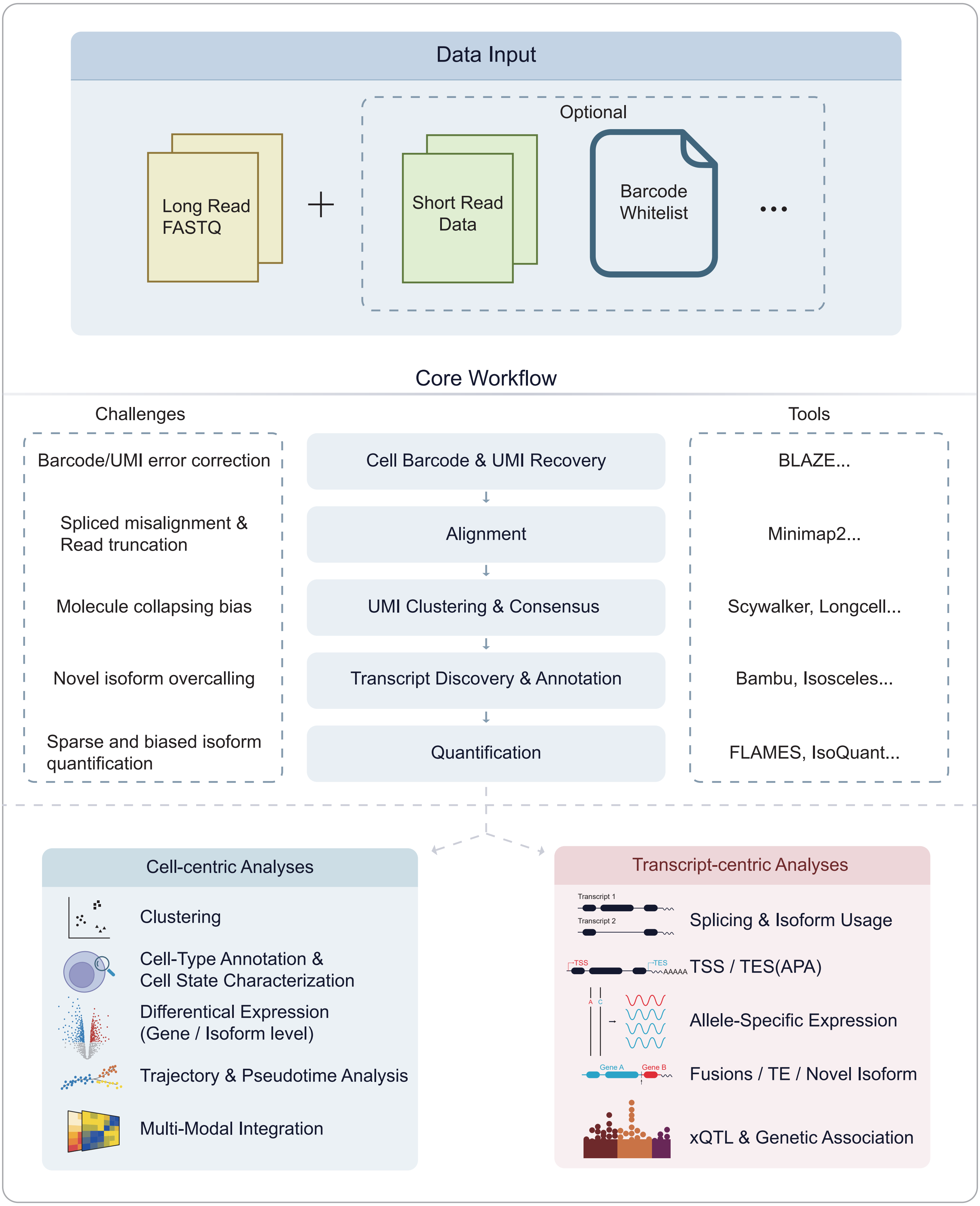

Supplement: Supplementary file 3 [file giag082_giga-d-26-00122_original_submission.pdf]
