# Supplementary material for: Single-cell long-read transcriptomics: from technologies to biological insights
Source: Gigascience. 2026 Jul 22;15:giag082. doi: 10.1093/gigascience/giag082 (PMC13508701; doi:10.1093/gigascience/giag082)
Supplement: Supplementary file 4 [file giag082_giga-d-26-00122_revision_1.pdf]

GigaScience

Single-cell Long-read Transcriptomics: From Technologies to Biological Insights

--Manuscript Draft--

**Manuscript Number:**	GIGA-D-26-00122R1
**Full Title:**	Single-cell Long-read Transcriptomics: From Technologies to Biological Insights
**Article Type:**	Review
**Funding Information:**	
**Abstract:**	The rapid advancement of single-cell long-read transcriptomics (scLR-seq) is transforming transcriptomic research. Unlike traditional approaches, scLR-seq enables the direct capture of full-length transcript structures, isoform usage patterns, and precise information on alternative splicing and transcription start and end sites at single-cell resolution. These capabilities provide an unprecedented molecular view for dissecting cellular heterogeneity, developmental trajectories, and disease mechanisms. This review summarizes the technological evolution of scLR-seq and its potential to illuminate transcriptomic complexity, cellular states, and regulatory networks across key biological domains, including immunology, neurodevelopment, and oncology. We provide a comprehensive overview of the end-to-end analytical workflows, spanning from raw data processing to transcript discovery and quantification. We also discuss downstream biological analyses, with emphasis on cell-type annotation and the resolution of isoform usage dynamics. Finally, we highlight applications of scLR-seq in diverse biological systems, such as the immune system, neural development, and tumor microenvironments, and consider future opportunities and challenges in integrating multi-omics data to decode cellular programs and disease evolution.
**Corresponding Author:**	Chuanyu Liu<br>BGI Group<br>shenzhen, CHINA
**Corresponding Author Secondary Information:**	
**Corresponding Author's Institution:**	BGI Group
**Corresponding Author's Secondary Institution:**	
**First Author:**	Ze-Hui Ren
**First Author Secondary Information:**	
**Order of Authors:**	Ze-Hui Ren
	Wenteng Liu
	Jianhua Yin
	Chuanyu Liu
**Order of Authors Secondary Information:**	
**Response to Reviewers:**	Reviewer1<br>This is a well-structured and timely review that covers the scLR-seq landscape comprehensively. It will be a useful reference for the field, though several areas need strengthening before publication.<br>Response:<br>We thank the reviewer for the positive assessment of our review. We also appreciate the reviewer’s constructive comments and have carefully revised the manuscript to strengthen the areas highlighted below. We also strengthened the previously underdeveloped sections, added quantitative benchmarking evidence, and improved the balance and organization of the manuscript.<br><br>Strengths:

Powered by Editorial Manager® and ProduXion Manager® from Aries Systems Corporation

	The review covers the full pipeline from sequencing platforms to biological applications in a logical sequence. The inclusion of four detailed comparison tables (library strategies, demultiplexing tools, transcript discovery tools, functional analysis tools) is genuinely useful and not commonly found consolidated in a single review. Figures 1-4 are clear and well-designed.<br>Response:<br>We thank the reviewer for the positive comments on the organization, tables, and figures. Following Reviewer 2's suggestion, we have further consolidated the original Figures 2 and 3 into a single integrated figure, as these two figures presented related information that could be more effectively communicated together. This revision reduces redundancy and improves the clarity and coherence of the visual presentation.<br><br>Weaknesses:<br>1. There is a clear copy-paste error on lines 59-60: "These platforms have effectively . These platforms have effectively overcome the fragmentation issues…" More broadly, several sentences in the Background are overly long and would benefit from restructuring for clarity.<br>Response:<br>We thank the reviewer for identifying this editing error. We have removed the repeated phrase and revised the Background to improve sentence structure and readability. In the revised manuscript, the Background now more directly explains why short-read scRNA-seq is insufficient for resolving full-length transcript structures and how long-read sequencing addresses these limitations. We also shortened several overly long sentences to make the introduction easier to follow. These changes have been made in the Background section (line 40-76).<br><br>2. Cell Type Annotation Section is Underdeveloped - The discussion of cell type annotation is notably leaner than other sections. The authors correctly identify the gap, that isoform-level annotation frameworks are lacking, but offer only a brief speculatiion on future directions.<br>Response:<br>We agree with the reviewer that the original discussion of cell type annotation was too brief. We have revised the "Cell Type Annotation" section to provide a more comprehensive and balanced discussion of current annotation strategies, their limitations in the context of single-cell long-read transcriptomics, and potential future directions (line 348-368). Specifically, we now clarify that most existing annotation frameworks were developed for short-read scRNA-seq and operate primarily at the gene-expression level, whereas scLR-seq can capture isoform-level features, including alternative splicing and transcript boundary usage, that may contain cell-type-specific information. We also expanded the discussion of future developments by highlighting the need for multi-layered annotation strategies, isoform-aware reference resources, validated catalogs of cell-type-specific isoforms, and benchmarking frameworks to evaluate platform reliability and annotation robustness. These revisions provide a more detailed account of both the current gap and the methodological directions needed to support isoform-based cell type annotation.<br><br>3. Computational Challenges Section Needs Quantitative Grounding - The challenges discussion reads somewhat qualitatively. Phrases like "relatively high base-level error rates" and "lower in effective per-cell transcript coverage" would benefit from concrete numbers (ONT for example was often previously seen as very error prone but that's no longer the case - more recent papers have these numbers), at least as approximate benchmarks, to help readers appreciate the magnitude of the problems. Citing the systematic benchmarking studies already referenced more specifically here would help.<br>Response:<br>We agree with the reviewer that the original challenges discussion was too qualitative. We have revised the computational sections to include more quantitative context from recent benchmarking and systematic evaluation studies. Specifically, in the "Cell Barcode and UMI Demultiplexing" section, we now state that nanopore raw-read accuracy has improved from approximately 95% with earlier R9 chemistries to over 99% with recent R10.4.1 chemistry, while emphasizing that a typical 10-20 bp cell

barcode can still have an estimated 10-18% probability of containing at least one sequencing error. We also added benchmark-based discussion of barcode recovery performance, including the approximate 92% recall of matched short-read reference barcodes across evaluated methods, together with method-specific trade-offs in precision, recall, UMI correction, and sequencing chemistry. In the “Transcript Discovery and Quantification” section, we further added quantitative and benchmark-based discussion of barcode assignment rates, per-cell read depth, truncation bias, and the relative contributions of read length, read accuracy, and sequencing depth to transcript model reliability. These revisions provide a more concrete assessment of the magnitude of current computational challenges and are included in line 245-284 and line 287-339.

4. Inconsistent Depth Across Biological Applications - The immunology subsection is noticeably more developed than neuroscience and oncology. The neurodevelopment discussion leans heavily on a single study (ScISOr-Seq2), and the oncology discussion is brief. The abstract promises substantial coverage of all three domains; the body of the review should be improved.

Response:

We thank the reviewer for pointing out this imbalance. We have revised the applications section to improve the balance among immunology, neurodevelopment, and oncology, and to better match the scope promised in the Abstract. The revised section now presents these applications as examples of how scLR-seq can reveal transcript-structure variation across biological systems rather than as an uneven list of individual studies. We expanded the neurodevelopment discussion beyond a single study by emphasizing cell-type-resolved splicing, promoter usage, and polyadenylation regulation in neural systems, and we expanded the oncology discussion to highlight how fusion transcripts, allele-specific expression, somatic variants, and isoform diversity can contribute to tumor heterogeneity and disease interpretation. These revisions are located in the “Applications in Immunology and Beyond” section (line 567-620).

5. Multi-omics Integration is Underrepresented - Both the abstract and cover letter emphasize multi-omics integration as a key future direction, yet the actual discussion is dispersed and thin. A dedicated subsection or at minimum a summary table of tools like SPLONGGET, ScISOr-ATAC, and scGTP-seq, would make this a more actionable resource for readers planning such experiments.

Response:

We thank the reviewer for this helpful comment. We agree that multi-omics integration required more explicit treatment. We have added a dedicated “Multi-omics integration” subsection to consolidate the discussion that was previously dispersed across the manuscript (line 542-565). The revised subsection now explains why integrating full-length transcript information with chromatin, spatial, genomic, and other single-cell modalities is important for interpreting cell-state regulation and disease mechanisms. We also discuss recent long-read single-cell multi-omics approaches, including scNanoCOOL-seq, SPLONGGET, and ScISOr-ATAC, and clarify that the field still lacks mature library preparation protocols and standardized computational pipelines for joint interpretation of transcript expression, isoform usage, chromatin accessibility, and genetic variation (line 556-559). In addition, we expanded the Table 5 to include multi-omics methods such as scNanoCOOL-seq, SPLONGGET, ScISOr-ATAC, and scGTP-seq, thereby making the revised manuscript more useful as an actionable resource for readers planning multi-omics experiments.

6. Missing Critical Discussion: Benchmarking and Reproducibility - The review mentions benchmark studies but does not synthesise what those benchmarks actually found. A paragraph summarizing key take-home messages from existing benchmarks would substantially increase the practical value of this review.

Response:

We agree with the reviewer and have expanded the benchmarking and reproducibility discussion to provide clearer synthesis rather than simply citing existing benchmark studies. In the revised manuscript, we now summarize key take-home messages from available benchmarks and quantitative evaluations across the platform-selection and

Powered by Editorial Manager® and ProduXion Manager® from Aries Systems Corporation

computational-analysis workflow. In the “Long-Read Platform Selection for scLR-seq” section, we added a more explicit comparison of ONT and PacBio HiFi as complementary platforms and framed platform choice as a multi-parameter optimization problem involving per-read accuracy, molecule throughput per cell, barcode/UMI robustness, and cost per informative transcript. In the “Cell Barcode and UMI Demultiplexing” section, we discuss benchmark findings on cell barcode and UMI recovery, method-specific differences in sensitivity and precision, and the influence of sequencing chemistry on UMI correction. In the “Transcript Discovery and Quantification” section, we summarize benchmark evidence indicating that read length and read accuracy can affect transcript model reliability more strongly than sequencing depth alone, and we further discuss the trade-off between discovery breadth and model specificity. We also expanded the “Challenges and Future Directions” section to address reproducibility more explicitly, noting that novel isoform discovery requires careful validation, that isoform-level signals are sparser than gene-level counts, and that public benchmark datasets, unified evaluation metrics, and standardized cross-platform protocols remain essential infrastructure for the field. These revisions appear in lines 93-143, line 245-284, line 287-345 and line 622-656.

7. RNA Modifications Section is Abrupt - The paragraph introducing m6A-isoSC-seq and scm6A-seq appears mid-section on "Complex Transcripts" and feels appended rather than integrated. RNA modifications arguably deserve either a dedicated subsection or a more deliberate placement with proper framing.
Response:
We sincerely thank the reviewer for this insightful comment. We have reorganized the manuscript so that RNA modifications are now discussed in a dedicated “RNA Modifications” subsection rather than appearing abruptly within the discussion of complex transcripts (line 456-479). The revised section first introduces m6A as an additional regulatory layer of transcriptome biology and explains why bulk approaches can obscure cell-to-cell heterogeneity in RNA modification profiles. We then discuss current single-cell long-read strategies for m6A detection, such as m6A-isoSC-seq, in a more deliberate methodological context. We also added clearer framing of current limitations, including cDNA dependence, barcode retention, sensitivity, throughput, and the need for future methods that can jointly measure transcript structure and RNA modification in individual cells.

Reviewer2
The manuscript by Ren et al presents an overview of the single cell long reads transcriptomics approaches. The manuscript is timely owing to the rapid development of the technologies and field of applications. Long read transcriptomics has massive potential however as an extremely rapidly developing field it can be difficult to access for a non expert. The availability of reviews of the field including technologies, libraries preparation, and computational approaches can contribute in democratizing such approaches. In the current manuscript the authors present a thorough and well organised overview of long-read single-cell transcriptomics approaches.
Response:
We sincerely thank the reviewer for the positive and constructive assessment of our manuscript. We are encouraged that the reviewer recognizes the timeliness of this topic and the potential value of a review that summarizes technologies, library preparation strategies, and computational approaches for single-cell long-read transcriptomics. In response to the reviewer’s detailed comments below, we have comprehensively revised the manuscript to improve its focus, organization, accessibility, and practical value for both specialists and non-expert readers. The overall scope of the review remains unchanged, but the revised version now provides a more coherent framework for platform selection, library design, computational analysis, benchmarking, biological applications, and future directions.

Nevertheless, the manuscript is currently very verbose and the target audience is unclear, the work is too general / unfocussed for an experienced audience, but includes perhaps more than necessary do the detriment of it's length for non-specialist audience. The manuscript would gain in being substantially shortened and more

focused. For instance figures 2 and 3 are rather uninformative for the objective of the manuscript. Those present the sequencing technologies and integration.
Response:
We sincerely thank the reviewer for this insightful comment. The observation is highly accurate. During the initial drafting, the manuscript gradually shifted toward a method-by-method listing, which deviated from our original intent.
In reversion, we have revised the manuscript to provide a clear, concise overview of single-cell long-read transcriptomics that emphasizes the overall logic of the field and highlights key challenges, with the goal of helping scRNA-seq researchers rapidly understand current approaches. Specifically, we removed detailed method descriptions and consolidated similar methods into broader categories. For each section, we clarified the critical issues that researchers need to consider. Figures 2 and 3 have been integrated and redesigned to represent a decision-making workflow, allowing readers to quickly select appropriate platforms and single-cell long-read strategies. We believe these revisions substantially improve focus, readability, and practical value for the intended audience.

Likewise the authors should pay close attention to the contents of the tables, as such Table 1 that is focusing on library constructions, also include software and pipelines (Sockeye, scNonoGPS, Flexiplex) obfuscating the message from this table. This is also reflected in the text as lines 232-240 deal with algorithms in a section focusing on scLR-Seq library construction. I invite the authors in bringing clearer structure to the manuscript as this will bring greater focus and potential for the readers.
Response:
We thank the reviewer for this comment. We recognize that Table 1 previously mixed library construction protocols with associated computational methods, which could obscure the message. In the original draft, we found this difficult to fully disentangle. Single-cell long-read approaches that do not rely on short-read data follow two main strategies, either optimizing barcode design or optimizing computational methods. And new computational methods often come with specific library design considerations. As a result, Table 1 originally included some library schemes associated with computational method optimization.
To address this concern, we have revised the manuscript to clarify this structure, and all computational-method-associated library schemes have now been moved to the "Computational Methods" section for further discussion, making the separation between library construction and algorithmic optimization clearer.

Specific Comments
- Line 60. Repeated and meaningless sentence with incorrect citations. Assuming an editing error.
Response:
We thank the reviewer for catching this editing error. We have removed the repeated sentence and checked the surrounding citations for consistency. This correction was made in the Background section. We also reviewed the manuscript more broadly to identify similar editing artifacts.

- Line 89-92. Has AI been used in this document? This has a language stands out quite differently to the surrounding copy.
Response:
We thank the reviewer for this comment. The content reflects our intended message: single-cell long-read sequencing is no longer merely a supplement to short-read approaches, but can independently resolve questions that short reads cannot, such as detecting alternative splicing, fusion genes, allele-specific expression, and other transcriptomic features relevant in immunology, cancer, and related fields. Because it integrates multiple layers of transcriptomic information the description emphasizes the broader impact, which is why the wording is more expansive. All scientific content and conclusions are authored by us and have been reviewed for accuracy and consistency with the overall manuscript.
We have also provided a summary of AI-assisted writing in the "Declaration of Generative AI and AI-assisted Technologies in the Manuscript Preparation Process" (line 686-690).

Powered by Editorial Manager® and ProduXion Manager® from Aries Systems Corporation

	- Fig1. Editing box around "Allele expression Across Cells". The document has a large number of mistakes and errors like this suggesting closer attention to editing is required.<br>Response:<br>We apologize for the editing oversight and thank the reviewer for pointing it out. We have corrected the Figure 1 labeling issue around “Allele expression Across Cells” and reviewed the figure for similar formatting problems. In addition, we performed a broader language and formatting check of the manuscript to remove repeated phrases, inconsistent wording, and residual editing artifacts. We have also carefully inspected the final Word and PDF proofs to ensure that the figures, tables, and text are clean and consistent before resubmission.
**Additional Information:**	
**Question**	**Response**
Are you submitting this manuscript to a special series or article collection?	No
**Experimental design and statistics**<br><br>Full details of the experimental design and statistical methods used should be given in the Methods section, as detailed in our Minimum Standards Reporting Checklist. Information essential to interpreting the data presented should be made available in the figure legends.<br><br>Have you included all the information requested in your manuscript?	No
If not, please give reasons for any omissions below.<br><br>as follow-up to "**Experimental design and statistics**<br><br>Full details of the experimental design and statistical methods used should be given in the Methods section, as detailed in our Minimum Standards Reporting Checklist. Information essential to interpreting the data presented should be made available in the figure legends.<br><br>Have you included all the information requested in your manuscript?<br><br>"	Not applicable. This manuscript is a review article and does not include original experimental design or statistical analyses.

**Resources**<br><br>A description of all resources used, including antibodies, cell lines, animals and software tools, with enough information to allow them to be uniquely identified, should be included in the Methods section. Authors are strongly encouraged to cite Research Resource Identifiers (RRIDs) for antibodies, model organisms and tools, where possible.<br><br>Have you included the information requested as detailed in our Minimum Standards Reporting Checklist?	No
If not, please give reasons for any omissions below.<br><br>as follow-up to "**Resources**<br><br>A description of all resources used, including antibodies, cell lines, animals and software tools, with enough information to allow them to be uniquely identified, should be included in the Methods section. Authors are strongly encouraged to cite Research Resource Identifiers (RRIDs) for antibodies, model organisms and tools, where possible.<br><br>Have you included the information requested as detailed in our Minimum Standards Reporting Checklist?<br><br>"	Not applicable. This manuscript is a review article and does not include original resources.
**Availability of data and materials**<br><br>All datasets and code on which the conclusions of the paper rely must be either included in your submission or deposited in publicly available repositories (where available and ethically appropriate), referencing such data using a unique identifier in the references and in	No

Powered by Editorial Manager® and ProduXion Manager® from Aries Systems Corporation

the “Availability of Data and Materials” section of your manuscript.<br><br>Have you have met the above requirement as detailed in our Minimum Standards Reporting Checklist?	
If not, please give reasons for any omissions below.<br><br>as follow-up to "**Availability of data and materials**<br><br>All datasets and code on which the conclusions of the paper rely must be either included in your submission or deposited in publicly available repositories (where available and ethically appropriate), referencing such data using a unique identifier in the references and in the “Availability of Data and Materials” section of your manuscript.<br><br>Have you have met the above requirement as detailed in our Minimum Standards Reporting Checklist?<br><br>"	Not applicable. This manuscript is a review article and does not include original data.
GigaScience has policies and guidelines in place for the use of generative AI-writing tools such as ChatGPT. If you have used such writing tools to assist with writing the manuscript this must be declared and cited in the text. Authors should not list AI-writing tools and other AI-assisted technologies as an author or co-author and should acknowledge that they are fully responsible for text generated or refined by AI-writing tools.<p><br><br>A summary of use (particularly in the introduction or among methods) needs to be included at the end of the paper, and the outputs should also be included as a supplementary file hosted in GigaDB or other open repositories. Please read our guidelines for more information. <p>	No

By submitting to GigaScience, you are aware of the journal's AI-writing tools policy, and if you have declared use of such tools below, you have acknowledged this where appropriate in your manuscript and have made a summary of use and outputs available. <p> <b>AI-assisted writing tools have been used in the preparation of this manuscript?	

Manuscript Click here to access/download;Manuscript;Manuscript.pdf

Single-cell Long-read Transcriptomics: From Technologies to Biological Insights

Ze-Hui Ren[1,2]†, Wenteng Liu[1,3]†, Jianhua Yin[4,5] & Chuanyu Liu[4,5]*

[1]State Key Laboratory of Genome and Multi-omics Technologies, BGI Research, Shenzhen 518083, China

[2]Shenzhen Key Laboratory of Single-Cell Omics, BGI Research, Shenzhen 518083, China.

[3]National Engineering Research Center for Nanomedicine, College of Life Science and Technology, Huazhong University of Science and Technology, Wuhan 430074, China

[4]Shenzhen Proof-of-Concept Center of Digital Cytopathology, BGI Research, Shenzhen 518083, China

[5]Shanxi Medical University-BGI Collaborative Center for Future Medicine, Shanxi Medical University, Taiyuan 030001, China

†Contributed equally to this work

*Corresponding author (Chuanyu Liu, email: liuchuanyu@genomics.cn)

Abstract

Single-cell long-read transcriptomics (scLR-seq) extends single-cell analysis beyond gene abundance by resolving full-length transcript structures in individual cells. It can directly interrogate isoform usage, alternative splicing, and transcription start and end site selection, thereby revealing regulatory variation that is often obscured by short-read measurements. In this review, we examine the experimental and computational foundations of scLR-seq, including platform selection, library design, cell barcode and unique molecular identifier (UMI) recovery, transcript discovery, and isoform quantification. We discuss how these choices influence the reliability of downstream biological interpretation, and summarize emerging insights into isoform usage, alternative splicing, transcription start and end site selection, allele-specific expression, fusion transcripts, transposable element-derived transcripts, and RNA modifications. Finally, we highlight applications of scLR-seq in diverse biological systems, such as the immune system, neural development, and tumor microenvironments, and consider future opportunities and challenges in integrating multi-omics data to decode cellular programs and disease evolution.

Keywords

Single-cell long-read transcriptomics; Full-length isoform resolution; Long-read sequencing; scLR-seq analytical workflows; Transcript structural complexity; Immune profiling

Background

Over the past decade, single-cell transcriptomics has transformed the study of cellular heterogeneity, developmental trajectories, and disease-related state transitions [1-4]. Short-read single-cell RNA sequencing (scRNA-seq) is widely used to identify rare cell populations and analyze dynamic cell states, because it provides high throughput and quantitative stability [5-8]. However, short reads usually sample only part of each transcript, limiting the direct resolution of full-length transcript structures. Features such as alternative splicing, transcription start site (TSS) selection, and transcription end site (TES) choice must often be inferred rather than observed directly. Detection of fusion transcripts or allele-specific expression is also constrained by the inability to link distant variants on the same molecule [9-12]. These limitations matter because cell states can be defined not only by gene expression, but also by the isoforms produced, the boundaries selected, and the genetic variants linked within individual RNA molecules [13-15].

Long-read sequencing (LRS) provides a route to addressing these limitations. Pacific Biosciences (PacBio) high-accuracy circular consensus sequencing (CCS) generates high-accuracy reads, whereas Oxford Nanopore Technologies (ONT) platforms support real-time nanopore sequencing of RNA or cDNA molecules. Both approaches can capture full-length transcripts at the single-molecule level and reduce the fragmentation inherent to short-read technologies [12, 16-18]. In single-cell studies, this capability shifts analysis from gene-level abundance towards transcript-structure-resolved regulation. Full-length reads can reduce ambiguity in transcript reconstruction and enable direct analysis of isoform usage, promoter and polyadenylation choice, fusion structure, allele-specific transcription and unannotated transcripts across heterogeneous cell populations.

Advances in sequencing accuracy, cell-barcoding strategies and demultiplexing algorithms are moving scLR-seq beyond proof-of-concept studies towards increasingly systematic application [13, 15, 19]. Nevertheless, scLR-seq remains sensitive to experimental and analytical design. Platform choice, cDNA or direct RNA strategy, barcode architecture, molecule recovery, error correction, isoform annotation and quantification models can each determine whether apparent transcript diversity reflects biology or technical artefact. The central question is therefore not simply whether full-length transcripts can be captured in individual cells, but how reliably experimental and computational choices support isoform-level inference.

In this review, we examine scLR-seq from this design-to-interpretation perspective. We first consider major sequencing platforms, library construction strategies, barcode and UMI recovery methods, and computational workflows for transcript discovery and quantification. We then summarize biological insights into isoform usage, alternative splicing, transcript boundary selection, allelic variation, fusion transcripts, transposable element-derived transcripts, and RNA modifications. Finally, we discuss applications in development, immunity and cancer, and consider how integration with short-read scRNA-seq, spatial transcriptomics and other modalities may support more rigorous interpretation of cell-state regulation and disease evolution.

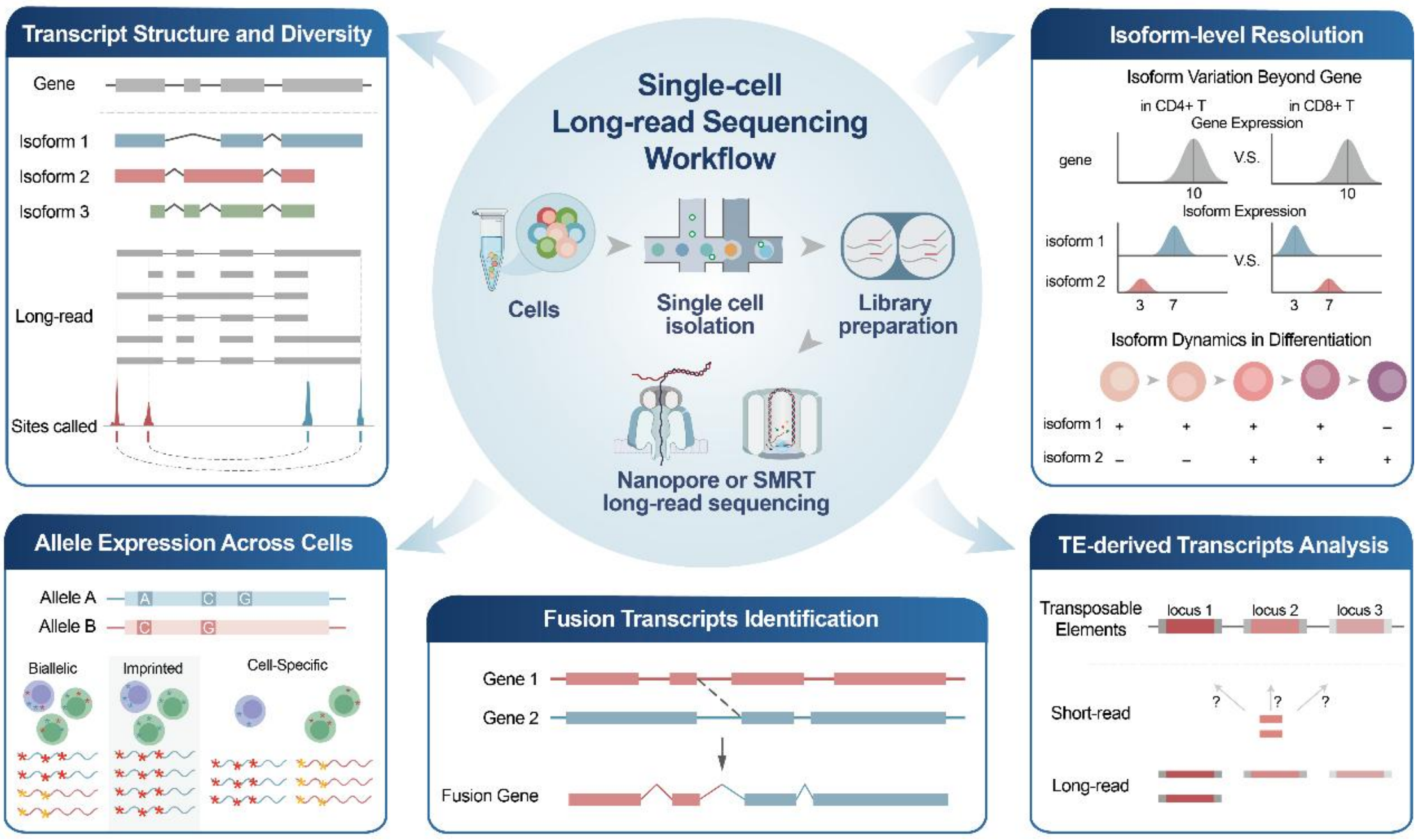

Figure 1. Overview of single-cell long-read RNA sequencing and its analytical applications. Single-cell long-read sequencing enables isolation of individual cells, library preparation, and sequencing on Nanopore or PacBio SMRT platforms. This approach captures full-length transcripts, allowing reconstruction of isoform structures, exon connectivity, and transcription start/end sites, as well as quantification of isoform diversity. It provides isoform-level resolution for assessing cell-type-specific expression and dynamic changes during differentiation. Full-length reads with cell barcodes and UMIs enable allele-specific expression analysis, revealing imprinting and allelic biases across cell populations. Fusion transcripts can be unambiguously detected, defining both fusion partners and their full-length structures. Long reads also facilitate analysis of transposable element-derived transcripts, resolving locus-specific expression and integration into novel isoforms. Together, these capabilities provide a comprehensive molecular view of transcriptomic complexity at single-cell resolution.

Single-Cell Long-Read Transcriptome Sequencing Technologies

Long-Read Platform Selection for scLR-seq

For scLR-seq, platform selection is primarily determined by the biological question and the required balance among read accuracy, transcript length, throughput, cost, and compatibility with downstream barcode and UMI recovery. Current long-read sequencing is dominated by two major technological frameworks: Pacific Biosciences circular consensus sequencing (CCS), which generates highly accurate HiFi reads through repeated sequencing of circularized templates [20, 21], and Oxford Nanopore Technologies (ONT), which detects nucleotide sequences from ionic current changes as nucleic acids pass through nanopores [22]. Emerging nanopore platforms, including MGI CycloneSEQ, QitanTech QNome, and Polyseq Biotech Polyseq, are further diversifying the long-read sequencing landscape. Across platforms, advances in sequencing chemistry, instrument throughput, and deep-learning-based signal processing have increased the utility of long-read data for single-cell transcriptomics [23, 24].

Nanopore-based platforms are attractive for scalable and flexible scLR-seq workflows. ONT sequencing supports ultra long reads, real-time data generation, adjustable throughput, and direct

RNA sequencing, making it useful for transcriptome-wide isoform discovery, rapid profiling, and studies that aim to retain native RNA signals. Recent basecalling frameworks, including Dorado [25], have improved raw-read accuracy through deep learning, while related signal-level methods support modification detection [26] and adaptive sampling [27]. These capabilities favor studies of transcript structure, RNA modification and rapid or cost-sensitive profiling [22]. However, Nanopore-based scLR-seq remains vulnerable to errors in CBs and UMIs, whose inaccurate recovery can cause cell misassignment, UMI inflation, or molecule loss. Therefore, Nanopore-based workflows usually require robust barcode correction, UMI collapsing, and transcript-level error-control strategies.

PacBio CCS-based platforms sequence SMRTbell templates repeatedly to generate high-accuracy HiFi reads. In scLR-seq, this per-read accuracy is particularly valuable for analyses requiring reliable base-level information, including allele-specific expression, expressed variant detection, somatic mutation analysis and precise interpretation of fusion breakpoints [12, 28]. Although HiFi reads are generally shorter than the longest nanopore reads, they span most full-length eukaryotic transcripts, and newer PacBio instruments have improved throughput for isoform-resolved single-cell studies [29]. Because PacBio CCS accuracy is achieved by repeated sequencing of the same molecule, the number of independent molecules recovered per unit of sequencing output and cost should be carefully considered. This issue is especially relevant for large cell numbers, low-abundance isoforms, shallow per-cell coverage, and studies requiring broad transcriptome discovery.

The two major TGS platforms provide complementary but asymmetric advantages for scLR-seq. Rather than a binary preference, platform selection can be formalized as a multi-parameter optimization problem involving four primary dimensions: per-read accuracy, molecule throughput per cell, barcode/UMI robustness, and cost per informative transcript. ONT nanopore sequencing provides high flexibility in throughput and experimental design, supports ultra-long reads and direct RNA sequencing, and is therefore well suited for discovery-oriented studies that prioritize isoform diversity, transcript structure exploration, and large-scale cell sampling [12, 14]. However, error propagation in short sequence tags such as CBs and UMIs remains a limiting factor, requiring dedicated correction and collapsing strategies to ensure accurate molecule counting [16, 30]. PacBio HiFi sequencing provides substantially higher per-read accuracy and more stable base-level resolution, making it preferable for applications requiring precise sequence interpretation, including allele-specific expression, variant detection, and fusion breakpoint resolution [9]. This accuracy is achieved at the cost of reduced molecule throughput per unit sequencing output, which becomes a critical constraint in large-scale single-cell designs. Consequently, ONT is typically favored for high-dimensional discovery and population-scale profiling, whereas PacBio is better suited for precision-driven analyses where molecular correctness outweighs coverage breadth.

scLR-seq Library Construction Strategies

A central challenge in single-cell long-read sequencing is the accurate recovery and assignment of CBs and UMIs. scLR-seq must preserve the association between each full-length transcript molecule and its cell of origin. Because CBs and UMIs are short sequence tags, even modest sequencing or synthesis error can bias isoform quantification. Library strategies therefore differ in whether they use orthogonal short-read sequencing to assist CB and UMI identification or recover barcode information directly from long-read data.

Early scLR-seq workflows largely used short-read data to support cell and molecule assignment. In parallel designs such as ScISOr-Seq [31], ScNaUmi-seq [32], and FLT-Seq [33], barcoded full-length cDNA is divided between short-read sequencing, which provides high-confidence CB and UMI references and cell-state information, and long-read sequencing, which resolves transcript architecture. Related targeted designs, including LR-Split-seq [34] and RAGE-seq [35], use short-read profiles to guide long-read analysis towards selected cells or transcript classes. These strategies established a reliable route for linking isoform structures to cellular identity, but dual-platform sequencing increases cost, workflow complexity and integration burden.

More recent approaches recover CBs and UMIs directly from long-read data, reducing dependence on matched short-read sequencing. One route modifies barcode or molecule architecture for long-read compatibility. For example, scCOLOR-seq [36] uses redesigned CB and UMI sequences based on dimer nucleotide blocks, enabling error detection and correction in nanopore reads. Anchor-enhanced bead designs [37] insert defined sequences between barcode and UMI regions to improve boundary detection and reduce synthesis-associated errors. Molecular strategies such as R2C2 [38] generate concatemeric reads by rolling-circle amplification to yield higher-confidence consensus sequences. A second route retains standard barcoded cDNA formats but uses long-read-native demultiplexing. Tools including BLAZE [39], scNanoGPS [40], and Flexiplex [41] recover CBs and UMIs through whitelist correction, de novo clustering or error-tolerant matching. Their performance depends on barcode structure, read quality, expected cell number and the availability of suitable whitelists, which are discussed further in the Computational Methods section.

Recent benchmarking indicates that short-read-free approaches can approach the accuracy of short-read-assisted methods while reducing experimental complexity and cost [42]. Their performance should nevertheless be evaluated for the library architecture, sequencing chemistry and biological endpoint of each study.

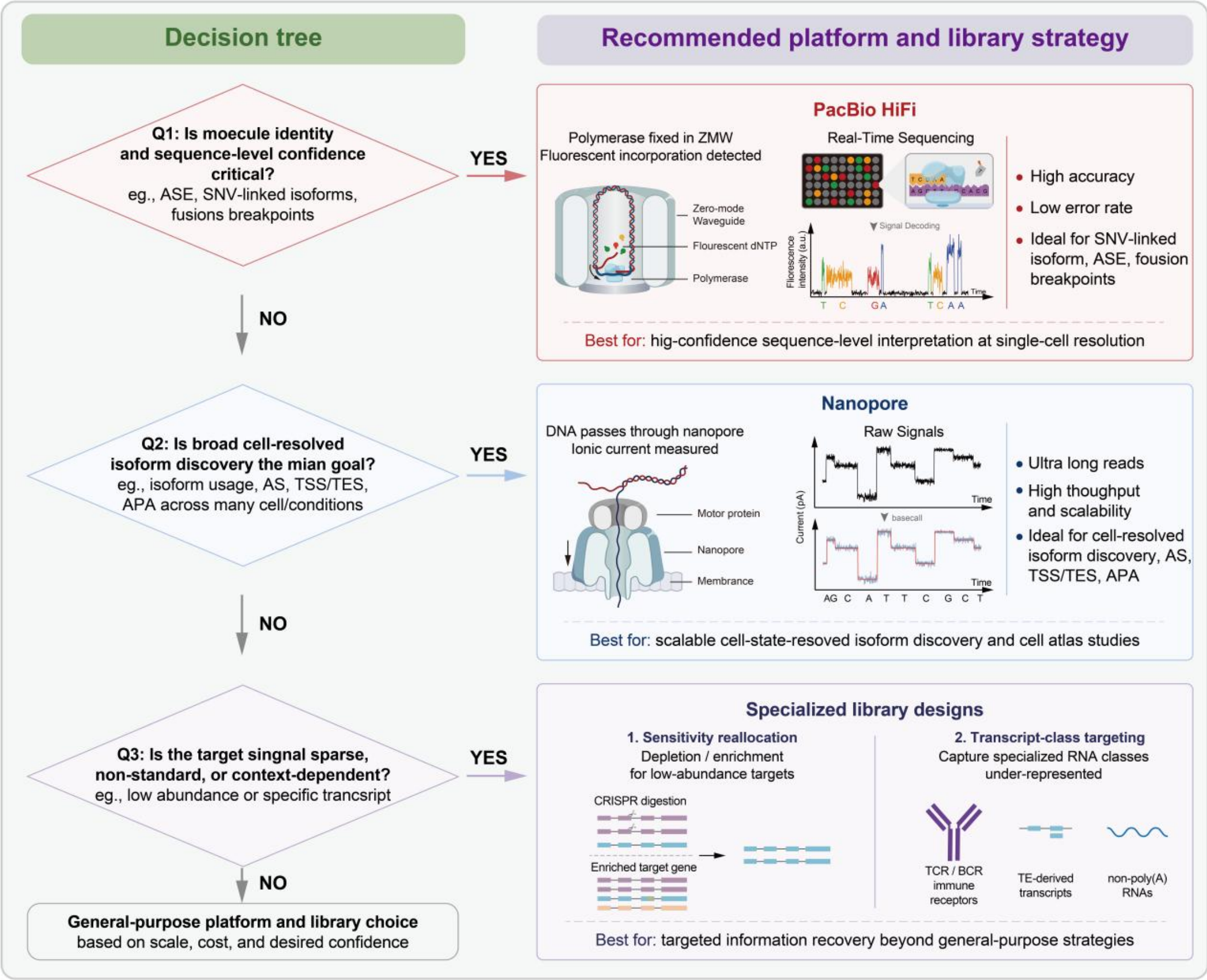

Figure 2. Decision framework for selecting sequencing platforms and library strategies in single-cell long-read transcriptomics. The decision tree guides experimental design according to the primary biological objective. PacBio HiFi is recommended when accurate molecule identification and high-confidence sequence-level interpretation are required. Nanopore sequencing is better suited for scalable, cell-resolved discovery of isoform usage, alternative splicing, transcription start and end sites, and alternative polyadenylation across large numbers of cells or conditions. When the target signal is sparse, non-standard, or context dependent, specialized library designs can improve information recovery through depletion or enrichment of low-abundance targets or through selective capture of transcript classes such as immune receptor transcripts, transposable element-derived transcripts, and non-polyadenylated RNAs. For studies without a dominant specialized requirement, platform and library selection should be guided by experimental scale, cost, throughput, and the required level of sequence confidence.

Table 1. Core library construction strategies

Method	Concept	Advantages	Limitations
Short-read-assisted strategies			
ScISOr-Seq [31]	PacBio full-length sequencing of barcoded cDNA from droplet-based scRNA	High HiFi accuracy; enables cell-type-specific isoform discovery	Lower throughput; needs dedicated processing
ScNaUmi-seq [32]	Split-library: short-read barcodes guide nanopore full-length reads	Accurate molecule assignment; concordant with short-read data	Requires paired short-read sequencing and dual-platform workflow
FLT-Seq [33]	Split-library 10x sequencing with short-read-guided barcode assignment and clustering	Enhances detection of low-abundance isoforms while retaining full-cell coverage	Still requires dual-platform sequencing and split library
LR-Split-seq [34]	Short-read-guided cell-state definition with long-read sequencing of selected cells	Reduces cost by sequencing a subset of cells; suitable for following up existing atlases	Long reads cover only subset of cells; rare isoforms may be missed
RAGE-Seq [35]	Targeted long-read immune receptor sequencing linked to droplet-based scRNA-seq	High-throughput clonotype-resolved profiling with matched transcriptomes	Focuses on immune receptors; not transcriptome-wide
Short-read-free strategies			
scCOLOR-seq [36]	Bi-nucleotide barcodes enable error detection and direct ONT correction	Short-read-free; supports isoform, fusion and transcript quantification	Requires custom barcoded beads and prior barcode knowledge
Anchor-Enhanced Bead Design [37]	Anchor-guided barcode/UMI design to reduce truncation errors	Improves UMI recovery and transcript detection at library design level	Requires custom bead design; mainly tackles synthesis rather than sequencing errors
R2C2 [38]	Rolling-circle amplification with consensus sequencing for ONT error correction	Improves accuracy through consensus; cost-effective full-length profiling	May introduce amplification bias; throughput tied to circle generation efficiency
scLIS-seq [43]	Plate-based long-read isoform sequencing using Smart-seq3xpress-derived amplification	High sensitivity for low-abundance isoforms and complex transcript detection	Low throughput; dependent on FACS/manual cell isolation; ONT errors need correction
MAS-seq [44]	Concatenates multiple cDNAs into long molecules to boost PacBio throughput	Greatly increases throughput; integrates with 10x workflows	Needs extra concatemer construction; may limit very long transcripts
SCAN-seq [45]	RT primer integrates cell barcodes for pooled nanopore sequencing of full-length cDNA	Enables alternative splicing, novel transcript and allele-specific analyses without short reads	Limited throughput and labor intensive; ONT errors still need correction
SCAN-seq2 [46]	Combinatorial 3′ and 5′ barcoding for higher-throughput full-length single-cell sequencing	Improves throughput and supports isoform quantification and immune-receptor analysis	Complex library preparation and demultiplexing

Specialized and Application-Oriented Library Designs

Specialized library designs have extended the range of biological questions addressable by scLR-seq. By incorporating targeted enrichment or molecular depletion into standard workflows, these strategies focus sequencing capacity on transcript features that would otherwise be poorly recovered.

One approach is to redistribute sequencing capacity towards transcripts that are under-represented in conventional single-cell libraries. CRISPR-based depletion strategies such as scCLEAN [47] remove highly abundant non-target transcripts with a CRISPR-Cas9 system before sequencing, increasing effective coverage of lower-abundance transcripts and rare isoforms. Conversely, targeted long-read methods such as scRaCH-seq [48] enrich predefined transcripts from existing single-cell cDNA libraries, enabling focused analysis of selected genes, splice variants or mutation-bearing transcripts.

Other library designs address transcript classes that require full-length or locus-resolved information. RAGE-seq [35] captures full-length T cell receptor and B cell receptor sequences, retaining complete V(D)J recombination information and enabling immune repertoire features to be linked with cellular identity. SMART-Seq-Total [49] extends profiling beyond poly(A)-selected mRNA by using Escherichia coli poly(A) polymerase to tail diverse RNA species, enabling full-length analysis of polyadenylated and non-polyadenylated transcripts, including miRNAs, snoRNAs and histone RNAs.

Collectively, available evidence suggests that library selection should be guided by the information recovered per cell rather than platform identity alone [50]. Standard 10x-derived cDNA libraries are compatible with established droplet-based workflows, but isoform analysis may be limited by cDNA length bias, incomplete recovery of long transcripts, barcode and UMI retention, and isoform-level filtering. Targeted depletion or enrichment can alter library information content by removing highly abundant transcripts or increasing coverage of selected molecules. Library design should therefore be evaluated against cell throughput, molecule recovery, transcript completeness, isoform sensitivity and the intended endpoint, whether broad atlas construction, deep isoform analysis, immune receptor reconstruction or targeted detection of rare features.

Table 2. Specialized strategies for single-cell long-read library preparation

Method	Library	Key Features	Outputs	Limitations
scCLEAN [47]	10x Chromium 3′ cDNA library with CRISPR-mediated depletion	CRISPR depletion of abundant transcripts; boosts rare isoform detection	Low-abundance transcripts, rare isoforms, difficult-to-detect transcript classes	Extra CRISPR step required
scRaCH-seq [48]	10x Chromium cDNA library (3′/5′ compatible)	Probe-based targeted long-read resequencing from 10x cDNA	Full-length targeted isoforms, point mutations, and splicing variants	Restricted to targeted genes; requires probe-panel design; enrichment may introduce bias
SMART-Seq-Total [49]	Plate-based full-length total-RNA cDNA library (96/384-well; FACS-sorted cells)	Full-length total-RNA capture with UMI; broad RNA biotype coverage	mRNA, lncRNA, miRNA, snoRNA, snRNA, tRNA, histone RNA, and related RNA biotypes	rRNA depletion is often required; limited throughput; complex workflow
CRISPR-edited Single-cell profiling [51]	Plate-based single-cell cDNA library after CRISPR perturbation	Single-cell long-read readout of CRISPR-induced isoform changes	Edited loci, transcript-structure consequences, and full-length edited isoforms	Target-focused; requires customized perturbation design and dedicated analysis
FlsnRNA-seq [52]	Protoplasting-free single-nucleus cDNA library	Protoplasting-free full-length single-nucleus RNA profiling in plants using isolated nuclei	Nuclear full-length transcripts and isoforms in plants	Plant-specific and nucleus-focused

Computational Methods for Data Processing

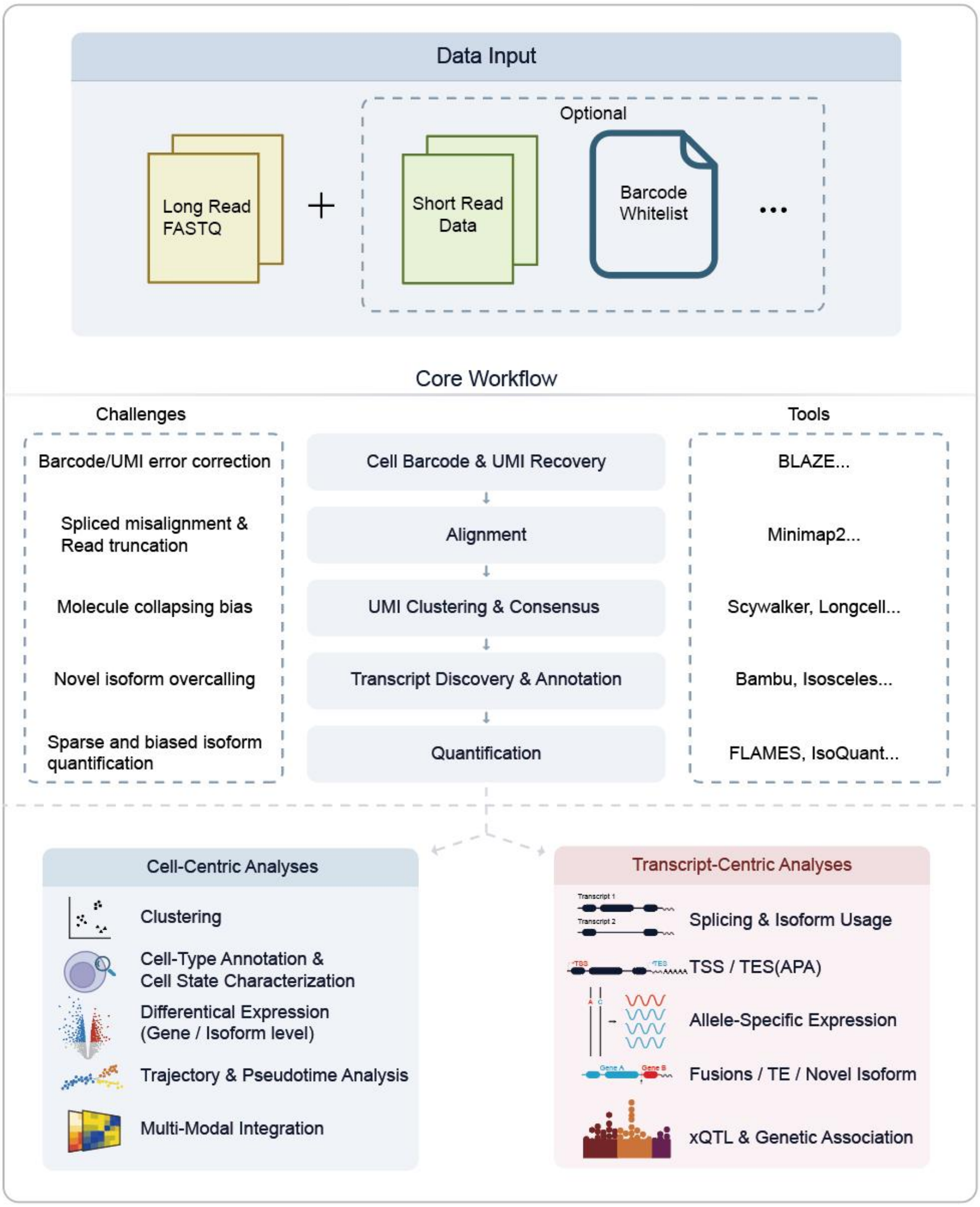

Figure 3. Overview of single-cell long-read RNA sequencing analytical workflow. The core workflow addresses common challenges in single-cell long-read transcriptomics, including barcode/UMI errors, spliced misalignment, read truncation, molecule collapsing bias, novel isoform overcalling, and sparse or biased isoform quantification. Key computational steps include cell barcode and UMI recovery, read alignment, UMI clustering and consensus building, transcript discovery and annotation, and isoform-level quantification. Downstream analyses are divided into cell-centric and transcript-centric approaches. Cell-centric analyses include clustering, cell-type annotation and characterization, differential expression, trajectory and pseudotime inference, and multi-modal data integration. Transcript-centric analyses focus on splicing and isoform usage, transcription start/end site (TSS/TES) analysis, allele-specific expression, detection of fusion or

novel transcripts, and integration with quantitative trait loci (QTL) and genetic association studies.

Cell Barcode and UMI Demultiplexing

In scLR-seq, accurate demultiplexing of CBs and UMIs is required for single-cell-resolved quantification. Although nanopore accuracy has improved markedly from approximately 95% with earlier R9 chemistries to over 99% with recent R10.4.1 chemistry, a typical 10 to 20 base pair barcode still carries an estimated 10-18% probability of containing at least one error. Such errors can cause misassigned cell identities or biased molecular quantification, making barcode recovery a central computational bottleneck and driving the development of both short-read-assisted and short-read-free demultiplexing strategies [30].

Demultiplexing strategies can be grouped by the source of barcode evidence. Short-read-assisted pipelines such as SiCeLoRe [32] use matched short-read data to provide high-confidence CB and UMI references for assigning long reads. Although reliable, this strategy entails additional cost, experimental complexity and cross-platform integration [13, 14]. Short-read-free workflows infer CBs and UMIs directly from long reads. Whitelist-based methods, including BLAZE [39], wf-single-cell [53] and PacBio scIso-Seq, are suited to standard barcode systems such as 10x-derived libraries, in which candidate barcodes can be corrected against predefined references. BLAZE, for example, was developed to identify 10x cell barcodes directly from nanopore scRNA-seq reads and showed strong concordance with matched short-read benchmarks.

De novo demultiplexing methods address cases in which barcode structures are unknown, customized or poorly matched to existing whitelists. scNanoGPS [40] uses de novo barcode clustering for same-cell genotype and phenotype analysis without short-read or whitelist guidance, whereas Flexiplex [41] supports error-tolerant matching for non-standard barcode designs. These approaches broaden the range of compatible library formats but are more sensitive to parameter settings, expected cell number, read quality and barcode abundance. Method selection should therefore reflect barcode architecture and data quality: matched short-read references maximize assignment confidence, whitelist-based long-read methods offer practical scalability for standard libraries, and de novo approaches provide flexibility with greater benchmarking requirements.

Benchmarks of long-read scRNA-seq demultiplexing show substantial differences in CB recovery and UMI correction [42]. Across evaluated methods, approximately 92% of true CBs identified in matched short-read references were recalled. scNanoGPS, SiCeLoRe (v2.1) and Bambu additionally reported hundreds to thousands of candidate CBs beyond the reference set, suggesting increased sensitivity but potentially more false positives. BLAZE-FLAMES and wf-single-cell achieved favorable precision-recall trade-offs, whereas SiCeLoRe tended towards high recall with lower precision, potentially owing to edit-distance-based merging. UMI correction also depended on sequencing chemistry: in simulations using R9.4.1 chemistry, wf-single-cell showed high F1 scores and adjusted Rand indices, and increased accuracy with R10.4.1 improved all evaluated tools. Collectively, benchmarking studies across different barcode recovery tools consistently indicate that sequencing chemistry and barcode architecture exert a stronger influence on recovery accuracy than algorithmic differences alone, highlighting experimental design as a primary determinant of demultiplexing performance.

Table 3. Cell Barcode and UMI Demultiplexing Methods

Method	Short-read -free	Whitelist needed	Primary task	Corrected element(s)	Correction strategy	Key feature
SiCeLoRe [32]	×	Yes	CB/UMI assignment; molecule consensus	CB + UMI	Short-read truth-set assignment + UMI-guided consensus	Short-read-guided CB/UMI assignment with molecule consensus
BLAZE [39]	✓	Yes	CB calling; UMI extraction	CB + UMI	Adapter/poly(T) localization + whitelist filtering + count cutoff	Count/quality-based 10x barcode calling from ONT reads
wf-single-cell [53]	✓	Yes	CB/UMI demultiplexing; count matrix generation	CB + UMI	Sockeye-based barcode calling + whitelist-guided correction	Official ONT Nextflow workflow built around Sockeye
scNanoGPS [40]	✓	No	CB calling; read assignment; optional variant analysis	CB + UMI	De novo CB calling + locus-aware UMI collapsing	iCARLO-based de novo cell calling without short reads or whitelist
Flexiplex [41]	✓	Optional	CB/UMI extraction; demultiplexing; barcode discoverv	CB + UMI	Flanking-sequence search + Levenshtein matching / discovery mode	Levenshtein-based flexible demultiplexing
scIso-Seq	✓	Yes	Barcode calling; UMI deduplication	CB + UMI	LSH-assisted whitelist rescue + edit-distance correction + groupdedup	Official PacBio HiFi single-cell workflow
scTagger [54]	×	Yes	LR–SR barcode matching	CB	Trie-based approximate matching to short-read CBs	Fast approximate matching between long- and short-read CBs
Longcell [55]	✓	Yes	CB/UMI recovery; isoform quantification	CB + UMI	k-mer barcode alignment + edit-distance filtering + UMI denoising	UMI-aware denoising for isoform quantification
ScNapBar [56]	×	Yes	Barcode assignment	CB	UMI matching or Naïve Bayes with Illumina-derived priors	Hybrid UMI/Bayesian barcode assignment
FLAMES [33]	✓	Optional	CB/UMI assignment; isoform quantification	CB + UMI	External CB calling + edit-distance UMI collapse	End-to-end isoform workflow; supports external CB callers
Scnanoseq [57]	✓	Yes	Barcode correction; UMI deduplication; quantification	CB + UMI	BLAZE-based calling + barcode correction + deduplication	Reproducible nf-core end-to-end ONT scRNA-seq pipeline

Transcript Discovery and Quantification

After CB and UMI assignment, transcript discovery and quantification determine how effectively scLR-seq resolves isoform diversity and cell-type-specific splicing. Currently, nanopore methodologies often suffer from low validation rates, where only 50-70% of raw reads receive accurate barcode assignments. The resulting depth of several thousand to ten thousand reads per cell fails to match short read standards, restricting the profiling of low abundance transcripts [50]. Crucially, systematic analysis shows that these deficiencies stem largely from truncated isoforms in the assembly, which manifest as a distinct three prime bias and five prime truncation in the sequence data [58]. These shortened reads are widely attributed to underlying challenges such as homopolymer regions, premature reverse transcription termination, or fluctuating flow cell performance including low enzyme processivity and voltage instability [59-61]. Consequently, compensating for these technical biases and truncated reads has become a driving force behind the development of specialized transcript discovery and quantification workflows.

Although long reads provide direct evidence of exon connectivity, limited per-cell depth makes transcript discovery vulnerable to sparse isoform support, splice-junction errors, read truncation and imperfect molecule collapsing. These factors can fragment transcript models or inflate novel isoform calls. Annotation-guided tools such as Bambu [62] and IsoQuant [63] combine reference transcripts with splice-aware models to separate supported transcript structures from alignment or sequencing noise, making them useful in well-annotated genomes. For PacBio data, Iso-Seq and scIso-Seq [64] use the accuracy of CCS/HiFi reads to support full-length transcript consensus generation. For sparse single-cell or spatial data, approaches such as Bambu-Clump [62, 65] can share information within cell clusters to support transcript discovery and quantification.

Isoform quantification is complicated by reads, particularly truncated reads, that are compatible with multiple transcripts. Methods such as Isosceles [66] use transcript compatibility counts and expectation-maximization inference for single-cell isoform quantification, with improved handling of ambiguous read assignment relative to earlier approaches such as ESPRESSO. In addition, to address the limited sequencing depth of single cells, recent methods such as LRAA [67] introduce cluster-guided strategies. By pooling information from cells with similar biological profiles, these methods can recover low-abundance transcripts that might otherwise be lost in the noise of a single cell. For poorly annotated genomes or non-model organisms, Mandalorion [68] provides strong de novo transcript reconstruction capabilities.

In practical research, end-to-end pipelines increasingly streamline these analytical steps. Tools such as FLAMES [33] integrate barcode correction, transcript assembly, and rapid quantification into a unified framework, enabling efficient processing from raw reads to expression matrices. Other frameworks, including TALON [69] and FLAIR2 [70], emphasize stringent filtering and validation of transcript models, and are commonly used in large-scale consortium projects to ensure reproducibility.

Benchmarking studies suggest that read length and accuracy can affect the reliability of transcript models more strongly than depth alone [12, 66, 71]. Reference-guided tools such as Bambu and IsoQuant generally perform well in well-annotated genomes, whereas tools such as StringTie2, FLAIR, and Mandalorion may be useful for exploratory novel-isoform discovery but require stringent validation to control false positives. These benchmarking results reflect a trade-off between discovery breadth and model specificity.

For quantification, sequencing depth and the strategy used to handle ambiguously assigned reads are important determinants of performance. Isosceles has shown strong performance in isoform-level quantification across single-cell, pseudo-bulk, and bulk sequencing settings, and is particularly suitable for addressing ambiguity in isoform abundance estimation. By contrast, IsoQuant and Bambu represent robust and broadly applicable robust default tools for reference-based transcript quantification. Benchmark comparisons of isoform quantification methods suggest that differences in read truncation handling and ambiguity resolution contribute more to variability in abundance estimates than the choice of alignment strategy, particularly in low-depth single-cell settings.

Overall, the central challenge in transcript discovery and quantification is to convert molecular evidence from full-length reads into interpretable cell-level isoform matrices without allowing sparse support, alignment noise or ambiguous assignment to destabilize transcript catalogues and abundance estimates. Future robust workflows should coordinate transcript reconstruction, filtering and quantification, and should report read depth, transcript support and filtering criteria clearly to support downstream interpretation and cross-platform comparison.

Table 4. Transcript Discovery and Quantification Methods

Method	Reference-guided novel transcript discovery	Annotation-free de novo discovery	Core principle	Strengths / recommended use
Bambu / bambu-clump [62, 65]	Yes	Limited	ML-guided reference-based discovery + context-aware quantification	Conservative, high-confidence discovery in annotated or shallow sc/spatial data
IsoQuant [63]	Yes	Yes	Intron-graph reconstruction + annotation-assisted correction	Balanced sensitivity/precision; suitable for ONT and PacBio
Iso-Seq [64]	Optional	Yes	HiFi processing + clustering/deduplication + isoform classification	Standard PacBio workflow for full-length isoform discovery
Isosceles [66]	Yes	Yes	DAG splice graph + TCC/EM quantification	Sensitive detection and accurate quantification, especially for sparse data
ESPRESSO [72]	Yes	Yes	Error-aware splice-graph reconstruction + abundance estimation	Useful when splice correction and quantification are both priorities
LRAA [67]	Yes	Yes	Strand-specific splice-graph assembly + EM quantification	Flexible exploratory analysis across bulk, sc, and snRNA data
Mandalorion [68]	Optional	Yes	Consensus-based isoform calling from accurate reads	Strong for poorly annotated or non-model systems
FLAMES [33]	Yes	Limited	Barcode-aware semi-supervised isoform calling	Practical ONT workflow for isoforms, DTU, and mutation-aware analysis
FLAIR / FLAIR2 [70, 73]	Yes	Yes	Alignment correction + isoform collapse; variant-aware in FLAIR2	High-confidence isoform filtering and variant-/haplotype-aware analysis
SCOTCH [68]	Yes	Yes	Sub-exon modeling + novel-read clustering	Strong for isoform usage and DTU in barcoded single-cell long-read data
StringTie2 [74]	Yes	Yes	Network-flow transcript assembly	Fast general-purpose baseline for annotated genomes
TALON [69]	Yes	Limited	Database-based transcript tracking	Conservative multi-sample reproducibility tracking
Oarfish [75]	No	No	Probabilistic long-read quantification	Fast, memory-efficient complement to discovery tools
lr-kallisto [76]	No	No	Long-read pseudoalignment + EM quantification	Fast, low-memory quantification for large datasets
LongcellPre [55]	No	No	CB/UMI recovery + consensus correction	Improves ONT quantification for downstream Longcell analysis
scNanoGPS [40]	No	No	iCARLO deconvolution + consensus BAM generation	Best for joint isoform–mutation and genotype–phenotype analysis

Cell Type Annotation

Cell type annotation is a prerequisite for downstream biological interpretation and aims to assign each cell a defined identity based on its transcriptomic profile. Established approaches include marker-gene-based methods, such as SCINA [77] and scCATCH [78], reference-mapping methods, such as SingleR [79] and clustifyr [80], and machine-learning classifiers. These approaches have been extensively applied to short-read scRNA-seq data and provide a mature

gene-expression-based framework.

Applying these frameworks to long-read data exposes an important limitation in that most current annotation methods operate at the gene-expression level and were designed for short-read technologies. scLR-seq additionally captures structural features, including alternative splicing and variation in transcription start and end sites, that can contain cell-type-specific information not apparent from gene-level expression [31, 81, 82]. Relying only on gene counts may therefore obscure biologically relevant heterogeneity encoded by isoform usage.

Future developments are likely to move toward multi-layered annotation strategies. Advances in deep learning models, particularly in representation learning for transcript features, are expected to facilitate this transition. Recent work such as ICON, which employs random forest models, has applied isoform-level single-cell long-read data for cell type annotation [83]. Moreover, the development of isoform-based annotation depends on appropriate reference resources. Compiling validated catalogs of cell-type-specific isoforms and constructing rigorous benchmarks to test platform reliability represent the critical next steps required to fully anchor the development of isoform-based single-cell classification.

Biological Insights Enabled by Long-Read Sequencing

Table 5. Functional Analysis Tools for Single-Cell Long-Read Transcriptomics

Method	Compatibility with scLR data	Analysis module	Key strength	Example application
Alternative Splicing and Isoform Usage Analysis Tools				
Longcell [55]	Native	Isoform usage and alternative splicing analysis	UMI-aware isoform quantification with intra-/inter-cell splicing heterogeneity modeling; spatial-ready	Spatial isoform switching; splice-factor perturbation targets
IsoTools 2.0 [84]	Compatible	Transcript structure, TSS, and alternative splicing analysis	Comprehensive long-read transcriptome analysis with TSS prediction, entropy, and differential splicing	Long-read TSS prediction and AS analyses
Chevreul [85]	Compatible	Full-length single-cell transcript exploration and visualization	Exploratory analysis and visualization of full-length single-cell sequencing data	Full-length single-cell isoform and exon-coverage exploration
scCyclone [86]	Native	Differential isoform usage, differential splicing, and RBP analysis	Integrated DTU/DSE/RBP toolkit with rMAPS-compatible output	Public single-cell long-read demo datasets
IsoformSwitchAnalyzeR [87]	Compatible	Isoform switching and functional consequence analysis	Functional consequence analysis of isoform switches in long-read and single-cell data	Disease-associated isoform switching analyses
Transcription Start and End Site Analysis Methods				
scTSS [88]	Compatible	Transcription start site usage analysis	Alternative TSS detection and comparison from 5′ scRNA-seq data	Cell-type-specific TSS and promoter-usage analysis
scAPA [89]	No	Alternative polyadenylation analysis	APA site usage analysis from 3′-tag scRNA-seq	Cell-type APA dynamics and PAU shifts
ouro-seq [90]	Native	Full-length single-cell transcriptome profiling	Improves recovery of authentic full-length transcripts and enables comprehensive isoform-level analysis	Alternative splicing, alternative promoter usage, APA, and isoform landscape
Mutation & ASE Tools				
LongSom [91]	Native	Somatic variant, CNA, and fusion profiling	Joint calling of somatic SNVs, CNAs, and fusions from single-cell long-read RNA-seq	Ovarian-cancer clonal heterogeneity and prognostic subclones
ASPEN [92]	Compatible	Allele-specific expression analysis	Adaptive-shrinkage ASE modeling with improved sensitivity and dispersion control	F1 mouse brain organoids and T cells
Fusion Transcript Analysis Tools				
LongFUSE [93]	Native	Fusion transcript and fusion-isoform detection	Cell-level fusion and fusion-isoform calling with XOR-based candidate search	TMPRSS2–ERG fusion and fusion-positive tumor-cell identification

CTAT-LR-Fusion [94]	Native	Fusion transcript detection	Accurate long-read fusion calling with or without matched short reads	Fusion detection in bulk and single-cell cancer transcriptomes
Transposable Elements Analysis Tools				
scTE [95]	Compatible	Transposable element expression analysis	Family-level TE quantification for scRNA-seq	Cell-fate-specific TE dynamics in development and disease
SoloTE [96]	Compatible	Locus-specific transposable element expression analysis	Locus-aware TE quantification for scRNA-seq	Two-cell embryo and disease TE activity analyses
CELLO-seq [97]	Native	Locus-specific transposable element and TE-chimeric transcript analysis	Long-UMI long-read framework for locus-specific young-TE and TE-chimeric transcript detection	Active versus readthrough TE transcription in single cells
RNA Modification Analysis Methods				
m6A-isoSC-seq [98]	Native	m6A-aware isoform profiling	Captures cell- and isoform-level m6A heterogeneity	Isoform-level m6A and modification-associated transcript states
Multi-Omics Methods				
scNanoCOOL-seq [99]	Native	Long-read single-cell multi-omics	Integrates transcriptome and epigenome in the same cell	Allele-specific regulation and complex genomic regions
SPLONGGET [100]	Native	Genome–epigenome–transcriptome profiling	Connects genetic variants with chromatin and transcript output	SVs, CNAs, full-length transcripts, and splice-affecting variants
ScISOr-ATAC [101]	Native	Full-length RNA + ATAC profiling	Links chromatin accessibility with splicing in the same nucleus	Chromatin–splicing coupling in frozen tissues
scGTP-seq [102]	Compatible	Genome–transcriptome co-profiling	Resolves structural variation with transcriptomic effects	ecDNA and SV-associated expression changes

Alternative Splicing Dynamics and Isoform Usage

Alternative splicing and isoform usage regulate gene output by generating transcripts with distinct coding or regulatory features [103]. Changes in isoform composition can therefore alter protein products, untranslated regions, or RNA fate, even when total gene abundance changes little.

Short-read approaches have established mature frameworks for analyzing differential splicing and transcript usage. However, they often infer isoform structure from local junctions or exon signals. This limitation makes it difficult to determine whether multiple splicing events occur within the same transcript molecule. Long-read data help close this gap by directly observing full-length isoforms, but sparse per-cell coverage and ambiguous read assignment still complicate differential isoform and splicing analysis.

Computational tools for scLR-seq isoform analysis now span discovery, comparison,

visualization, and interpretation. LongCell highlights intra- and inter-cellular isoform diversity, while Scywalker and IsoTools 2.0 connect transcript discovery with differential usage and local splicing analysis [55, 84, 104]. LRAA and Chevreul improve the recovery and inspection of complex isoforms [67, 85]. Recent frameworks, including scCyclone and IsoformSwitchAnalyzeR v2, extend isoform analysis toward regulatory and functional interpretation by linking DTU, DSE, RBP motif enrichment, or predicted consequences of isoform switching [86, 87].

Despite methodological progress, splicing and isoform analysis remain limited by sparse per-cell coverage and ambiguous read assignments. Current strategies therefore integrate discovery, quantification, and functional interpretation rather than focusing on a single output. More standardized statistical frameworks and benchmarks are still needed to improve the reproducibility of differential isoform and splicing analyses across platforms.

Transcription Start and End Site Resolution

Transcription start site (TSS) selection and transcription end site (TES) usage are critical regulatory layers that define transcript boundaries and functional potential. Alternative TSS usage can reshape the 5′ UTR and influence translation initiation, whereas alternative TES selection can modulate 3′ UTR length. These changes may affect RNA stability, localization, and translational efficiency [105, 106].

A key limitation of short-read and tag-based single-cell approaches is that transcript boundaries are often measured separately from the complete isoform body. 5′-focused data can support promoter or TSS analysis, and 3'-focused data can inform poly(A) site usage. However, these signals are usually insufficient to assign boundary usage to complete transcript isoforms. This creates a gap between detecting boundary usage and resolving boundary-defined isoforms, especially when transcripts from the same gene share most internal exons but differ at their 5′ or 3' ends.

Several recent tools begin to address these challenges from complementary perspectives. scTSS uses 5′ single-cell RNA-seq data to identify TSS clusters, quantify their usage, and test differential TSS usage across cell groups or conditions [88]. In long-read transcriptome analysis, IsoTools 2.0 incorporates TSS information into reconstructed transcript models, helping distinguish promoter-associated isoforms within the same gene locus [84]. For 3' end regulation, APA analysis captures shifts in poly(A) site usage that generate alternative terminal isoforms or alter 3′ UTR length. Ouro-Seq shows that alternative promoter usage, APA, and splicing can be jointly examined in single-cell long-read data, providing a more connected view of transcript-boundary regulation [90].

Future progress will depend on more reliable discrimination of true transcript ends from truncation and priming artefacts, statistical models for differential boundary usage, and benchmarks that evaluate end-site resolution across library chemistries. Integrating boundary-defined isoforms with RBP binding, miRNA regulation, genetic variants, and functional annotation may further clarify when altered TSS or TES usage has regulatory consequences rather than reflecting passive transcript diversity.

Somatic Mutations and Allele-Specific Expression

Somatic mutations and allele-specific expression (ASE) provide a genetic perspective on single-cell transcriptomes. Somatic mutations capture acquired genetic differences among cells and offer insight into clonal evolution, whereas ASE reveals asymmetric transcriptional activity

between the two allelic copies of a gene. These signals are particularly informative when genetic variation, clonal structure, and transcriptional output need to be interpreted together [107, 108].

A major limitation of short-read single-cell RNA-seq is that variant information is sparse and fragmented. Somatic mutation detection often relies on weak support at individual sites, making true mutations difficult to distinguish from sequencing errors and background polymorphisms. ASE estimates often rely on only a few heterozygous SNPs, making them sensitive to low coverage, mapping bias, and overdispersion. Long-read sequencing expands the available variant information by covering more SNP sites within individual transcripts. This increased density of informative loci provides a richer set of candidate variants for both somatic mutation detection and ASE analysis, narrowing the gap between variant detection and transcript-level interpretation [16, 91, 107].

Computational methods in this area are beginning to separate into somatic-variant and allele-specific frameworks. LongSom represents the former direction, using long-read single-cell RNA-seq to detect somatic SNVs, mitochondrial variants, copy number alterations, and fusion transcripts for clonal reconstruction [91]. For ASE, earlier single-cell approaches mainly addressed sparse allelic counts and overdispersion. ASPEN, for example, uses a moderated beta-binomial model with adaptive shrinkage to stabilize ASE estimation [92]. LongAllele further adapts allele-specific analysis to long-read bulk and single-cell RNA-seq by jointly inferring heterozygous variants, haplotype structure, read-haplotype assignment, and ASE, thereby making fuller use of the multi-variant information carried by individual long reads [109].

Somatic mutation and ASE analysis extend scLR-seq from transcript discovery to genetically informed transcriptomics. Future work should improve error-aware variant calling, haplotype phasing, and allele-specific isoform quantification, especially for lowly expressed genes and rare cell populations. Matched DNA sequencing, benchmark datasets, and models that jointly evaluate allelic imbalance with transcript structure will be important for distinguishing robust regulatory signals from technical noise.

RNA Modifications

RNA modifications add a regulatory layer to transcriptome biology, and N^6-methyladenosine (m^6A) is the most abundant internal modification in eukaryotic mRNA. This modification regulates multiple biological processes, including transcript stability, nuclear export, and degradation. Crucially, m6A sites often exhibit substantial cell to cell variability in both their presence and stoichiometric abundance, meaning that these epitranscriptomic profiles can effectively distinguish distinct cell subpopulations.

Most current methods for detecting m6A require substantial RNA input and are applied to bulk samples. Such measurements average modification signals across cells and can obscure heterogeneity in complex tissues or disease contexts. Moreover, while m6A site enrichment near stop codons requires extensive transcript coverage, simultaneous recovery of modifications and cell identities remains constrained by current sequencing chemistry. Single-cell platforms typically rely on cDNA synthesis to introduce cell barcodes, which removes or obscures native m6A signals. Because alternative direct RNA sequencing technologies cannot readily incorporate these cell-associated barcodes, concurrent quantification of both features in individual cells remains technically challenging.

One current single-cell strategy for m^6A detection converts modified-base information into sequence changes that can be detected during sequencing. For example, m^6A-isoSC-seq [98] uses

APOBEC1-YTH to induce C-to-U mutations near m^6A sites and combines 10x Genomics single-cell cDNA libraries with ONT long-read sequencing to detect isoform-level m6A in individual cells. Single-cell long-read sequencing enables m6A heterogeneity analysis but remains constrained by cDNA dependence, sensitivity, and throughput. Improvements in direct RNA sequencing, labelling and barcode retention will be needed to measure transcript structure and RNA modification together in single cells.

Genetic Regulation at Transcript Resolution

Genetic variants can regulate transcript choice as well as transcript abundance. A variant may shift isoform usage, alter splice junction selection, or affect promoter and polyadenylation site usage while producing only modest changes in total gene expression. Transcript-resolution QTL analysis is therefore important for identifying the specific RNA products through which regulatory variants act.

Conventional eQTL analysis mainly links variants to total gene expression, whereas short-read sQTL or trQTL analyses often rely on local junctions or incomplete transcript models. This creates a gap between detecting a regulatory association and identifying the full-length isoform affected by that association. The limitation is especially relevant for unannotated, low-abundance, or cell-type-specific isoforms, which may be missed or collapsed in gene-level and short-read-based analyses.

For scLR-seq, the key methodological advance is the construction of a unified QTL framework based on full-length transcript phenotypes rather than a single QTL category. Long reads can define isoform abundance, isoform usage, splice-junction patterns, TSS usage, and TES/APA usage within the same transcript model and cellular context. These phenotypes can then be tested separately or jointly to determine whether a variant primarily affects expression level, splicing, transcript choice, or transcript boundaries. Recent single-cell long-read isoQTL studies illustrate this direction by combining sparse-count modelling, cell-type-specific effect estimation, and GWAS integration to connect regulatory variants with full-length transcript structures in relevant cell populations [110].

Future work should move beyond isoQTL discovery toward disease-oriented interpretation. GWAS–QTL integration methods, including SMR and colocalization, can help assess whether disease associations and transcript-level QTLs reflect the same regulatory mechanism. Combining isoQTL with other molecular QTL layers may further prioritize the cell types, regulatory pathways, and effector isoforms most directly affected by disease-associated variants.

Complex Transcripts: Fusions, Transposable Elements, and Novel Isoforms

Complex transcripts, including gene fusion transcripts [94, 111], transposable element (TE)-derived transcripts [112], and unannotated isoforms [16], are not merely artifacts. They can reflect important layers of transcriptome regulation. Fusion transcripts can produce novel chimeric RNAs or proteins that contribute to oncogenic processes and serve as clinically relevant biomarkers, particularly in cancer. Gene regulatory sequences carried by transposable elements can be exapted as promoters, enhancers, or non-coding RNAs that influence host gene expression and regulatory evolution. Novel isoforms expand the functional repertoire of a single gene, enabling differential protein functions and regulatory roles across cell types and states. Together, these complex transcripts represent functional diversity that shapes cellular identity, regulatory programs, and evolutionary innovation [9, 20].

Complex transcriptome features are biologically meaningful but difficult to resolve with conventional short-read sequencing. Because individual short reads rarely cover entire transcripts or junctions, full fusion isoforms can be ambiguous to reconstruct. Assignment of sequences derived from repetitive regions such as TEs is also uncertain, and novel isoforms can be difficult to distinguish from fragments of similar annotated transcripts. Long-read sequencing mitigates these issues by capturing full-length transcript sequences, reducing reliance on computational assembly and improving the accuracy of identifying and quantifying complex structures.

Several long-read-oriented tools have been developed to exploit the ability of long reads to span complete transcript structures and complex junctions. For fusion transcripts, LongFUSE [93] and CTAT-LR-fusion [94] use long-range information to detect and quantify complete fusion isoforms at single-cell resolution, improving sensitivity and structural resolution relative to short-read approaches. For TE-derived transcripts, scTE [95] and SoloTE [96] extend single-cell analysis to TE expression by summarizing multi-mapping reads at the family level or assigning locus-specific activity. Long-read methods such as CELLO-seq [97] use long UMIs and error correction to measure full-length TE-derived molecules reliably, enabling locus-specific and cell-type-specific characterization. Large long-read single-cell atlases, including TRAILS [113] and Ouro-Seq [90], have revealed that many unannotated transcripts are widespread and biologically meaningful, reflecting cell-type-specific splicing and polyadenylation programs.

Future progress will require more robust modelling of multi-mapping, low-abundance isoforms, and technical noise. As long-read platforms increase throughput and accuracy, these strategies should make complex transcript features more tractable as phenotypic markers and mechanistic signals.

Multi-omics integration

Single-cell multi-omics measures complementary molecular layers within individual cells and can link cellular identity to regulatory state. It not only enables the identification of cell types and rare subpopulations, but also reveals regulatory relationships across the genome, epigenome, transcriptome, and even post-translational levels, which is critical for understanding cell heterogeneity, developmental trajectories, and disease mechanisms. scLR-seq contributes full-length transcript structures and isoform usage, providing a transcript-resolved layer that can be integrated with genomic, epigenomic and spatial measurements. Recent spatial long-read studies have demonstrated that splicing and polyadenylation regulation have clear tissue-spatial dimensions [42, 114].

Currently, long-read single-cell multi-omics remains limited by the lack of mature library preparation protocols and standardized computational pipelines capable of simultaneously capturing full-length transcriptome and epigenome information. In addition, there is a shortage of dedicated analytical frameworks for jointly interpreting transcript expression and chromatin accessibility. Recent multi-omics methods, including scNanoCOOL-seq [99], SPLONGGET [100], and ScISOr-ATAC [101], address these issues by implementing customized library designs that enable simultaneous profiling of chromatin accessibility and full-length transcripts within the same cell. Their associated analytical frameworks enable joint assessment of isoform usage, promoter activity and chromatin state, but broader benchmarking is needed to establish reproducibility and scalability.

By linking transcript structure with epigenomic context, single-cell long-read multi-omics may improve the interpretation of cellular heterogeneity and regulatory function. Its widespread

use will require simpler experimental protocols and validated integration frameworks that perform robustly at larger scale.

Applications in Immunology and Beyond

scLR-seq captures transcript-processing programs that shape cellular states, features that are often missed by gene-level expression. It helps connect isoform diversity with immune identity, developmental organization, genetic regulation, and disease-associated cellular remodeling, which is helpful in immunology, neuroscience, and oncology[115].

In immune systems, scLR-seq has revealed that immune-subset identity and functional states are accompanied by cell-type-specific isoform programs. The TRAILS atlas, which maps full-length transcripts across 29 peripheral blood immune cell subsets by long-read sequencing, shows widespread unannotated isoforms with clear cell-type-specific patterns. Alternative 3′ UTR usage contributes to shaping these specific expression profiles. Integrating isoform switch analysis with QTL and GWAS data further highlights isoforms linked to immune-related diseases [113]. Another long-read single-cell study of human PBMCs uncovered numerous novel, cell-type-specific isoforms, revealing isoform diversity within functional immune contexts [116]. Full-length immune-receptor analyses have further shown that TCR and BCR clonotypes are coupled with transcriptional states, allowing clonal relationships to be linked with activation programs and functional differentiation at single-cell resolution [35]. More recently, the integration of scLR-seq with immune-repertoire profiling has revealed coordinated changes in isoform usage and immune receptor clonotypes during immunosenescence, suggesting that aging-related immune remodeling involves transcript-structure regulation in addition to conventional gene-level expression changes [117].

In the nervous system, single-cell long-read transcriptomics has revealed that brain cell identity and maturation are accompanied by extensive transcript-structure remodeling. Joglekar et al. showed that full-length isoform usage varies across brain regions, cell subtypes, developmental stages, and species, with cell-type-specific regulation of splicing, transcription start sites, and polyadenylation sites contributing to neuronal and glial diversity [118]. Extending this view to the developing human neocortex, Patowary et al. identified widespread developmental isoform diversity and isoform switches during cortical neurogenesis, many of which were predicted to alter RNA regulatory elements or protein structures; this isoform-resolved annotation also enabled neuropsychiatric risk variants to be interpreted within more cell- and stage-specific transcript contexts [119]. Spatial long-read analysis further showed that transcript-structure regulation is cell-type dependent and spatially organized. Foord et al. revealed developmentally regulated splicing and polyadenylation-site usage across distinct cortical layers and cell types, thereby linking isoform regulation to the anatomical organization of the cortex [114]. In disease contexts, Belchikov et al. further showed that frontotemporal dementia is associated with both cell-type-specific and broader splicing dysregulation in the human brain, with a substantial fraction of disease-associated splicing changes being masked by other cell types or cortical layers in mixed-cell analyses [120].

Cancer applications of scLR-seq increasingly focus on how transcript-structure variation contributes to tumor heterogeneity. In ovarian cancer, Dondi et al. showed that high-throughput full-length single-cell RNA sequencing of clinical metastatic samples captured extensive isoform diversity, including more than 52,000 previously unreported isoforms, and linked

cell-type-specific isoform usage with polyadenylation patterns, fusion transcripts, and expressed mutations within the same tumor ecosystem [121]. Extending this genotype–phenotype perspective, Penter et al. demonstrated that long-read sequencing can recover cancer- and immune-cell molecular barcodes, including SNVs, fusion genes, isoforms, CAR sequences, and TCRs, thereby enabling integrative tracking of tumor and immune phenotypes at single-cell resolution [122]. In colorectal cancer, Lu et al. further showed that malignant epithelial cells exhibit increased transcript complexity, widespread 3′ UTR shortening, reduced intron retention, and subtype-associated splicing programs, indicating that isoform-level regulation is closely linked to tumor-state diversification and cancer-specific RNA processing [123].

Collectively, these studies show that scLR-seq adds an isoform-resolved layer to biological frameworks established by conventional single-cell transcriptomics. Across immunology, neuroscience, and cancer biology, it reveals how transcript-structure variation contributes to the organization and regulation of cellular programs.

Challenges and Future Directions

Despite rapidly expanding applications, scLR-seq remains in transition from demonstration studies to routine use. The most immediate limitations arise from the experimental layer. Compared with conventional short-read scRNA-seq, long-read workflows still require trade-offs among throughput, cost, single-molecule accuracy, and recovery of full-length molecules [124]. Although long-read platforms have improved in accuracy and accessibility, long-read single-cell approaches generally yield lower effective transcript coverage per cell. Long molecules are also susceptible to degradation, truncation and amplification bias during reverse transcription and library preparation [10, 125]. In addition, errors in CB and UMI recovery introduce uncertainty into demultiplexing, molecule collapsing and dropout estimates [13, 126]. As a result, the primary bottleneck is no longer whether full-length transcripts can be captured, but whether they can be profiled in a stable, scalable, and cost-effective manner.

Computational analysis constitutes a second challenge. scLR-seq requires the coordinated processing of CB and UMI extraction, error correction, isoform quantification and downstream analyses of alternative splicing, TSS and TES usage, ASE and fusions. Bias introduced early in this workflow can propagate into subsequent inference. For instance, sequencing errors impair barcode recovery, whereas read truncation can distort isoform quantification [55, 116, 127]. The challenge is not a lack of tools, but rather a lack of stable, compatible analysis systems that can be easily migrated across different sequencing platforms and library strategies.

Furthermore, the information density of long reads also complicates statistical interpretation and reproducibility. Signals at the isoform, TSS, TES, or fusion levels are sparser than gene-level counts and depend more strongly on sequencing depth, sample size, and reference annotation quality [13]. While the discovery of novel isoforms is a major advantage of long-read sequencing, their biological relevance and reproducibility across cohorts cannot be assumed. Systematic benchmarks have shown significant performance differences among workflows for isoform profiling [50]. Public benchmark datasets, shared evaluation metrics and standardized cross-platform protocols will therefore be important for establishing reproducible practice.

As these challenges become clearer, future research directions are beginning to converge. Experimentally, increasing throughput, reducing input requirements and improving full-length

molecule recovery may narrow the gap with short-read scales. Computationally, joint models that integrate gene expression, isoform usage and sequence variation could replace isolated task-specific tools. Integration with short-read sequencing, spatial transcriptomics and other single-cell multi-omics modalities is also likely to be important [100]. As reference annotations and benchmark systems mature, scLR-seq may move from cataloguing transcript structures towards testing how those structures contribute to regulatory phenotypes and disease biology.

Conclusions

scLR-seq has rapidly emerged as a transformative technology, providing an unprecedented molecular view of transcriptomes at the level of full-length isoforms. By directly capturing transcript structure, alternative splicing, and transcription start and end sites, scLR-seq overcomes the inherent limitations of short-read approaches and enables precise characterization of cellular heterogeneity, developmental programs, and disease-associated transcriptomic changes. It has revealed novel isoforms, complex transcript architectures, and regulatory dynamics across immune, neural, and tumor systems, uncovering cell-type-specific splicing, promoter usage, and polyadenylation patterns that were previously inaccessible. These findings highlight the critical role of transcript structural complexity in defining cellular identity and function. While challenges remain in throughput, cost, and data analysis, ongoing developments in computational tools, multi-omics integration, and experimental scale promise to expand its impact. scLR-seq is poised to become a core tool for dissecting cellular programs, developmental trajectories, and disease mechanisms, offering a new level of resolution in transcriptomic research.

Abbreviations

APA: alternative polyadenylation; AS: alternative splicing; ASE: allele-specific expression; CB: cell barcode; CCS: circular consensus sequencing; CNA: copy number alteration; DSE: differential splicing events; DTU: differential transcript usage; EM: expectation–maximization; GWAS: genome-wide association study; LRS: long-read sequencing; LSH: locality-sensitive hashing; ONT: Oxford Nanopore Technologies; PacBio: Pacific Biosciences; QTL: quantitative trait locus; RBP: RNA-binding protein; RNN: Recurrent Neural Network; scLR-seq: single-cell long-read sequencing; scRNA-seq: single-cell RNA sequencing; SMRT: Single-Molecule Real-Time; SNP: single-nucleotide polymorphism; SNV: single nucleotide variants; SNV: single-nucleotide variants; T2T: telomere-to-telomere; TCC: transcript compatibility count; TE: transposable element; TES: transcription end site; TSS: transcription start site; UMI: unique molecular identifier

Declaration of Generative AI and AI-assisted Technologies in the Manuscript Preparation Process

During the preparation of this work, the authors used ChatGPT to polish the manuscript. After using this tool, the authors reviewed and edited the content as needed and take full responsibility for the content of the published article.

Acknowledgements

We thank all of our team members. This work was supported by the National Key Research and Development Program of China (2025YFC3409300), Guangdong Basic and Applied Basic Research Foundation (2026A1515012050 and 2024B1515230003), and the Shenzhen Key Laboratory of Single-Cell Omics (ZDSYS20190902093613831).

Author Contributions

Chuanyu Liu (Conceptualization, Supervision, Writing–review & editing), Ze-Hui Ren (Investigation, Writing–original draft, Visualization), Wenteng Liu (Writing–original draft, Visualization), Jianhua Yin (Writing–review & editing).

Data Availability

not applicable

Competing Interests

The authors declare no competing financial interests.

References

1. Boxer E, Feigin N, Tschernichovsky R, et al. Emerging clinical applications of single-cell RNA sequencing in oncology. Nature reviews. Clinical oncology 2025;**22**(5):315-326. doi:10.1038/s41571-025-01003-3.
2. Gulati GS, D'Silva JP, Liu Y, et al. Profiling cell identity and tissue architecture with single-cell and spatial transcriptomics. Nature reviews. Molecular cell biology 2025;**26**(1):11-31. doi:10.1038/s41580-024-00768-2.
3. Rood JE, Maartens A, Hupalowska A, et al. Impact of the Human Cell Atlas on medicine. Nat Med 2022;**28**(12):2486-2496. doi:10.1038/s41591-022-02104-7.
4. Yin J, Zheng Y, Huang Z, et al. Chinese Immune Multi-Omics Atlas. Science 2026;**391**(6781):eadt3130. doi:10.1126/science.adt3130.
5. Cha J, Lee I. Single-cell network biology for resolving cellular heterogeneity in human diseases. Exp Mol Med 2020;**52**(11):1798-1808. doi:10.1038/s12276-020-00528-0.
6. Grün D, Lyubimova A, Kester L, et al. Single-cell messenger RNA sequencing reveals rare intestinal cell types. Nature 2015;**525**(7568):251-255. doi:10.1038/nature14966.
7. Jindal A, Gupta P, Jayadeva, et al. Discovery of rare cells from voluminous single cell expression data. Nat Commun 2018;**9**(1):4719. doi:10.1038/s41467-018-07234-6.
8. Tirosh I, Suva ML. Cancer cell states: Lessons from ten years of single-cell RNA-sequencing of human tumors. Cancer Cell 2024;**42**(9):1497-1506. doi:10.1016/j.ccell.2024.08.005.
9. Ament IH, Debruyne N, Wang F, et al. Long-read RNA sequencing: A transformative technology for exploring transcriptome complexity in human diseases. Mol Ther 2025;**33**(3):883-894. doi:10.1016/j.ymthe.2024.11.025.
10. Chen Y, Davidson NM, Wan YK, et al. A systematic benchmark of Nanopore long-read RNA sequencing for transcript-level analysis in human cell lines. Nat Methods 2025;**22**(4):801-812. doi:10.1038/s41592-025-02623-4.
11. Iwabuchi S, Nasti A, Okada H, et al. Systematic evaluation of long- and short-read RNA-seq for human peripheral blood. NAR molecular medicine 2026;**3**(1):ugag006. doi:10.1093/narmme/ugag006.

12. Pardo-Palacios FJ, Wang D, Reese F, et al. Systematic assessment of long-read RNA-seq methods for transcript identification and quantification. Nat Methods 2024;**21**(7):1349-1363. doi:10.1038/s41592-024-02298-3.
13. Bhatia S, Field MA, Hebbard L, et al. Bioinformatics frameworks for single-cell long-read sequencing: unlocking isoform-level resolution. Brief Bioinform 2025;**26**(6):bbaf655. doi:10.1093/bib/bbaf655.
14. Kumari P, Kaur M, Dindhoria K, et al. Advances in long-read single-cell transcriptomics. Hum Genet 2024;**143**(9-10):1005-1020. doi:10.1007/s00439-024-02678-x.
15. Wen L, Tang F. Single-cell omics sequencing technologies: the long-read generation. Trends Genet 2026;**42**(1):46-62. doi:10.1016/j.tig.2025.07.012.
16. Deng E, Shen Q, Zhang J, et al. Systematic evaluation of single-cell RNA-seq analyses performance based on long-read sequencing platforms. J Adv Res 2025;**71**:141-153. doi:10.1016/j.jare.2024.05.020.
17. Monzó C, Liu T, Conesa A. Transcriptomics in the era of long-read sequencing. Nature reviews. Genetics 2025;**26**(10):681-701. doi:10.1038/s41576-025-00828-z.
18. Wang Y, Zhao Y, Bollas A, et al. Nanopore sequencing technology, bioinformatics and applications. Nat Biotechnol 2021;**39**(11):1348-1365. doi:10.1038/s41587-021-01108-x.
19. Gupta P, O'Neill H, Wolvetang EJ, et al. Advances in single-cell long-read sequencing technologies. NAR Genom Bioinform 2024;**6**(2):lqae047. doi:10.1093/nargab/lqae047.
20. Wang B, Jia P, Gao S, et al. Long and Accurate: How HiFi Sequencing is Transforming Genomics. Genomics, proteomics & bioinformatics 2025;**23**(1):qzaf003. doi:10.1093/gpbjnl/qzaf003.
21. Wissel D, Mehlferber MM, Nguyen KM, et al. A systematic benchmark of high-accuracy PacBio long-read RNA sequencing for transcript-level quantification. Genome Biol 2026;**27**(1):110. doi:10.1186/s13059-026-03988-1.
22. Kasianowicz JJ, Bezrukov SM. On 'three decades of nanopore sequencing'. Nat Biotechnol 2016;**34**(5):481-482. doi:10.1038/nbt.3570.
23. Pagès-Gallego M, de Ridder J. Comprehensive benchmark and architectural analysis of deep learning models for nanopore sequencing basecalling. Genome Biol 2023;**24**(1):71. doi:10.1186/s13059-023-02903-2.
24. Wan YK, Hendra C, Pratanwanich PN, et al. Beyond sequencing: machine learning algorithms extract biology hidden in Nanopore signal data. Trends Genet 2022;**38**(3):246-257. doi:10.1016/j.tig.2021.09.001.
25. Dorado (2026). Dorado (Version 1.4) https://github.com/nanoporetech/dorado/
26. Zhong ZD, Xie YY, Chen HX, et al. Systematic comparison of tools used for m(6)A mapping from nanopore direct RNA sequencing. Nat Commun 2023;**14**(1):1906. doi:10.1038/s41467-023-37596-5.
27. Geyer J, Opoku KB, Lin J, et al. Real-time genomic characterization of pediatric acute leukemia using adaptive sampling. Leukemia 2025;**39**(5):1069-1077. doi:10.1038/s41375-025-02565-y.
28. Nurk S, Koren S, Rhie A, et al. The complete sequence of a human genome. Science 2022;**376**(6588):44-53. doi:10.1126/science.abj6987.
29. Manuel JG, Heins HB, Crocker S, et al. High Coverage Highly Accurate Long-Read Sequencing of a Mouse Neuronal Cell Line Using the PacBio Revio Sequencer. bioRxiv 2023:2023.06.06.543940. doi:10.1101/2023.06.06.543940.
30. Ni Y, Liu X, Simeneh ZM, et al. Benchmarking of Nanopore R10.4 and R9.4.1 flow cells in single-cell whole-genome amplification and whole-genome shotgun sequencing. Comput Struct

Biotechnol J 2023;**21**:2352-2364. doi:10.1016/j.csbj.2023.03.038.
31. Gupta I, Collier PG, Haase B, et al. Single-cell isoform RNA sequencing characterizes isoforms in thousands of cerebellar cells. Nat Biotechnol 2018:10.1038/nbt.4259. doi:10.1038/nbt.4259.
32. Lebrigand K, Magnone V, Barbry P, et al. High throughput error corrected Nanopore single cell transcriptome sequencing. Nat Commun 2020;**11**(1):4025. doi:10.1038/s41467-020-17800-6.
33. Tian L, Jabbari JS, Thijssen R, et al. Comprehensive characterization of single-cell full-length isoforms in human and mouse with long-read sequencing. Genome Biol 2021;**22**(1):310. doi:10.1186/s13059-021-02525-6.
34. Rebboah E, Reese F, Williams K, et al. Mapping and modeling the genomic basis of differential RNA isoform expression at single-cell resolution with LR-Split-seq. Genome Biol 2021;**22**(1):286. doi:10.1186/s13059-021-02505-w.
35. Singh M, Al-Eryani G, Carswell S, et al. High-throughput targeted long-read single cell sequencing reveals the clonal and transcriptional landscape of lymphocytes. Nat Commun 2019;**10**(1):3120. doi:10.1038/s41467-019-11049-4.
36. Philpott M, Watson J, Thakurta A, et al. Nanopore sequencing of single-cell transcriptomes with scCOLOR-seq. Nat Biotechnol 2021;**39**(12):1517-1520. doi:10.1038/s41587-021-00965-w.
37. Sun J, Philpott M, Loi D, et al. Enhancing single-cell transcriptomics using interposed anchor oligonucleotide sequences. Commun Biol 2025;**8**(1):67. doi:10.1038/s42003-025-07474-5.
38. Volden R, Palmer T, Byrne A, et al. Improving nanopore read accuracy with the R2C2 method enables the sequencing of highly multiplexed full-length single-cell cDNA. Proc Natl Acad Sci U S A 2018;**115**(39):9726-9731. doi:10.1073/pnas.1806447115.
39. You Y, Prawer YDJ, De Paoli-Iseppi R, et al. Identification of cell barcodes from long-read single-cell RNA-seq with BLAZE. Genome Biol 2023;**24**(1):66. doi:10.1186/s13059-023-02907-y.
40. Shiau CK, Lu L, Kieser R, et al. High throughput single cell long-read sequencing analyses of same-cell genotypes and phenotypes in human tumors. Nat Commun 2023;**14**(1):4124. doi:10.1038/s41467-023-39813-7.
41. Cheng O, Ling MH, Wang C, et al. Flexiplex: a versatile demultiplexer and search tool for omics data. Bioinformatics 2024;**40**(3):btae102. doi:10.1093/bioinformatics/btae102.
42. Hamraoui A, Onfroy A, Senamaud-Beaufort C, et al. A systematic benchmark of bioinformatics methods for single-cell and spatial RNA-seq Nanopore long-read data. bioRxiv 2025. doi:10.1101/2025.07.21.665920.
43. Deserranno K, Callens E, Berrevoet D, et al. Plate-based long-read single cell gene- and isoform transcriptome profiling using scLIS-seq. Research Square 2025. doi:10.21203/rs.3.rs-6217988/v1.
44. Al'Khafaji AM, Smith JT, Garimella KV, et al. High-throughput RNA isoform sequencing using programmed cDNA concatenation. Nat Biotechnol 2024;**42**(4):582-586. doi:10.1038/s41587-023-01815-7.
45. Fan X, Tang D, Liao Y, et al. Single-cell RNA-seq analysis of mouse preimplantation embryos by third-generation sequencing. PLoS Biol 2020;**18**(12):e3001017. doi:10.1371/journal.pbio.3001017.
46. Liao Y, Liu Z, Zhang Y, et al. High-throughput and high-sensitivity full-length single-cell RNA-seq analysis on third-generation sequencing platform. Cell Discov 2023;**9**(1):5. doi:10.1038/s41421-022-00500-4.
47. Pandey AC, Bezney J, Deascanis D, et al. A CRISPR/Cas9-based enhancement of

high-throughput single-cell transcriptomics. Nat Commun 2025;**16**(1):4664. doi:10.1038/s41467-025-59880-2.

48. Peng H, Jabbari JS, Tian L, et al. Single-cell Rapid Capture Hybridization sequencing reliably detects isoform usage and coding mutations in targeted genes. Genome Res 2025;**35**(4):942-955. doi:10.1101/gr.279322.124.
49. Isakova A, Neff N, Quake SR. Single-cell quantification of a broad RNA spectrum reveals unique noncoding patterns associated with cell types and states. Proc Natl Acad Sci U S A 2021;**118**(51):e2113568118. doi:10.1073/pnas.2113568118.
50. Scoones ALA, Lan Y, Utting C, et al. A comparison of long-read single-cell transcriptomic approaches. bioRxiv 2025. doi:10.1101/2025.07.03.662955.
51. Kim HS, Grimes SM, Hooker AC, et al. Single-cell characterization of CRISPR-modified transcript isoforms with nanopore sequencing. Genome Biol 2021;**22**(1):331. doi:10.1186/s13059-021-02554-1.
52. Long Y, Liu Z, Jia J, et al. FlsnRNA-seq: protoplasting-free full-length single-nucleus RNA profiling in plants. Genome Biol 2021;**22**(1):66. doi:10.1186/s13059-021-02288-0.
53. wf-single-cell (2026). wf-single-cell (Version 3.3.4) https://github.com/epi2me-labs/wf-single-cell
54. Ebrahimi G, Orabi B, Robinson M, et al. Fast and accurate matching of cellular barcodes across short-reads and long-reads of single-cell RNA-seq experiments. iScience 2022;**25**(7):104530. doi:10.1016/j.isci.2022.104530.
55. Fu Y, Kim H, Roy S, et al. Single cell and spatial alternative splicing analysis with Nanopore long read sequencing. Nat Commun 2025;**16**(1):6654. doi:10.1038/s41467-025-60902-2.
56. Wang Q, Bönigk S, Böhm V, et al. Single-cell transcriptome sequencing on the Nanopore platform with ScNapBar. RNA 2021;**27**(7):763-770. doi:10.1261/rna.078154.120.
57. Trull A, Worthey EA, Ianov L. scnanoseq: an nf-core pipeline for Oxford Nanopore single-cell RNA-sequencing. Bioinformatics 2025;**41**(9):btaf487. doi:10.1093/bioinformatics/btaf487.
58. Calvo-Roitberg E, Daniels RF, Pai AA. Challenges in identifying mRNA transcript starts and ends from long-read sequencing data. Genome Res 2024;**34**(11):1719-1734. doi:10.1101/gr.279559.124.
59. Guo LT, Olson S, Patel S, et al. Direct tracking of reverse-transcriptase speed and template sensitivity: implications for sequencing and analysis of long RNA molecules. Nucleic Acids Res 2022;**50**(12):6980-6989. doi:10.1093/nar/gkac518.
60. Sessegolo C, Cruaud C, Da Silva C, et al. Transcriptome profiling of mouse samples using nanopore sequencing of cDNA and RNA molecules. Sci Rep 2019;**9**(1):14908. doi:10.1038/s41598-019-51470-9.
61. Workman RE, Tang AD, Tang PS, et al. Nanopore native RNA sequencing of a human poly(A) transcriptome. Nat Methods 2019;**16**(12):1297-1305. doi:10.1038/s41592-019-0617-2.
62. Chen Y, Sim A, Wan YK, et al. Context-aware transcript quantification from long-read RNA-seq data with Bambu. Nat Methods 2023;**20**(8):1187-1195. doi:10.1038/s41592-023-01908-w.
63. Prjibelski AD, Mikheenko A, Joglekar A, et al. Accurate isoform discovery with IsoQuant using long reads. Nat Biotechnol 2023;**41**(7):915-918. doi:10.1038/s41587-022-01565-y.
64. IsoSeq (2025). IsoSeq (Version 4.3.0) https://github.com/pacificbiosciences/isoseq
65. Sim A, Ling MH, Chen Y, et al. Isoform-level discovery, quantification and fusion analysis from single-cell and spatial long-read RNA-seq data with Bambu-Clump. bioRxiv 2025. doi:10.1101/2024.12.30.630828.
66. Kabza M, Ritter A, Byrne A, et al. Accurate long-read transcript discovery and quantification at

single-cell, pseudo-bulk and bulk resolution with Isosceles. Nat Commun 2024;**15**(1):7316. doi:10.1038/s41467-024-51584-3.

67. Yu H, Georgescu CH, Khorgade A, et al. Accurate strand-specific long-read transcript isoform discovery and quantification at bulk, single-cell, and single-nucleus resolution. bioRxiv 2026:2026.02.12.705617. doi:10.64898/2026.02.12.705617.

68. Volden R, Schimke KD, Byrne A, et al. Identifying and quantifying isoforms from accurate full-length transcriptome sequencing reads with Mandalorion. Genome Biol 2023;**24**(1):167. doi:10.1186/s13059-023-02999-6.

69. Wyman D, Balderrama-Gutierrez G, Reese F, et al. A technology-agnostic long-read analysis pipeline for transcriptome discovery and quantification. bioRixv 2019. doi:10.1101/672931.

70. Tang AD, Felton C, Hrabeta-Robinson E, et al. Detecting haplotype-specific transcript variation in long reads with FLAIR2. Genome Biol 2024;**25**(1):173. doi:10.1186/s13059-024-03301-y.

71. Dong X, Du MRM, Gouil Q, et al. Benchmarking long-read RNA-sequencing analysis tools using in silico mixtures. Nat Methods 2023;**20**(11):1810-1821. doi:10.1038/s41592-023-02026-3.

72. Gao Y, Wang F, Wang R, et al. ESPRESSO: Robust discovery and quantification of transcript isoforms from error-prone long-read RNA-seq data. Sci Adv 2023;**9**(3):eabq5072. doi:10.1126/sciadv.abq5072.

73. Tang AD, Soulette CM, van Baren MJ, et al. Full-length transcript characterization of SF3B1 mutation in chronic lymphocytic leukemia reveals downregulation of retained introns. Nat Commun 2020;**11**(1):1438. doi:10.1038/s41467-020-15171-6.

74. Kovaka S, Zimin AV, Pertea GM, et al. Transcriptome assembly from long-read RNA-seq alignments with StringTie2. Genome Biol 2019;**20**(1):278. doi:10.1186/s13059-019-1910-1.

75. Zare Jousheghani Z, Singh NP, Patro R. Oarfish: enhanced probabilistic modeling leads to improved accuracy in long read transcriptome quantification. Bioinformatics 2025;**41**(Supplement_1):i304-i313. doi:10.1093/bioinformatics/btaf240.

76. Loving RK, Sullivan DK, Reese F, et al. Long-read sequencing transcriptome quantification with lr-kallisto. PLoS Comput Biol 2025;**21**(12):e1013692. doi:10.1371/journal.pcbi.1013692.

77. Zhang Z, Luo D, Zhong X, et al. SCINA: A Semi-Supervised Subtyping Algorithm of Single Cells and Bulk Samples. Genes (Basel) 2019;**10**(7):531. doi:10.3390/genes10070531.

78. Shao X, Liao J, Lu X, et al. scCATCH: Automatic Annotation on Cell Types of Clusters from Single-Cell RNA Sequencing Data. iScience 2020;**23**(3):100882. doi:10.1016/j.isci.2020.100882.

79. Aran D, Looney AP, Liu L, et al. Reference-based analysis of lung single-cell sequencing reveals a transitional profibrotic macrophage. Nat Immunol 2019;**20**(2):163-172. doi:10.1038/s41590-018-0276-y.

80. Fu R, Gillen AE, Sheridan RM, et al. clustifyr: an R package for automated single-cell RNA sequencing cluster classification. F1000Research 2020;**9**:223. doi:10.12688/f1000research.22969.2.

81. Byrne A, Beaudin AE, Olsen HE, et al. Nanopore long-read RNAseq reveals widespread transcriptional variation among the surface receptors of individual B cells. Nat Commun 2017;**8**:16027. doi:10.1038/ncomms16027.

82. Lim C, An H, Park J. Identification of cell-type-specific, transcriptionally active transposable elements using long-read RNA-sequencing data-based comprehensive annotation. Genomics & informatics 2025;**23**(1):17. doi:10.1186/s44342-025-00048-1.

83. Wijewardena H, Wu S, Schmitz U. ICON: An isoform-aware hierarchical random forest model

for cell type classification. bioRxiv : the preprint server for biology 2026:2026.04.28.721306. doi:10.64898/2026.04.28.721306.

84. Bi Y, Lankenau TL, Lienhard M, et al. IsoTools 2.0: Software for Comprehensive Analysis of Long-read Transcriptome Sequencing Data. J Mol Biol 2025;**437**(15):169049. doi:10.1016/j.jmb.2025.169049.

85. Stachelek K, Bhat B, Cobrinik D. Chevreul: an R bioconductor package for exploratory analysis of full-length single cell sequencing. GigaByte 2025;**2025**:gigabyte158. doi:10.46471/gigabyte.158.

86. scCyclone (2025). scCyclone (Version 1.1.0) https://github.com/dawangran/scCyclone

87. Vitting-Seerup K, Sandelin A. IsoformSwitchAnalyzeR: analysis of changes in genome-wide patterns of alternative splicing and its functional consequences. Bioinformatics 2019;**35**(21):4469-4471. doi:10.1093/bioinformatics/btz247.

88. Fu S, Li WV. Predicting and comparing transcription start sites in single cell populations. PLoS Comput Biol 2025;**21**(4):e1012878. doi:10.1371/journal.pcbi.1012878.

89. Shulman ED, Elkon R. Cell-type-specific analysis of alternative polyadenylation using single-cell transcriptomics data. Nucleic Acids Res 2019;**47**(19):10027-10039. doi:10.1093/nar/gkz781.

90. An H, Choi SY, Yi J, et al. Mouse Single-Cell Long-Read Splicing Atlas by Ouro-Seq. bioRxiv 2025. doi:10.1101/2025.01.17.633678.

91. Dondi A, Borgsmüller N, Ferreira PF, et al. De novo detection of somatic variants in high-quality long-read single-cell RNA sequencing data. Genome Res 2025;**35**(4):900-913. doi:10.1101/gr.279281.124.

92. Petrova V, Niu M, Vierbuchen TS, et al. ASPEN: Robust detection of allelic dynamics in single cell RNA-seq. PLoS Comput Biol 2025;**21**(12):e1013837. doi:10.1371/journal.pcbi.1013837.

93. Shiau CK, Lin HY, Pan T, et al. Fusion gene discovery in single cells from high throughput long read single cell transcriptomes. bioRxiv 2026:2026.01.13.699333. doi:10.64898/2026.01.13.699333.

94. Qin Q, Popic V, Wienand K, et al. Accurate fusion transcript identification from long- and short-read isoform sequencing at bulk or single-cell resolution. Genome Res 2025;**35**(4):967-986. doi:10.1101/gr.279200.124.

95. He J, Babarinde IA, Sun L, et al. Identifying transposable element expression dynamics and heterogeneity during development at the single-cell level with a processing pipeline scTE. Nat Commun 2021;**12**(1):1456. doi:10.1038/s41467-021-21808-x.

96. Rodríguez-Quiroz RO, Valdebenito-Maturana B. SoloTE for improved analysis of transposable elements in single-cell RNA-Seq data using locus-specific expression. Commun Biol 2022;**5**(1):1063. doi:10.1038/s42003-022-04020-5.

97. Berrens RV, Yang A, Laumer CE, et al. Locus-specific expression of transposable elements in single cells with CELLO-seq. Nat Biotechnol 2022;**40**(4):546-554. doi:10.1038/s41587-021-01093-1.

98. Ren Z, He J, Huang X, et al. Isoform characterization of m(6)A in single cells identifies its role in RNA surveillance. Nat Commun 2025;**16**(1):5828. doi:10.1038/s41467-025-60869-0.

99. Lin J, Xue X, Wang Y, et al. scNanoCOOL-seq: a long-read single-cell sequencing method for multi-omics profiling within individual cells. Cell Res 2023;**33**(11):879-882. doi:10.1038/s41422-023-00873-5.

100. Pančíková A, Cools R, Eftychiou M, et al. Long-read single-cell genome, transcriptome and open chromatin profiling links genotype to phenotypes. bioRxiv 2025.

doi:10.1101/2025.09.08.674950.

101. Hu W, Foord C, Hsu J, et al. Combined single-cell profiling of chromatin-transcriptome and splicing across brain cell types, regions and disease state. Nat Biotechnol 2025:10.1038/s41587-025-02734-5. doi:10.1038/s41587-025-02734-5.

102. Chang L, Deng E, Wang J, et al. Single-cell third-generation sequencing-based multi-omics uncovers gene expression changes governed by ecDNA and structural variants in cancer cells. Clin Transl Med 2023;**13**(8):e1351. doi:10.1002/ctm2.1351.

103. Liu S, Chen X, Huang X, et al. AEnet: a practical tool to construct the splicing-associated phenotype atlas at a single cell level. Gigascience 2025;**14**:giaf110. doi:10.1093/gigascience/giaf110.

104. De Rijk P, Watzeels T, Küçükali F, et al. Scywalker: scalable end-to-end data analysis workflow for long-read single-cell transcriptome sequencing. Bioinformatics 2024;**40**(9):btae549. doi:10.1093/bioinformatics/btae549.

105. Alfonso-Gonzalez C, Hilgers VRE. (Alternative) transcription start sites as regulators of RNA processing. Trends Cell Biol 2024;**34**(12):1018-1028. doi:10.1016/j.tcb.2024.02.010.

106. Weber R, Ghoshdastider U, Spies D, et al. Monitoring the 5'UTR landscape reveals isoform switches to drive translational efficiencies in cancer. Oncogene 2023;**42**(9):638-650. doi:10.1038/s41388-022-02578-2.

107. Qi G, Battle A. Computational methods for allele-specific expression in single cells. Trends Genet 2024;**40**(11):939-949. doi:10.1016/j.tig.2024.07.003.

108. Roehrig AL, Hirsch TZ, Pire A, et al. Single-cell multiomics reveals the interplay of clonal evolution and cellular plasticity in hepatoblastoma. Nat Commun 2024;**15**(1):3031. doi:10.1038/s41467-024-47280-x.

109. Xu Z, Wang K. LongAllele: a joint inference framework for allele-specific analysis on long-read bulk and single-cell RNA sequencing. bioRxiv 2026:2026.05.05.722992. doi:10.64898/2026.05.05.722992.

110. Li B, Luong T, Sisay E, et al. Single-cell full-length transcriptome of human lung reveals genetic effects on isoform regulation beyond gene-level expression. bioRxiv 2026. doi:10.64898/2026.03.27.714873.

111. Walter W, Kern W, Stengel A. Long-read single-cell isoform sequencing for cell type-specific detection of genomic rearrangement-dependent and -independent fusion transcripts. Blood 2025;**146**(Supplement 1):6117.

112. Marlow SA, Deaville LA, Berrens RV. Long-read RNA sequencing of transposable elements from single cells using CELLO-seq. Nat Protoc 2025;**20**(11):3070-3095. doi:10.1038/s41596-025-01203-2.

113. Inamo J, Suzuki A, Ueda MT, et al. Long-read sequencing for 29 immune cell subsets reveals disease-linked isoforms. Nat Commun 2024;**15**(1):4285. doi:10.1038/s41467-024-48615-4.

114. Foord C, Prjibelski AD, Hu W, et al. A spatial long-read approach at near-single-cell resolution reveals developmental regulation of splicing and polyadenylation sites in distinct cortical layers and cell types. Nat Commun 2025;**16**(1):8093. doi:10.1038/s41467-025-63301-9.

115. Liu X, Li F, Czosnyka M, et al. Multi-omics and high-spatial-resolution omics: deciphering complexity in neurological disorders. Gigascience 2025;**14**:giaf137. doi:10.1093/gigascience/giaf137.

116. Doyle PH, Page ML, Brandon JA, et al. Decoding the human PBMC isonome: Isoform-level resolution with single-cell long-read transcriptomics. bioRxiv 2025:2025.10.16.682832.

doi:10.1101/2025.10.16.682832.

117. Zheng Y, Ren ZH, Yang Y, et al. Single-Cell Multi-Omics Dissects Transcript Isoform and Immune Repertoire Dynamics in Human Immunosenescence. bioRxiv 2025. doi:10.1101/2025.11.21.689731.

118. Joglekar A, Hu W, Zhang B, et al. Single-cell long-read sequencing-based mapping reveals specialized splicing patterns in developing and adult mouse and human brain. Nat Neurosci 2024;**27**(6):1051-1063. doi:10.1038/s41593-024-01616-4.

119. Patowary A, Zhang P, Jops C, et al. Developmental isoform diversity in the human neocortex informs neuropsychiatric risk mechanisms. Science 2024;**384**(6698):eadh7688. doi:10.1126/science.adh7688.

120. Belchikov N, Hu W, Fan L, et al. A single-cell, long-read, isoform-resolved case-control study of FTD reveals cell-type-specific and broad splicing dysregulation in human brain. Cell Rep 2025;**44**(9):116198. doi:10.1016/j.celrep.2025.116198.

121. Dondi A, Lischetti U, Jacob F, et al. Detection of isoforms and genomic alterations by high-throughput full-length single-cell RNA sequencing in ovarian cancer. Nat Commun 2023;**14**(1):7780. doi:10.1038/s41467-023-43387-9.

122. Penter L, Borji M, Nagler A, et al. Integrative genotyping of cancer and immune phenotypes by long-read sequencing. Nat Commun 2024;**15**(1):32. doi:10.1038/s41467-023-44137-7.

123. Lu P, Zhang Y, Cui Y, et al. Systematic characterization of full-length RNA isoforms in human colorectal cancer at single-cell resolution. Protein Cell 2025;**16**(10):873-895. doi:10.1093/procel/pwaf049.

124. Shi H, Zhou Y, Jia E, et al. Bias in RNA-seq Library Preparation: Current Challenges and Solutions. Biomed Res Int 2021;**2021**:6647597. doi:10.1155/2021/6647597.

125. Prawer YDJ, Gleeson J, De Paoli-Iseppi R, et al. Pervasive effects of RNA degradation on Nanopore direct RNA sequencing. NAR Genom Bioinform 2023;**5**(2):lqad060. doi:10.1093/nargab/lqad060.

126. Belchikov N, Hsu J, Li XJ, et al. Understanding isoform expression by pairing long-read sequencing with single-cell and spatial transcriptomics. Genome Res 2024;**34**(11):1735-1746. doi:10.1101/gr.279640.124.

127. Alfonso-Gonzalez C, Hilgers VRE. Elucidating the coordination of RNA processing using short-read and long-read RNA-sequencing methods. Nature reviews. Molecular cell biology 2026;**27**(3):194-212. doi:10.1038/s41580-025-00895-4.

Click here to access/download;Figure;Figure1.pdf

Figure 1

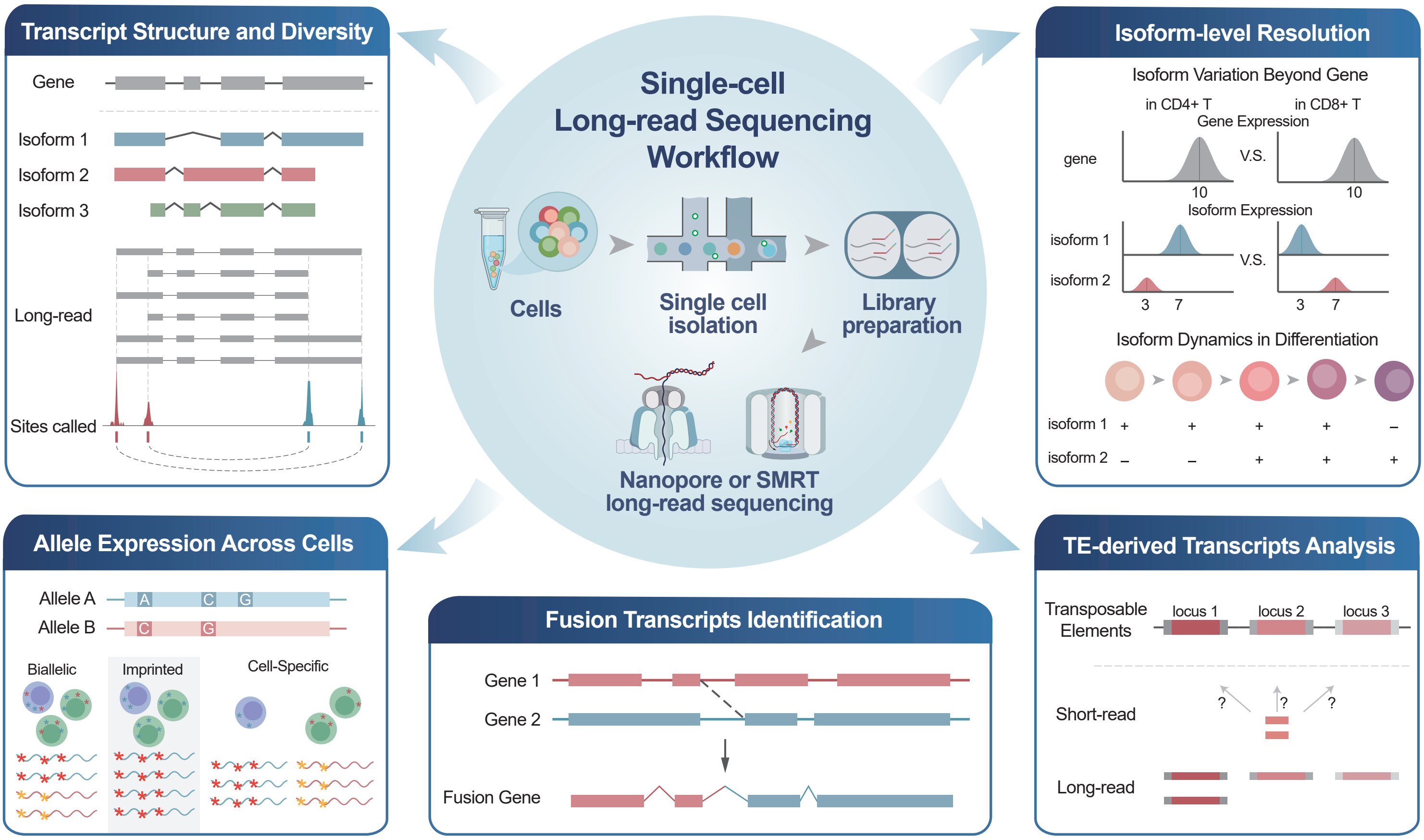

Figure2

Click here to access/download;Figure;Figure2.pdf

Figure 2

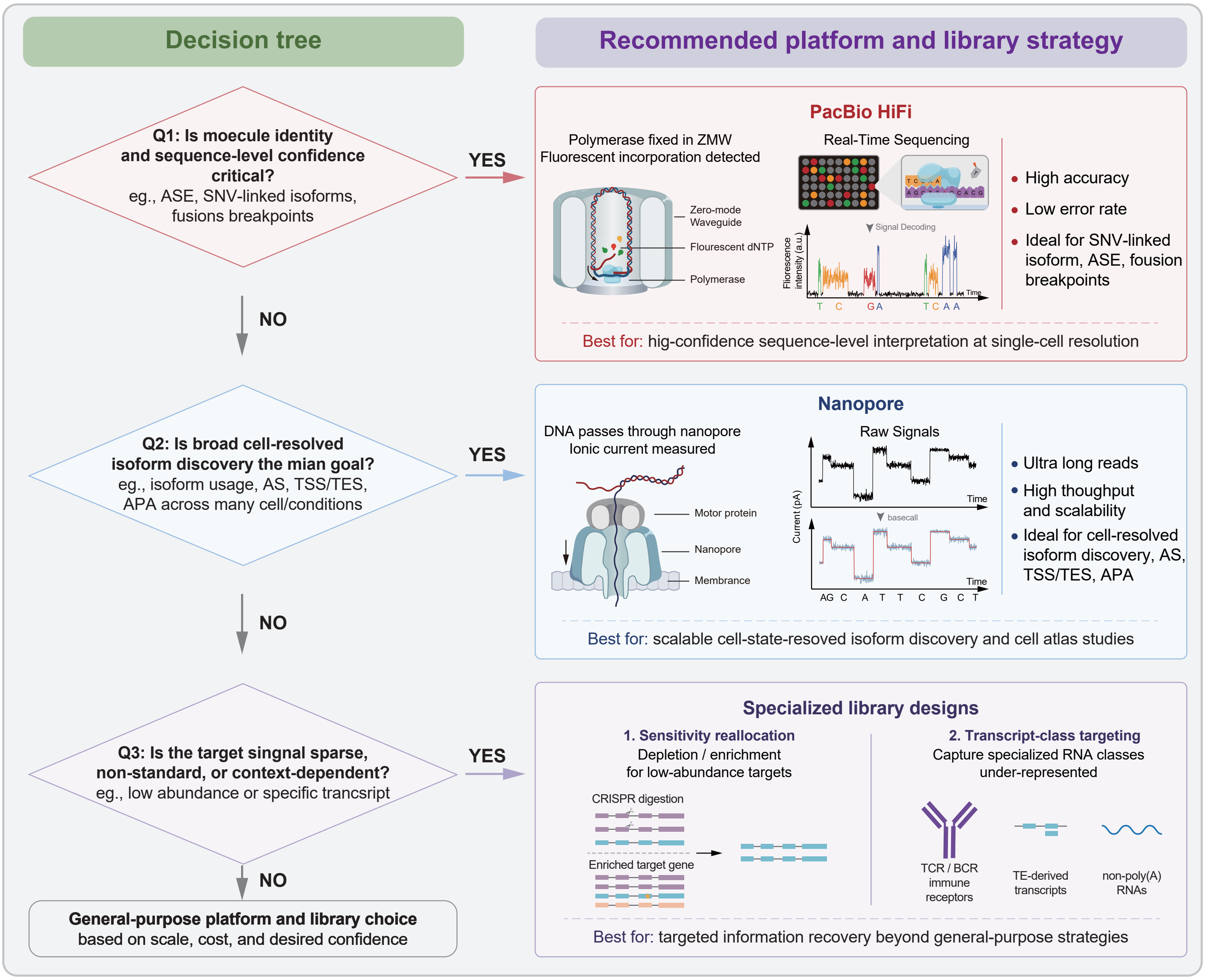

Figure 3

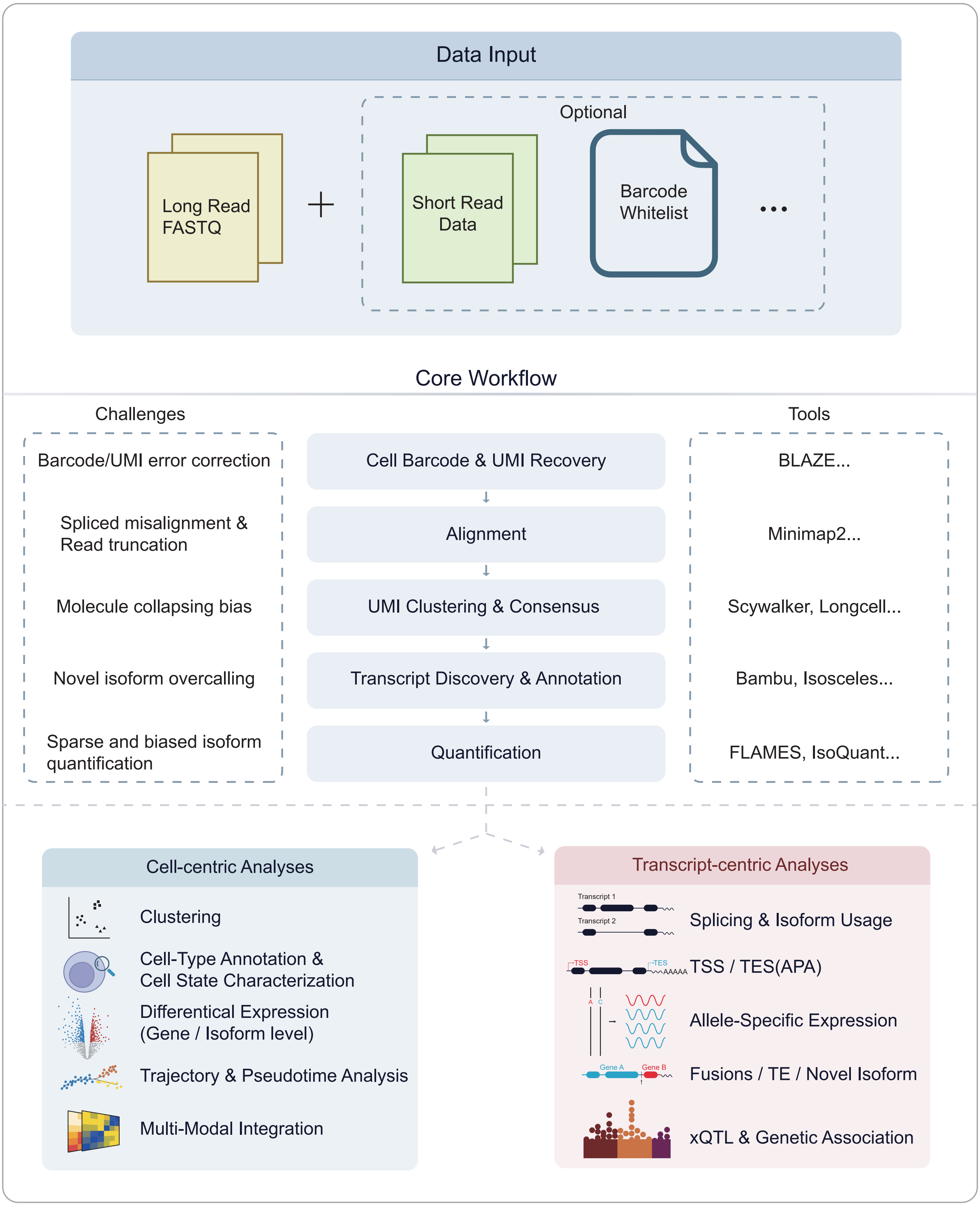
Data Input
Optional
Long Read FASTQ
+
Short Read Data
Barcode Whitelist
...
Core Workflow
Challenges
Barcode/UMI error correction
Spliced misalignment & Read truncation
Molecule collapsing bias
Novel isoform overcalling
Sparse and biased isoform quantification
Cell Barcode & UMI Recovery
Alignment
UMI Clustering & Consensus
Transcript Discovery & Annotation
Quantification
Tools
BLAZE...
Minimap2...
Scywalker, Longcell...
Bambu, Isosceles...
FLAMES, IsoQuant...
Cell-centric Analyses
Clustering
Cell-Type Annotation & Cell State Characterization
Differential Expression (Gene / Isoform level)
Trajectory & Pseudotime Analysis
Multi-Modal Integration
Transcript-centric Analyses
Transcript 1
Transcript 2
Splicing & Isoform Usage
TSS
TES
AAAAA
TSS / TES(APA)
Allele-Specific Expression
Gene A
Gene B
Fusions / TE / Novel Isoform
xQTL & Genetic Association

Click here to access/download
Supplementary Material
Revised Manuscript Track Changes.docx

Point by point letter

Click here to access/download
Supplementary Material
Response_to_Editor_and_Reviewers.docx

Cover Letter
Click here to access/download;Personal Cover;Cover_letter.docx

华大生命科学研究院
BGI·Research

Jun 22nd, 2026

Dear Editor,

We are pleased to resubmit our revised manuscript, "Single-cell Long-read Transcriptomics: From Technologies to Biological Insights" by Ren et al., for consideration in ***GigaScience***. We sincerely thank you and the reviewers for the careful evaluation of our manuscript and for the constructive comments. We have considered all comments in detail and have substantially revised the manuscript in this revision.

The major revisions include the following:

1. We substantially reorganized and streamlined the manuscript to improve its focus, coherence, and accessibility. The revision reframes the review around the major experimental and analytical decisions in scLR-seq, rather than presenting the field as a method-by-method catalogue, and the text was carefully edited to remove repetition, correct terminology, and improve readability.

2. We clarified the methodological organization and visual presentation of the review. In particular, the distinction between library construction strategies and computational analysis methods was made more explicit, the corresponding tables and sections were reorganized, and the original Figures 2 and 3 were integrated into a single decision-oriented figure to better guide platform selection and workflow design.

3. We strengthened the critical and practical value of the manuscript by adding quantitative benchmarking and reproducibility considerations, expanding the discussions of cell-type annotation, neurodevelopment, and oncology, and introducing dedicated sections on multi-omics integration and RNA modifications.

All corresponding revisions have been incorporated into the manuscript, and detailed point-by-point responses are provided in the accompanying Response Letter.

Thank you for reconsidering our manuscript. We hope that the revised version is now suitable for publication in ***GigaScience***.

Best regards,
Sincerely,

Chuanyu Liu
BGI-Research, Shenzhen, China
