# Supplementary material for: Single-cell long-read transcriptomics: from technologies to biological insights
Source: Gigascience. 2026 Jul 22;15:giag082. doi: 10.1093/gigascience/giag082 (PMC13508701; doi:10.1093/gigascience/giag082)
Supplement: Supplementary file 6 [file giag082_reviewer_1_report_original_submission.pdf]

Reviewer Report

Title: Single-cell Long-read Transcriptomics: From Technologies to Biological Insights

Version: Original Submission **Date:** 5/8/2026

Reviewer name: Reviewer 1

Reviewer Comments to Author:

This is a well-structured and timely review that covers the scLR-seq landscape comprehensively. It will be a useful reference for the field, though several areas need strengthening before publication.Strengths: The review covers the full pipeline from sequencing platforms to biological applications in a logical sequence. The inclusion of four detailed comparison tables (library strategies, demultiplexing tools, transcript discovery tools, functional analysis tools) is genuinely useful and not commonly found consolidated in a single review. Figures 1-4 are clear and well-designed.Weaknesses:1. There is a clear copy-paste error on lines 59-60: "These platforms have effectively . These platforms have effectively overcome the fragmentation issues…" More broadly, several sentences in the Background are overly long and would benefit from restructuring for clarity.2. Cell Type Annotation Section is Underdeveloped - The discussion of cell type annotation is notably leaner than other sections. The authors correctly identify the gap, that isoform-level annotation frameworks are lacking, but offer only a brief speculatiion on future directions.3. Computational Challenges Section Needs Quantitative Grounding - The challenges discussion reads somewhat qualitatively. Phrases like "relatively high base-level error rates" and "lower in effective per-cell transcript coverage" would benefit from concrete numbers (ONT for example was often previously seen as very error prone but that's no longer the case - more recent papers have these numbers), at least as approximate benchmarks, to help readers appreciate the magnitude of the problems. Citing the systematic benchmarking studies already referenced more specifically here would help.4. Inconsistent Depth Across Biological Applications - The immunology subsection is noticeably more developed than neuroscience and oncology. The neurodevelopment discussion leans heavily on a single study (ScISOr-Seq2), and the oncology discussion is brief. The abstract promises substantial coverage of all three domains; the body of the review should be improved.5. Multi-omics Integration is Underrepresented - Both the abstract and cover letter emphasize multi-omics integration as a key future direction, yet the actual discussion is dispersed and thin. A dedicated subsection or at minimum a summary table of tools like SPLONGGET, ScISOr-ATAC, and scGTP-seq, would make this a more actionable resource for readers planning such experiments.6. Missing Critical Discussion: Benchmarking and Reproducibility - The review mentions benchmark studies but does not synthesise what those benchmarks actually found. A paragraph summarizing key take-home messages from existing benchmarks would substantially increase the practical value of this review.7. RNA Modifications Section is Abrupt - The paragraph introducing m6A-isoSC-seq and scm6A-seq appears mid-section on "Complex Transcripts" and feels appended rather than integrated. RNA modifications arguably deserve either a dedicated subsection or a more deliberate placement with proper framing.

Level of Interest

Please indicate how interesting you found the manuscript: Choose an item.

Quality of Written English

Please indicate the quality of language in the manuscript: Choose an item.

Declaration of Competing Interests

Please complete a declaration of competing interests, considering the following questions:

- Have you in the past five years received reimbursements, fees, funding, or salary from an organisation that may in any way gain or lose financially from the publication of this manuscript, either now or in the future?
- Do you hold any stocks or shares in an organisation that may in any way gain or lose financially from the publication of this manuscript, either now or in the future?
- Do you hold or are you currently applying for any patents relating to the content of the manuscript?
- Have you received reimbursements, fees, funding, or salary from an organization that holds or has applied for patents relating to the content of the manuscript?
- Do you have any other financial competing interests?
- Do you have any non-financial competing interests in relation to this paper?

If you can answer no to all of the above, write 'I declare that I have no competing interests' below. If your reply is yes to any, please give details below.

I hold several patents covering the long-read single-cell and bulk sequencing and hold equity in a biotech that uses long-reads. This will not have an impact on my opinions as a reviewer.

I agree to the open peer review policy of the journal. I understand that my name will be included on my report to the authors and, if the manuscript is accepted for publication, my named report including any attachments I upload will be posted on the website along with the authors' responses. I agree for my report to be made available under an Open Access Creative Commons CC-BY license (http://creativecommons.org/licenses/by/4.0/). I understand that any comments which I do not wish to be included in my named report can be included as confidential comments to the editors, which will not be published.

I agree to the open peer review policy of the journal

To further support our reviewers, we have joined with Publons, where you can gain additional credit to further highlight your hard work (see: https://publons.com/journal/530/gigascience). On publication of this paper, your review will be automatically added to Publons, you can then choose whether or not to claim your Publons credit. I understand this statement.

Yes Choose an item.
