# Supplementary material for: Single-cell long-read transcriptomics: from technologies to biological insights
Source: Gigascience. 2026 Jul 22;15:giag082. doi: 10.1093/gigascience/giag082 (PMC13508701; doi:10.1093/gigascience/giag082)
Supplement: Supplementary file 8 [file giag082_reviewer_2_report_original_submission.pdf]

Reviewer Report

Title: Single-cell Long-read Transcriptomics: From Technologies to Biological Insights

Version: Original Submission **Date:** 5/13/2026

Reviewer name: Reviewer 2

Reviewer Comments to Author:

The manuscript by Ren et al presents an overview of the single cell long reads transcriptomics approaches. The manuscript is timely owing to the rapid development of the technologies and field of applications. Long read transcriptomics has massive potential however as an extremely rapidly developing field it can be difficult to access for a non expert. The availability of reviews of the field including technologies, libraries preparation, and computational approaches can contribute in democratizing such approaches. In the current manuscript the authors present a thorough and well organised overview of long-read single-cell transcriptomics approaches.Nevertheless, the manuscript is currently very verbose and the target audience is unclear, the work is too general / unfocussed for an experienced audience, but includes perhaps more than necessary do the detriment of it's length for non-specialist audience. The manuscript would gain in being substantially shortened and more focused. For instance figures 2 and 3 are rather uniformative for the objective of the manuscript. Those present the sequencing technologies and integration.Likewise the authors should pay close attention to the contents of the tables, as such Table 1 that is focusing on library constructions, also include software and pipelines (Sockeye, scNonoGPS, Flexiplex) obfuscating the message from this table. This is also reflected in the text as lines 232-240 deal with algorithms in a section focusing on scLR-Seq library construction. I invite the authors in bringing clearer structure to the manuscript as this will bring greater focus and potential for the readers.Specific Comments- Line 60. Repeated and meaningless sentence with incorrect citations. Assuming an editing error.- Line 89-92. Has AI been used in this document? This has a language stands out quite differently to the surrounding copy.- Fig1. Editing box around "Allele expression Across Cells". The document has a large number of mistakes and errors like this suggesting closer attention to editing is required.

Level of Interest

Please indicate how interesting you found the manuscript: Choose an item.

Quality of Written English

Please indicate the quality of language in the manuscript: Choose an item.

Declaration of Competing Interests

Please complete a declaration of competing interests, considering the following questions:

- Have you in the past five years received reimbursements, fees, funding, or salary from an organisation that may in any way gain or lose financially from the publication of this manuscript, either now or in the future?
- Do you hold any stocks or shares in an organisation that may in any way gain or lose financially from the publication of this manuscript, either now or in the future?
- Do you hold or are you currently applying for any patents relating to the content of the manuscript?
- Have you received reimbursements, fees, funding, or salary from an organization that holds or has applied for patents relating to the content of the manuscript?
- Do you have any other financial competing interests?
- Do you have any non-financial competing interests in relation to this paper?

If you can answer no to all of the above, write 'I declare that I have no competing interests' below. If your reply is yes to any, please give details below.

I declare that I have no competing interest

I agree to the open peer review policy of the journal. I understand that my name will be included on my report to the authors and, if the manuscript is accepted for publication, my named report including any attachments I upload will be posted on the website along with the authors' responses. I agree for my report to be made available under an Open Access Creative Commons CC-BY license (http://creativecommons.org/licenses/by/4.0/). I understand that any comments which I do not wish to be included in my named report can be included as confidential comments to the editors, which will not be published.

I agree to the open peer review policy of the journal

To further support our reviewers, we have joined with Publons, where you can gain additional credit to further highlight your hard work (see: https://publons.com/journal/530/gigascience). On publication of this paper, your review will be automatically added to Publons, you can then choose whether or not to claim your Publons credit. I understand this statement.

Yes Choose an item.
